# Effects of grazing patterns on grassland biomass and soil environments in China: A meta-analysis

**DOI:** 10.1371/journal.pone.0215223

**Published:** 2019-04-22

**Authors:** Yunqing Hao, Zhengwei He

**Affiliations:** 1 State Key Laboratory of Geohazard Prevention and Geoenvironment Protection, Chengdu University of Technology, Chengdu, China; 2 College of Earth Science, Chengdu University of Technology, Chengdu, China; 3 College of Resources and Environment, Chengdu University of Information Technology, Chengdu, China; Ohio State University South Centers, UNITED STATES

## Abstract

**Background:**

Grazing has important influences on the structures and functions of grassland ecosystems, but the effects of grazing patterns on grassland biomass and soil environments in China remain unclear.

**Objective:**

We employed a meta-analysis to identify the response of biomass and soil environments to different grazing patterns in China.

**Methods:**

Peer-reviewed journal articles were searched using the Web of Science and China National Knowledge to compile a database. A total of 1011 sets of sample observations satisfied the sampling standards; these were derived from 140 study sites and were obtained from 86 published articles. We conducted random effects meta-analyses and calculated correlation coefficients with corresponding 95% confidence intervals.

**Results:**

Grazing significantly decreased the total biomass, aboveground biomass (AGB), belowground biomass (BGB), soil organic matter, soil total nitrogen, soil total phosphorus and soil water content but increased the root-to-shoot ratio, soil available nitrogen, soil pH and bulk density. Generally, increasing grazing intensity and duration significantly increased the effects of grazing on the biomass and soil environment. Additionally, the smallest effects of grazing on the biomass and soil environments were observed under light grazing and cattle grazing alone. Moreover, non-growing season grazing significantly increased AGB, while annual grazing and growing-season grazing significantly reduced AGB. Furthermore, AGB was positively correlated with soil organic matter, soil available phosphorus and bulk density, while BGB was negatively correlated with pH.

**Conclusions:**

These findings highlight the importance of grazing patterns in the biomass and soil environment response to grazing and suggest that cattle grazing alone and grazing during the non-growing season are beneficial for improving the quality of grassland in China.

## Introduction

Grasslands cover 3.55 × 108 hm^2^ in China, accounting for 6% ~ 8% of the world’s grassland area [1]. These areas are important for ecological services [2], such as carbon sequestration, water conservation, and livestock production [3–5]. At present, approximately all grasslands in China are used for grazing and are severely degraded [6]. Grazing is the most important method of grassland utilization and has an important influence on the structures and functions of grassland ecosystems [7, 8]. Grazing can contribute to compensatory growth in grasses. However, overgrazing may result in obvious changes in the composition and structure of the plant community and may lead to a significant decrease in the regenerative ability of grasslands, biomass, and the amount of nutrients returned to the soil through litter, eventually leading to grassland degradation [9].

To understand the effects of grazing on grassland ecosystems, numerous experimental studies have been conducted on grassland vegetation and soil [10, 11], but contradictory results have been obtained. For example, grassland productivity has decreased [11], increased [12], or not changed significantly [13]. The soil environmental response to grazing (such as soil organic matter and available nutrients) also significantly varies among different studies, demonstrating positive, negative or neutral effects [14, 15]. Such a large difference in the results of these studies may be due to large differences in the grazing patterns of the study sites. For instance, compared with light and heavy grazing, intermediate grazing may increase aboveground biomass (AGB) because intermediate grazing results in higher species diversity than no or low grazing conditions [6, 16]. Thus, to understand the response patterns of grassland biomass (AGB or belowground biomass (BGB) or both) and soil environments to different grazing patterns, we must integrate published studies to quantitatively evaluate the effects of different grazing systems on grassland biomass and soil environments.

Grazing is the most important method of grassland utilization, and understanding the impact of grazing patterns on grassland biomass and soil environments is of fundamental importance for grassland conservation and management. Previous comprehensive studies have stressed only the effects of grazing intensity on biodiversity and matter cycling [17–19], but the effects of grazing patterns (including grazing intensity, grazing duration, grazing model and grazing season) on the biomass and soil environments of Chinese grasslands are rarely reported. However, studies on the effects of individual factors on grassland biomass and soil environments are unlikely to reveal the pattern of the effects of grazing on grassland ecosystems. Therefore, a comprehensive analysis and summary of the effects of grazing patterns on grassland biomass and soil environments are required to obtain an in-depth understanding of the response patterns of grassland ecosystems to grazing.

To identify a sustainable grassland management model, we quantitatively analysed the response of the biomass (total biomass, AGB and BGB) and soil environment (physical and chemical properties) of grasslands to different grazing patterns in China. Based on previous studies on the effects of grazing on biomass and soil environments [20–22], we hypothesized that grazing would negatively affect the biomass and soil environments of grasslands in China, and the response of target variables to grazing can be regulated by grazing intensity, grazing duration, grazing model and grazing season. To test this hypothesis, we addressed the following questions: (1) What are the effects of grazing on grassland biomass (i.e., total biomass, AGB and BGB)? (2) What is the response pattern of the physicochemical properties of grassland soil to grazing? (3) Do the different grazing patterns (including grazing intensity, grazing duration, grazing model and grazing season) regulate the response of the grassland biomass and soil environment to grazing?

## Materials and methods

### Data sources and compilation

Peer-reviewed journal articles were searched using the Web of Science and China National Knowledge to compile a database. The publication time of all literature in the database was limited to 2007–2018. The following key words were used to select the studies: grazing, grassland biomass, grassland productivity, and soil environment. To reduce publication bias, previous case studies had to satisfy the following criteria to be selected for analyses: (1) the experimental data must be collected from manipulative field experiments on grazing in Chinese grasslands; (2) the experiments had to include grazing treatments and a control treatment, and the grazing patterns were clearly indicated in the grazing treatment (i.e., grazing intensity, grazing duration, grazing model and grazing season); (3) the experimental data of the biomass and soil environments were measured during the peak growing season; (4) the means, standard deviations (SDs) and/or standard errors (SEs), and confidence interval of the grazing treatments and controls were presented in each primary study; (5) the soil depth measured for the belowground biomass data had to exceed 30 cm; and (6) seasonal grazing had to last at least two months. Additionally, we collected the background information relevant to the data from the papers for detailed analyses and comparisons. For instance, the grazing intensities, grazing duration, grazing season, and grazing models were collected from journal articles. We extracted the raw data directly from tables of the published reports or used the Get Data Graph Digitizer (version 2.24, Russian Federation) to extract data from digitized graphs. Consequently, 1011 sets of sample observations satisfied the sampling standards; these were derived from 140 study sites (S1 Fig) and were obtained from 86 published articles (S2 Fig, S1 Table). To determine how grazing patterns regulated the effects of grazing on grassland ecosystems, we grouped the data according to grazing intensity (i.e., light grazing, moderate grazing, heavy grazing, extreme grazing), livestock species grazing models (i.e., sheep grazing alone, cattle grazing alone, mixed sheep and cattle grazing), grazing season (i.e., annual grazing, growing-season grazing, non-growing season grazing), and grazing duration (≤ 1 year, 2–5 years, > 5 years) based on previous studies [5, 6].

### Meta-analysis

The data were analysed using a meta-analysis approach, which was used to calculate the response ratios (RRs) of the biomass and soil environments to grazing [12]. The RR is defined as the natural log of the ratio of the mean value of a specific variable in the experimental grazing group (*Xe*) to that in the control group (*Xc*), which can represent the magnitude of changes in the target variables (including biomass and soil physical and chemical properties) [9]. The formula is as follows:

RRs=ln(Xe/Xc)=ln(Xe)-ln(Xc) (1)

where *Xe* and *Xc* are the average values of the respective experimental group and control group in an independent study. If both *Xe* and *Xc* are normally distributed and *X*c is not equal to zero, the RRs are also approximately normally distributed. The variance (*V*) of the RRs is estimated by:

$$V={Se}^{2}/(Ne,\;{Xe}^{2})+{Se}^{2}/\left(Nc,{Xc}^{2}\right)$$ (2)

where Ne and Nc represent the sample sizes, and Se and Sc are the SDs of the concerned variable in the experimental and control groups, respectively. The reciprocal of variance (W = 1/V) was considered as the weight (W) of each RR. A mixed model was conducted using the meta-analytical software [23] Meta-win 2.1 (Sinauer Associates, Inc. Sunderland, MA, USA) to calculate the weighted RRs and the 95% confidence interval (95% CI). The 95% CI was also used to test whether the weighted RR of a grazing treatment was significant for a specific variable. If the bounds of the 95% CI of the RR overlapped with 0, the RR of the variable for treatment was not significant. If the ends of the 95% CI were greater or lower than 0, the RR of the variable for the treatment was significant. The total heterogeneity of a group of comparisons (QT) was partitioned into within-group (Qw) and between-group (Qb) heterogeneity. The Qb statistic can be tested against a Chi-squared distribution with k–1 degrees of freedom. A significant Qb suggests that there are differences among cumulative effect sizes of the groups [23].

### Publication bias

Publication bias is a potential threat to the validity of all meta-analysis studies [24]. We evaluated the presence of possible publication bias using a histogram (S3 Fig, the AGB, BGB, root-to-shoot ratio, soil available nitrogen, available phosphorus and organic matter were cited). A curve fitted by a Gaussian function was used to estimate the publication bias based on previous studies [25]. The histogram of the response of each variable to grazing followed a normal distribution (S3 Fig), suggesting that there was no publication bias in our results.

The relationship between the RRs of the biomasses and the soil environments was examined. Specifically, correlation analyses were used to analyse the relationships between the response ratios of the AGB, BGB and total biomass and the soil total nitrogen concentration, soil total phosphorus concentration, soil available nitrogen, soil available phosphorus, soil organic matter, soil water content, pH and soil bulk density, respectively. SPSS software (SPSS 17.0 for Windows; SPSS Inc; Chicago, IL, USA) was used to conduct the correlation analysis, and Origin (version 8.0) was used to draw the graphs.

## Results

### Effects of grazing on biomass and soil environments

**Fig 1:**
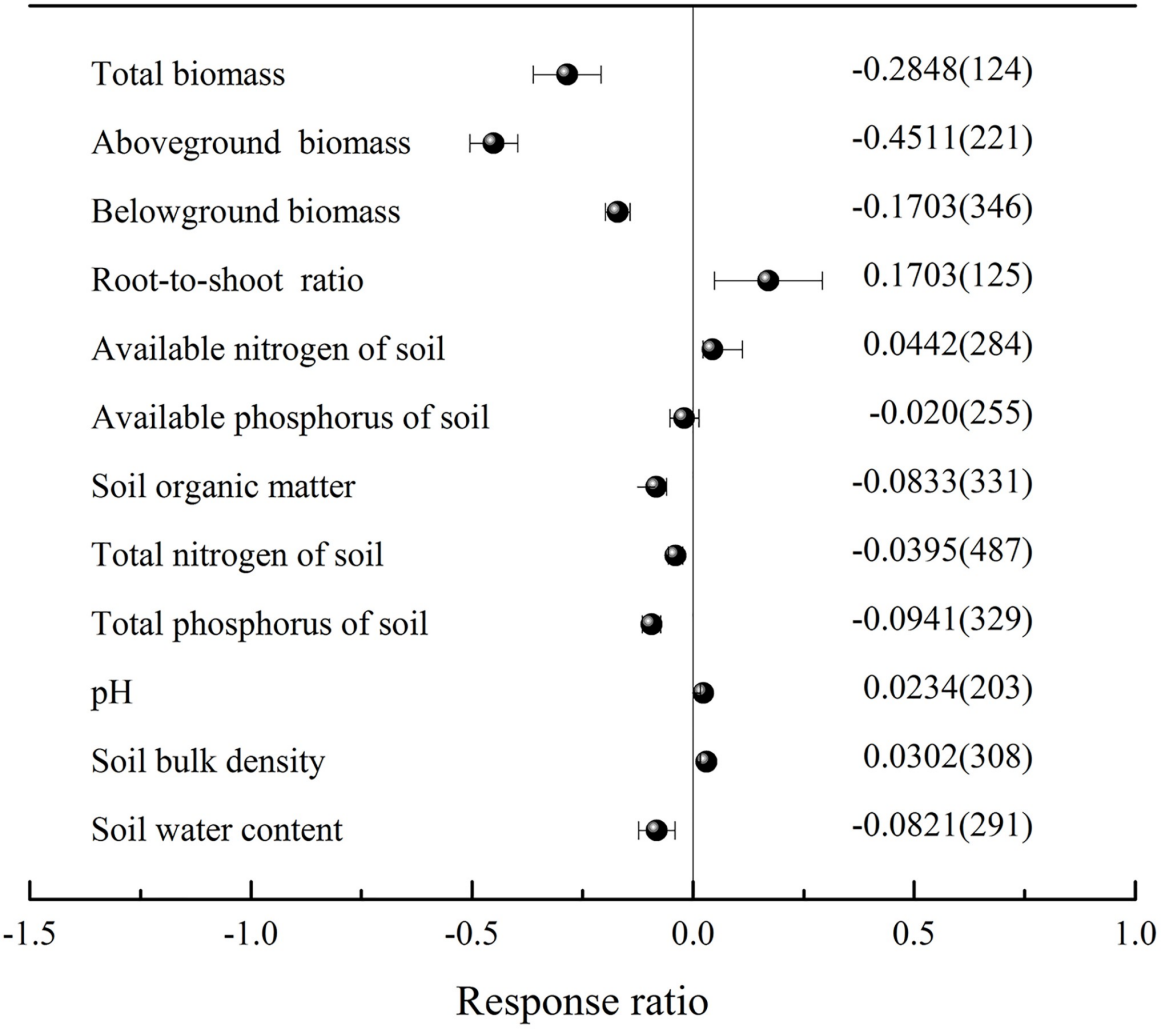
The effect size of grassland biomass and soil environments in response to grazing. Error bars are the 95% bootstrapped confidence intervals. The numbers inside and outside the parentheses indicate the response ratio (RR) and the number of observation samples, respectively.

Across all studies, the total biomass, AGB and BGB of grazing grassland decreased significantly by an average of 28.48%, 45.11% and 17.03%, respectively, while the root-to-shoot ratios increased significantly by 17.03% (Fig 1). In addition, grazing significantly decreased soil organic matter (8.33%), total nitrogen (3.95%), total phosphorus (9.41%) and the soil water content (8.21%) but increased the soil available nitrogen (4.42%), pH (2.34%) and bulk density (3.02%). Furthermore, grazing had no significant effects on soil available phosphorus (Fig 1).

### Grazing patterns influence the effects of grazing on biomass

**Fig 2:**
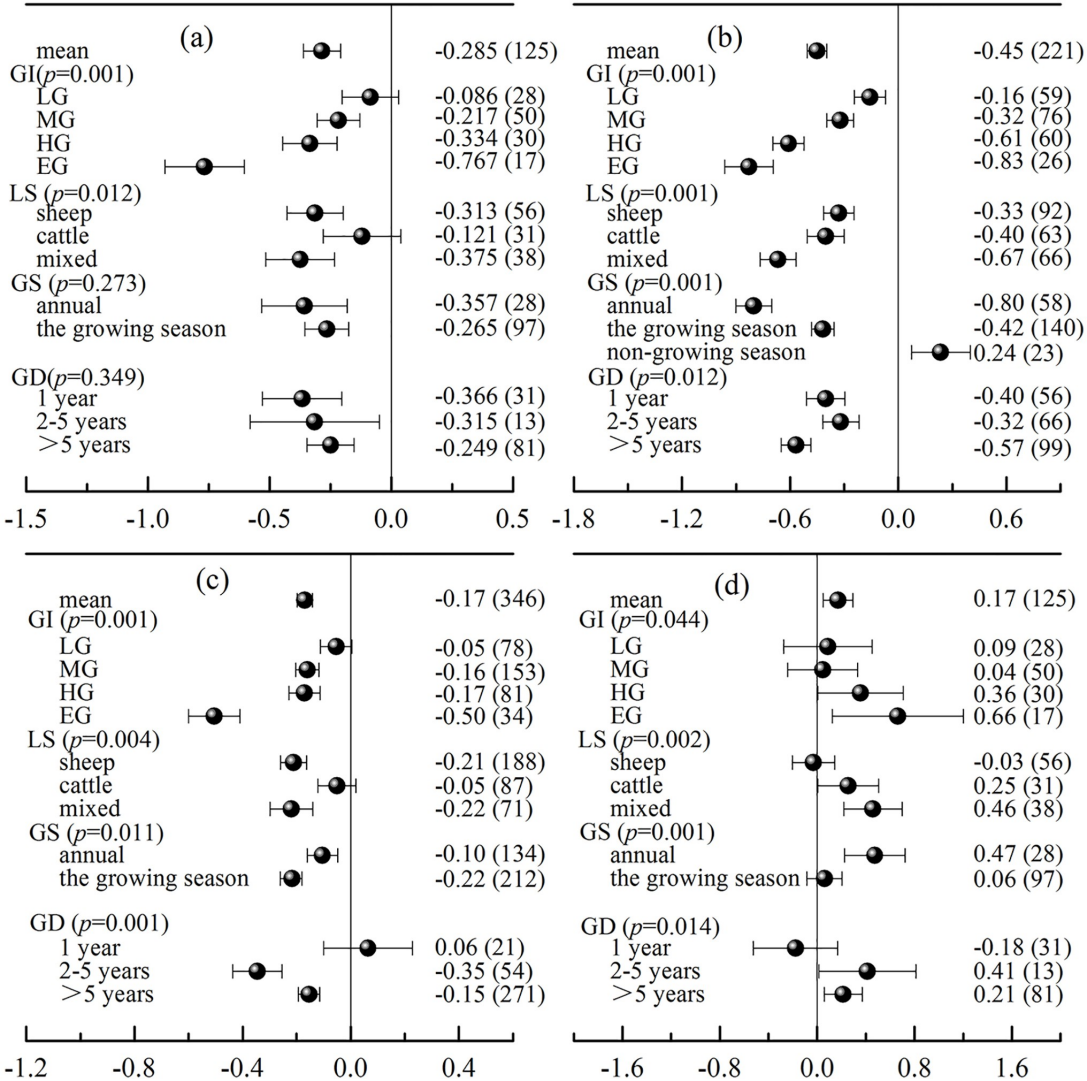
The effects of grazing intensity (GI), livestock species grazing models (LS), grazing season (GS) and grazing duration (GD) on total biomass (a), aboveground biomass (b), belowground biomass (c) and the root-to-shoot ratio (d). Error bars are the 95% bootstrapped confidence intervals. The numbers inside and outside the parentheses indicate the response ratio (RR) and the number of observation samples, respectively. LG = light grazing; MG = moderate grazing; HG = heavy grazing; EG = extreme grazing.

Across all the studies, grazing had a significant negative effect on grassland biomass (i.e., total biomass, AGB, BGB). However, the intensity of this negative effect varied with the grazing patterns (Fig 2, Table 1); as the intensity of grazing increased, the negative response of biomass (total biomass, AGB, BGB) to grazing increased. For example, light grazing (LG) slightly affected the biomass, while other grazing intensities (moderate grazing, heavy grazing, and extreme grazing) had significant negative effects on total biomass, AGB and BGB (all *p* < 0.001). In addition, when the data were classified according to the type of livestock, there were significant differences among the subgroups (*p* < 0.001). The smallest observed effects on total biomass were observed in response to cattle grazing alone. Moreover, we found that non-growing season grazing had a significant positive effect on AGB, with an increase of 24% (*p* < 0.05, total biomass and BGB data were not collected). Conversely, annual grazing and growing-season grazing had significant negative effects on AGB, BGB and total biomass (all *p* < 0.05). Furthermore, when the grazing duration was considered, we found that BGB decreased significantly by 35% and 15% based on study durations of 2–5 years and > 5 years, respectively, but there was no significant effect on BGB for study durations of 1 year (Fig 2c). When the grazing duration increased from short to long (≤ 1 year, 2–5 years, > 5 years), AGB decreased significantly by 40%, 32% and 57%, respectively (*p* = 0.012, Fig 2b).

**Table 1:** Between-group heterogeneity (Qb) among the studies comprising each response variable.

	Response variables	N	Grazing intensity	Livestock species grazing models	Grazing season	Grazing duration
Biomass	Total biomass	124	Qb = 54.92**	Qb = 6.31*	Qb = 0.91	Qb = 1.64
	AGB	221	Qb = 179.26**	Qb = 27.85**	Qb = 127.91**	Qb = 15.65*
	BGB	346	Qb = 67.88**	Qb = 15.90**	Qb = 9.25*	Qb = 24.28*
	Root-to-shoot ratio	125	Qb = 5.70*	Qb = 11.62**	Qb = 8.57**	Qb = 6.22**
Soil physical and chemical properties	Soil available nitrogen	284	Qb = 6.40	Qb = 36.98**	Qb = 82.02**	Qb = 9.69
	Soil available phosphorus	255	Qb = 16.62*	Qb = 4.67	Qb = 1.43	Qb = 3.10
	Soil organic matter	331	Qb = 12.70*	Qb = 92.25**	Qb = 23.37**	Qb = 20.51**
	Soil total nitrogen	487	Qb = 6.02*	Qb = 11.00*	Qb = 4.19*	Qb = 13.49*
	Soil total phosphorus	329	Qb = 15.44**	Qb = 2.56	Qb = 23.69**	Qb = 3.46
	pH	203	Qb = 15.13**	Qb = 49.89**	Qb = 10.39*	Qb = 54.46**
	Soil bulk density	308	Qb = 30.96**	Qb = 61.02**	Qb = 5.11	Qb = 47.56**
	Soil water content	291	Qb = 13.48*	Qb = 13.46**	Qb = 18.66**	Qb = 16.48**

Note:

** indicates *p* < 0.01;

* indicates *p* < 0.05.

Grazing patterns not only affected grassland biomass but also affected the allocation of above- and belowground material (Fig 2d). The response ratios of the root-to-shoot ratios increased with intensified grazing. The results show that high grazing intensity led to a larger proportion of belowground material allocation. Additionally, when cattle and sheep grazed separately, there was no significant effect on the root-to-shoot ratios, but when cattle and sheep grazed together, the root-to-shoot ratio increased significantly by 46% (*p* < 0.05). Moreover, with respect to seasonal grazing, growth-season grazing had no significant effect on the root-to-shoot ratio, but annual grazing significantly increased the root-to-shoot ratio by 47% (*p* < 0.05). Furthermore, continuous grazing for more than 2 years (2–5 years, > 5 years) also significantly increased the root-to-shoot ratio (*p* < 0.05, Fig 2d).

### Grazing patterns influence the effects of grazing on soil environments

**Fig 3:**
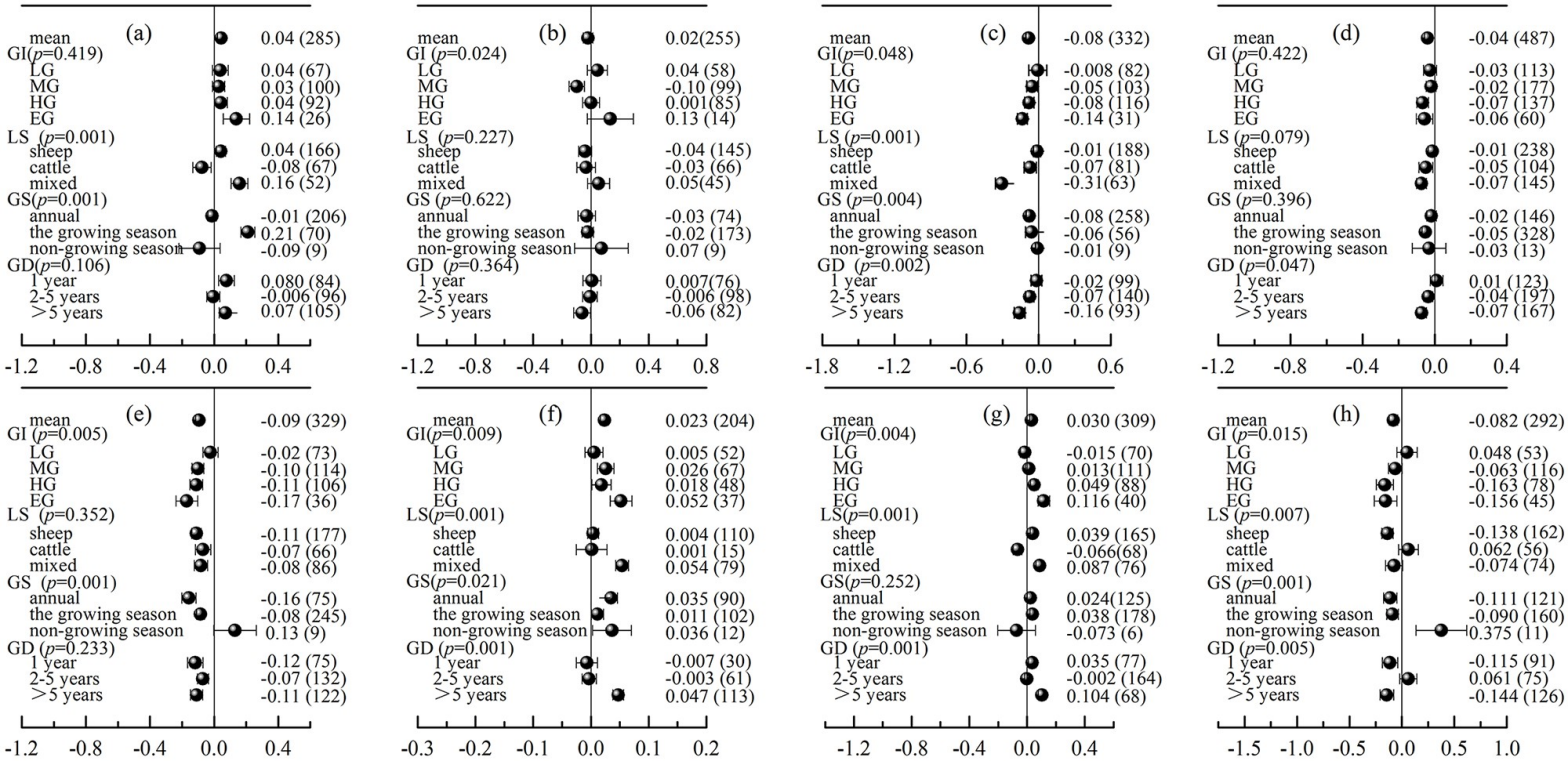
The effects of grazing intensity (GI), livestock species grazing models (LS), grazing season (GS) and grazing duration (GD) on soil available nitrogen (a), available phosphorus (b), organic matter (c), total nitrogen (d), total phosphorus (e), pH (f), density (g) and water content (h). Error bars are the 95% bootstrapped confidence intervals. The numbers inside and outside the parentheses indicate the response ratio (RR) and the number of observation samples, respectively. LG = light grazing; MG = moderate grazing; HG = heavy grazing; EG = extreme grazing.

The results showed that the response of the soil physical and chemical properties to grazing varied with the grazing patterns (Fig 3, Table 1). For example, LG had the lowest impact on the physical and chemical properties of grassland soil, and there was no significant impact on all observation indexes. However, the responses of soil characteristics to grazing increased with intensified grazing. There was a positive response of soil available nitrogen, pH and bulk density to grazing, while grazing had a negative impact on the soil total nitrogen, total phosphorus, available phosphorus, organic matter and soil water content. Additionally, when the data were subdivided based on the type of grazing livestock, the responses of the soil water content, available nitrogen and bulk density to grazing differed significantly between cattle grazing alone or sheep grazing alone and mixed grazing (*p* < 0.05). We found that the greatest effect sizes of grazing on the soil environment were observed under mixed cattle and sheep grazing, which had significant effects on the soil available nitrogen (16%), organic matter (-31%), total nitrogen (-7%), total phosphorus (-17%), pH (5.2%), and bulk density (8.7%) (Fig 3). Cattle grazing alone significantly increased the soil water content but reduced the soil available nitrogen and bulk density. However, the opposite result was found for sheep grazing alone and mixed grazing (Fig 3a, 3g and 3h). Furthermore, when considering the grazing seasons, non-growing season grazing had a significant positive effect on the soil moisture content (37.5%), while annual grazing and growing season grazing had a significant negative effect on the soil water content (-11.1% and -9%, respectively, Fig 3h). Finally, in addition to the pH and available nitrogen, there was a decreasing tendency in most indicators with increased grazing duration (Fig 3c and 3d).

### Correlation analysis

The response ratio of biomass changed with the soil environmental factors. For example, correlation analysis of biomass and soil physical and chemical properties showed that AGB was positively correlated with soil organic matter (correlation coefficient = 0.594, *p* = 0.002), soil available phosphorus (correlation coefficient = 0.518, *p* = 0.002) and soil bulk density (correlation coefficient = 0.25, *p* = 0.034). However, there was a significant negative correlation between BGB and soil pH (correlation coefficient = -0.551, *p* = 0.017) (Table 2).

**Table 2:** Correlations between biomass (AGB, BGB) and soil environmental factors (total nitrogen, total phosphorus, available nitrogen, available phosphorus, soil organic matter, water content, pH, bulk density).

	Total nitrogen	Total phosphorus	Available nitrogen	Available phosphorus	Soil organic matter	Water content	pH	Bulk density
AGB	0.055	0.077	0.176	0.518 *	0.594 *	0.038	0.254	0.25 *
	*p =* 0.349	*p* = 0.333	*p* = 0.181	*p* = 0.002	*p* = 0.002	*p* = 0.402	*p* = 0.115	*p* = 0.034
	N = 54	N = 34	N = 29	N = 28	N = 21	N = 46	N = 24	N = 54
BGB	0.187	0.233	0.186	0.422	—	0.179	0.551 *	0.102
	*p* = 0.236	*p* = 0.234	*p* = 0.303	*p* = 0.129	—	*p* = 0.186	*p* = 0.017	*p* = 0.263
	N = 17	N = 12	N = 10	N = 9	—	N = 27	N = 15	N = 41

* indicates *p* < 0.05.

## Discussion

### Effects of grazing on grassland

Grazing is one of the primary models in the utilization of grasslands worldwide. Livestock have direct and indirect impacts on AGB and BGB, as well as on the C distribution of vegetation above- and belowground. Milchunas and Lauenroth (1993) assessed the effects of grazing on grassland biomass at a global scale. The results showed that grazing did not significantly affect the total biomass of grassland but decreased AGB by 23% and increased BGB by 20% [13]. However, the results presented here showed that grazing significantly decreased the total biomass, AGB and BGB by 28.4%, 45% and 17.03%, respectively, and thus, the root-to-shoot ratio increased by 17.03% (Fig 1). Obviously, the results in our study are different from those found at the global scale, which are similar to the national scale syntheses for China [6]. Based on a previous study of grazing grasslands in China, grazing significantly decreased the total biomass, AGB and BGB by 58.34%, 42.77%, and 23.13%, respectively, and significantly increased the root-to-shoot ratio by 30.58% [6]. However, the effect sizes of grazing on the target variables in our study were lower than those reported in a previous study. This discrepancy may be attributable to the previous study having a smaller amount of data, i.e., only 12 sets of total biomass data, 80 sets of AGB data, 108 sets of BGB data, and all these data were from Inner Mongolia. The results of our study showed that grazing had a significant negative effect on total biomass, AGB and BGB, and the following factors may explain this phenomenon. First, AGB was significantly reduced by livestock feeding, which will decrease root elongation and biomass [26]. Second, the effects of herbivory on the biomass (AGB, BGB and total biomass) may directly reduce the source of soil organic matter and indirectly affect the decomposition of litter by changing soil environmental conditions (such as soil temperature, aeration and insolation) [27]. These effects of grazing may result in soil coarsening and a loss of soil organic matter and nutrients, which in turn negatively affect grassland biomass (AGB, BGB and total biomass), ultimately resulting in grassland degradation. Furthermore, although the total amount of BGB decreases with grassland degradation, to obtain more nutrients for growth, plants will allocate a higher proportion of biomass belowground [28], which is consistent with our results (Fig 1). Therefore, these results indicate that grassland productivity in China is seriously affected by grazing, and the degradation will be further aggravated if the current grazing patterns remain.

Grazing not only affects the biomass of grassland vegetation and the allocation of C between above- and belowground stocks but also directly or indirectly affects the physical and chemical properties of soil [29]. For example, we found that grazing decreased the soil organic matter, soil available phosphorus, total nitrogen, and total phosphorus (Fig 1), and these results can be explained by the following reasons. First, in the short term, plant defoliation often increases C-rich root exudates to stimulate microbial activity and turnover, ultimately resulting in an increase in soil nutrients available to plants [30]. However, in the longer term, herbivores feed on the aboveground biomass in grasslands, which reduces plant coverage and litter production [31] and is an important means through which the formation of soil organic matter is affected. Second, accelerated erosion caused by wind and water due to decreased vegetation cover and litter ultimately results in soil coarsening and loss of soil organic matter and nutrients [32]. In addition, the results showed that grazing increases soil bulk density (Fig 1) through the formation of soil peds [33] and reduces soil infiltration [32]. For instance, as grazing intensity increases, the increase in bulk density leads to a decrease in hydraulic conductivity and water repellency and an increase in the penetration time of water droplets [34], which is a negative feedback response to grazing from an environmental viewpoint [35]. These processes may lead to increased erosion, with an associated loss of nutrients and decreased plant available water [33], similar to our results (Fig 1). Furthermore, grazing significantly increased the soil available nitrogen concentration, which can be attributed to ungulate waste and its facilitative effect on soil biological processes, and is commonly promoted as one of the main driving mechanisms for grazers stimulating nitrogen availability [36]. Herbivorous mammals can return large quantities of undigested and non-assimilated nutrients to the soil as dung and urine, and this waste directly increases the soil available nitrogen [37]. Approximately 75–90% of the nitrogen intake of grazing animals is returned to the soil as excreta [38]. Excretal return effectively shortcuts the litter-decomposition pathway, providing highly decomposable resources that are rich in labile nutrients that can stimulate microbial biomass and activity [39], net carbon and nitrogen mineralization [40], and facilitate litter decomposition. In general, grazing decreases the source of soil organic matter, facilitates litter decomposition, and increases leaching to reduce soil organic matter. Additionally, grazing also decreases the density of soil organic matter and the water-holding capacity compared with mineral material, which contributes to the soil cation exchange capacity [41]. Moreover, the grazing process leads to the loss of soil nutrients, but animal faeces and urine increase the soil available nitrogen [36] and pH [42, 43].

In addition to these phenomena, we also found that AGB was positively correlated with soil organic matter (Table 2), which is consistent with previous research [44]. Soil organic matter is an important index for measuring soil quality [45] and plays an important role in cation retention and in regulating the availability of nutrients [46]. However, as described above, grazing can decrease the amount of soil organic matter input by reducing aboveground organisms [47]. A previous study as also showed that soil organic matter is significantly positively correlated with soil nutrients [48]. Thus, poor soil organic matter indicates a lack of soil nutrients, which negatively affects AGB and may result in grassland degradation. However, there was a positive relationship between AGB and bulk density in our results (Table 2). This phenomenon may be explained by the increase in unpalatable forbs in grassland, which is caused by overgrazing and indicates grassland degradation [49]. Similar results were found in research conducted in the source region of the Yellow River in China, which showed that soil bulk density increased as land degradation worsened, and aboveground biomass increased on extremely degraded land with the highest amount of inedible aboveground biomass for livestock [50]. Thus, taken together, these results indicate that the soil organic matter content can be used as an indicator to quantify soil nutrient and grassland degradation. These results indicate that grazing has a significant negative effect on the growth of plants and the soil of grasslands and may be one of the main causes of grassland degradation in China. Therefore, understanding the effects of grazing on grassland biomass and the soil environment is an important way to improve the negative impacts of grazing on grassland ecosystems and realize sustainable resource management and development.

### Factors influencing the effects of grazing on grassland

Analysis using all data showed that the RRs of grassland biomass and the soil environment to grazing were affected by different grazing patterns (Figs 2 and 3). As discussed above, grazing can significantly decrease the biomass of grassland. The negative RRs of the total biomass, AGB and BGB to grazing increased with increasing grazing intensity (Fig 2a and 2b). AGB was reduced by livestock feeding. As important photosynthetic components, the loss of AGB will lead to a reduction in the photosynthetic products of plants and thus to the redistribution of belowground C to the aboveground components [51], which further results in a reduction of BGB. As a result, a high grazing intensity led to a higher reduction of AGB, BGB and total biomass compared with light grazing (Fig 2c, *p* < 0.001). In addition, more nutrients are needed for plant regeneration when grazing intensity increases, which causes the proportion of BGB relative to total biomass to increase, thus allowing the acquisition of more nutrients [6, 51]. Therefore, as grazing intensity increased, the root-to-shoot ratio increased (Fig 2d). Moreover, grazing may result in obvious changes in the soil environment. The effects of grazing on soil organic matter, total nitrogen and total phosphorus increased with increasing grazing intensity (Fig 3c, 3d and 3e). This result may have occurred because grazing significantly decreased the return of litter, resulting in a decrease in soil nutrient contents [52]. Although livestock can enhance available nutrients (such as soil effective nitrogen and phosphorus) in grassland soil through faeces [12] (Fig 3a and 3b), livestock has a strong trampling effect on the soil, resulting in increased soil bulk density and pH and a reduced soil water content [53] (Fig 3g, 3f and 3h).

Sheep and cattle are the principal grazing animals in Chinese grasslands. We classified the grazing system according to the type of grazing livestock (sheep, cattle and mixed) and found that the effects of grazing on grassland biomass and the soil environment differed significantly under different grazing models. The effects of mixed cattle and sheep grazing on grassland biomass and soil environments were greater than those of cattle grazing alone or sheep grazing alone (Figs 2 and 3). It is worth noting that the effect of sheep grazing alone on grassland biomass and soil environments was more pronounced than that of cattle grazing alone, especially in terms of biomass. This phenomenon can be explained by the fact that the feeding habits of cattle and sheep are different [54, 55]. For example, the feed intake of cattle is high, but there is no requirement for pasture quality [56]. However, compared with cattle, sheep have higher selectivity in terms of eating and prefer to choose grass that is close to the ground [54]. At present, the grasslands of China have been largely degraded by overgrazing [57]. Due to the poor quality and community structure of grasslands, it has become more difficult for livestock to harvest gramineous plants, especially for cattle. Compared with sheep, cattle have a smaller proportion of the AGB to feed on; thus, they have a smaller influence on the biomass of plants. In addition, mixed cattle and sheep grazing will increase the utilization rate of grassland [58], which may also increase the grazing pressure on grassland.

Our results showed that the effects of grazing on grassland biomass and the soil environment differed significantly in different grazing seasons (Fig 2b and 2c). Notably, non-growing season grazing significantly increased AGB (Fig 2b). Three mechanisms may support this phenomenon. The first is that non-growing season grazing significantly increased the soil water content (Fig 3h), which is the main limiting factor for the growth of grassland plants [59]. In addition, non-growing season grazing may promote seed dispersal and increase seed sources [60]. For example, grazing animals can expand the spread of seeds and promote seed germination by forming nutrient patches through their excrement [61]. Third, trampling by grazing animals leads to the reduction of litter and shallow placement of seeds, which is conducive to the germination and growth of plants in the following year [62].

Our results also showed different responses of the biomass and soil environments of grasslands to different grazing durations, especially in terms of the belowground biomass, soil organic matter, total nitrogen, pH and bulk density (all *p* = 0.001). Moreover, the effects of grazing on grassland increased with increasing grazing duration. A grazing duration of 1 year had no significant influence on the biomass, soil organic matter, total nitrogen, pH or bulk density. However, grazing duration > 5 years significantly reduced BGB (15%), soil organic matter (16%) and total nitrogen (7%) but significantly increased soil pH (4.7%) and bulk density (10.4%).

## Conclusions

In summary, this study employed a meta-analysis to identify the response of biomass and soil environments to different grazing patterns in China. In general, grazing significantly decreased the total biomass, AGB, BGB, soil organic matter, soil total nitrogen, soil total phosphorus and soil water content but clearly increased the root-to-shoot ratio, soil effective nitrogen, soil pH and bulk density. Generally, increasing the grazing intensity and duration significantly increased the effects of grazing on the biomass and soil environment. Additionally, the smallest effects of grazing on the biomass and soil environment were observed under light grazing and cattle grazing alone compared with sheep grazing alone and mixed grazing. Furthermore, in the non-growing season, grazing significantly increased AGB, while annual grazing and growing-season grazing significantly reduced AGB. The results also showed that AGB was positively correlated with soil organic matter, soil available phosphorus and soil bulk density, while BGB was negatively correlated with soil pH. Therefore, different grazing patterns regulate the response of the grassland biomass and soil environment to grazing.

## Supporting information

S1 Fig The distribution of studies sites included in the meta-analysis. (TIF)

S2 Fig The flow diagram. Peer-reviewed journal articles were searched using the Web of Science and China National Knowledge to compile a database. The publication time of all literature in the database was limited to 2007–2018. The following key words were used to select the studies: grazing, grassland biomass, grassland productivity, and soil environment. Finally, 86 literatures were selected from 774 literatures.
(TIF)

S3 Fig The histogram of the response of the aboveground biomass (a), belowground biomass (b), root-to-shoot ratio (c), available nitrogen of soil (d), soil available phosphorus of soil (e) and organic matter (f) to grazing. (TIF)

S1 Table The papers used in this study to establish the database of the effects of different marketer grazing systems on grassland biomass and soil environments. (DOCX)

S1 Checklist PRISMA checklist. (DOC)
