# Supplementary figures and images for: Effects of grazing patterns on grassland biomass and soil environments in China: A meta-analysis
Source: PLoS One. 2019 Apr 22;14(4):e0215223. doi: 10.1371/journal.pone.0215223 (PMC6476490; doi:10.1371/journal.pone.0215223)

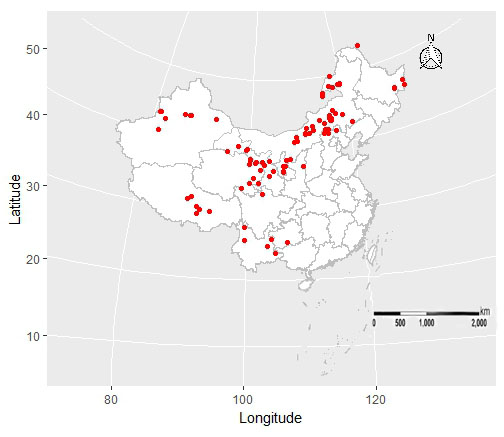

Supplement: S1 Fig — (TIF) [file pone.0215223.s001.tif]

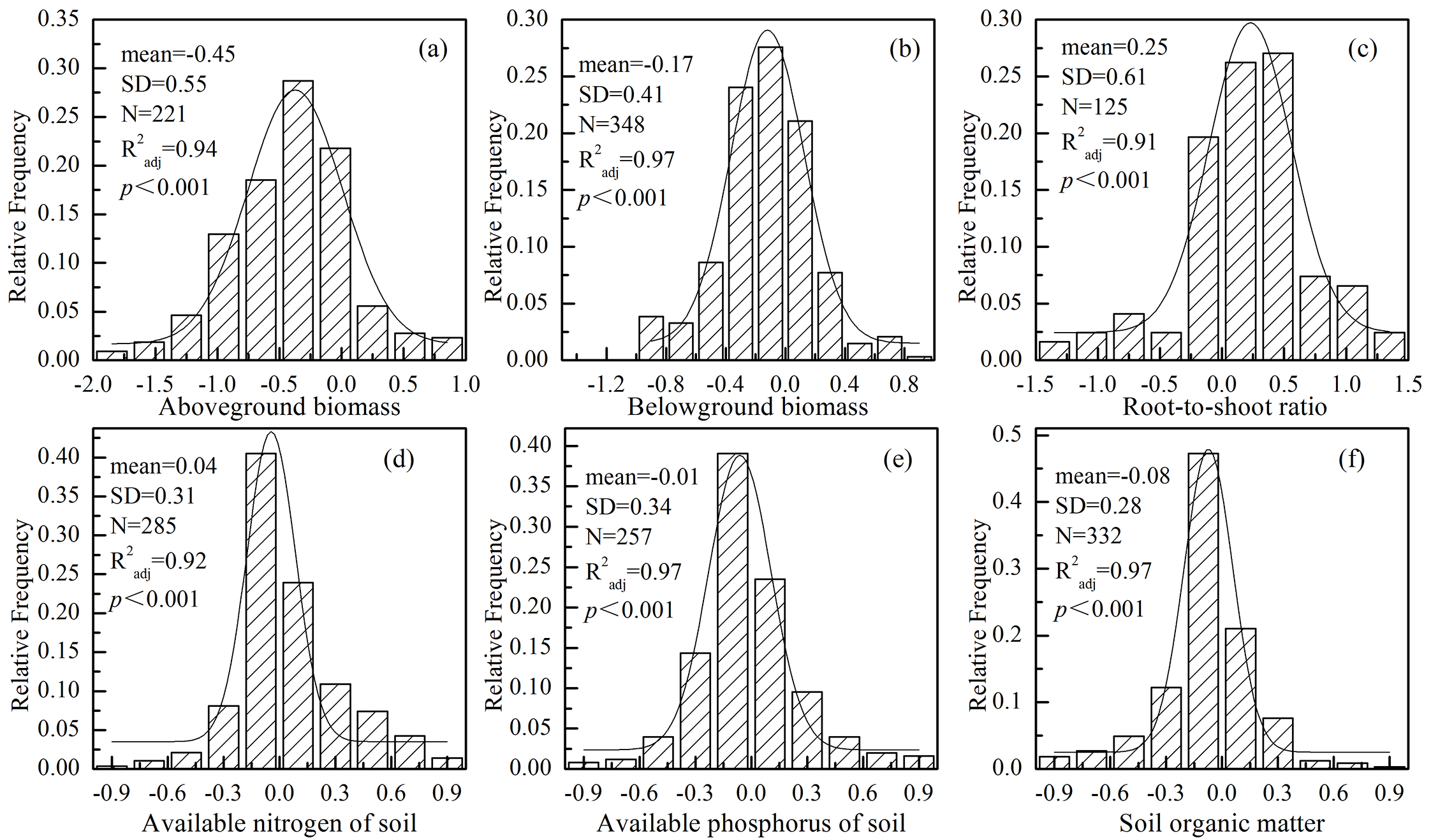

Supplement: S3 Fig — (TIF) [file pone.0215223.s003.tif]
