# Supplementary material for: Effects of grazing patterns on grassland biomass and soil environments in China: A meta-analysis
Source: PLoS One. 2019 Apr 22;14(4):e0215223. doi: 10.1371/journal.pone.0215223 (PMC6476490; doi:10.1371/journal.pone.0215223)
Supplement: S1 Table — (DOCX) [file pone.0215223.s004.docx]

**S1 Table. The papers used in this study to establish the database of the effects of different marketer grazing systems on grassland biomass and soil environments**

|  | grazing systems | | | |  | biomass | | | | | soil environments | | | | | | | |
| --- | --- | --- | --- | --- | --- | --- | --- | --- | --- | --- | --- | --- | --- | --- | --- | --- | --- | --- |
| References | Durations | livestock species | grazing season | grazing intensity | Times | AGB | BGB | Root-to-shoot ratio | | total biomass | total nitrogen | total phosphorus | available nitrogen | available phosphorus | organic matter | pH | density | water content |
| 1 | ＞5 years | sheep | the growing season | LG | 3 |  |  |  |  | |  |  | -0.3677 |  |  | 0.0324 |  | 0.0927 |
| 1 | ＞5 years | sheep | the growing season | MG | 3 |  |  |  |  | |  |  | -1.8718 |  |  | 0.0348 |  | -0.0379 |
| 1 | ＞5 years | sheep | the growing season | HG | 3 |  |  |  |  | |  |  | -0.9555 |  |  | 0.0275 |  | -0.1117 |
| 2 | ＞5 years | mixed | annual | EG | 3 |  |  |  |  | | -0.5561 | -0.0292 | -0.168 |  |  | 0.096 | 1.0498 | -0.4143 |
| 2 | ＞5 years | mixed | annual | EG | 3 |  |  |  |  | | -0.7114 | -0.3884 | -0.4778 |  |  | 0.1252 | 1.0826 | -0.4253 |
| 3 | 2-5 years | sheep | the growing season | LG | 3 |  |  |  |  | | 0.3489 | 0.1872 |  |  | 0.252 | -0.01 |  |  |
| 3 | 2-5 years | sheep | the growing season | LG | 3 |  |  |  |  | | 0.0924 | -0.0625 |  |  | -0.1077 | -0.035 |  |  |
| 3 | 2-5 years | sheep | the growing season | HG | 3 |  |  |  |  | | 0.2556 | 0.1625 |  |  | 0.182 | -0.0354 |  |  |
| 3 | 2-5 years | sheep | the growing season | HG | 3 |  |  |  |  | | 0.1769 | 0.087 |  |  | 0.1061 | -0.0143 |  |  |
| 4 | 2-5 years | sheep | the growing season | HG | 3 |  |  |  |  | |  |  |  |  |  |  | 0 |  |
| 4 | 2-5 years | sheep | the growing season | HG | 3 |  |  |  |  | |  |  |  |  |  |  | -0.0163 |  |
| 4 | 2-5 years | sheep | the growing season | HG | 3 |  |  |  |  | |  |  |  |  |  |  | 0.016 |  |
| 5 | 2-5 years | sheep | the growing season | EG | 3 | -1.189 | -0.1379 | 1.0511 | -0.5622 | | 0.1427 |  | 0.3217 |  |  |  |  |  |
| 5 | 2-5 years | sheep | the growing season | EG | 3 |  |  |  |  | | 0.2768 |  | 0.7512 |  |  |  |  |  |
| 5 | 2-5 years | cattle | the growing season | EG | 3 | -1.0338 | -0.0233 | 1.0105 | -0.4359 | | 0.1374 |  | -0.3892 |  |  |  |  |  |
| 5 | 2-5 years | cattle | the growing season | EG | 3 |  |  |  |  | | -0.0146 |  | 0.6374 |  |  |  |  |  |
| 5 | 2-5 years | mixed | the growing season | EG | 3 | -1.403 | -0.2195 | 1.1835 | -0.6796 | | 0.1103 |  | 0.4918 |  |  |  |  |  |
| 5 | 2-5 years | mixed | the growing season | EG | 3 |  |  |  |  | | 0.2768 |  | 0.0916 |  |  |  |  |  |
| 6 | 2-5 years | sheep | non-growing season | LG | 3 | 0.1915 |  |  |  | |  |  |  |  |  |  |  |  |
| 6 | 2-5 years | sheep | non-growing season | MG | 3 | 0.2495 |  |  |  | |  |  |  |  |  |  |  |  |
| 6 | 2-5 years | sheep | non-growing season | HG | 3 | 0.0582 |  |  |  | |  |  |  |  |  |  |  |  |
| 7 | 1 year | sheep | the growing season | LG | 3 |  |  |  |  | | 0.4796 |  |  |  | 0.361 | -0.024 | -0.0299 |  |
| 7 | 1 year | sheep | the growing season | LG | 3 |  |  |  |  | | 0 |  |  |  | 0.2056 | -0.0059 | 0.0385 |  |
| 7 | 1 year | sheep | the growing season | LG | 3 |  |  |  |  | | 0.3254 |  |  |  | 0.0047 | -0.0048 | 0.0846 |  |
| 7 | 1 year | sheep | the growing season | LG | 3 |  |  |  |  | | -1.3863 |  |  |  |  |  |  |  |
| 7 | 1 year | sheep | the growing season | LG | 3 |  |  |  |  | | -0.4055 |  |  |  |  |  |  |  |
| 7 | 1 year | sheep | the growing season | LG | 3 |  |  |  |  | | 0.2231 |  |  |  |  |  |  |  |
| 7 | 1 year | sheep | the growing season | LG | 3 |  |  |  |  | | 0.0953 |  |  |  |  |  |  |  |
| 7 | 1 year | sheep | the growing season | LG | 3 |  |  |  |  | | -0.2231 |  |  |  |  |  |  |  |
| 7 | 1 year | sheep | the growing season | LG | 3 |  |  |  |  | | -0.2231 |  |  |  |  |  |  |  |
| 7 | 1 year | sheep | the growing season | MG | 3 |  |  |  |  | | 0.3254 |  |  |  | 0.2879 | -0.0131 | -0.0299 |  |
| 7 | 1 year | sheep | the growing season | MG | 3 |  |  |  |  | | 0.3795 |  |  |  | 0.2006 | -0.0216 | -0.0198 |  |
| 7 | 1 year | sheep | the growing season | MG | 3 |  |  |  |  | | -0.539 |  |  |  |  |  |  |  |
| 7 | 1 year | sheep | the growing season | MG | 3 |  |  |  |  | | -0.4055 |  |  |  |  |  |  |  |
| 7 | 1 year | sheep | the growing season | MG | 3 |  |  |  |  | | -0.1054 |  |  |  |  |  |  |  |
| 7 | 1 year | sheep | the growing season | MG | 3 |  |  |  |  | | -0.3567 |  |  |  |  |  |  |  |
| 8 | ＞5 years | mixed | annual | EG | 3 | -1.7726 |  |  |  | | -0.1796 |  |  |  |  |  |  |  |
| 9 | ＞5 years | sheep | the growing season | LG | 3 | -0.3388 |  |  |  | |  |  |  |  |  |  |  |  |
| 9 | ＞5 years | sheep | the growing season | MG | 3 | -0.5947 |  |  |  | |  |  |  |  |  |  |  |  |
| 9 | ＞5 years | sheep | the growing season | HG | 3 | -0.9395 |  |  |  | |  |  |  |  |  |  |  |  |
| 19 | ＞5 years | sheep | annual | EG | 3 | -0.5419 |  |  |  | |  |  |  |  |  |  |  |  |
| 10 | ＞5 years | sheep | annual | EG | 3 | -1.6734 |  |  |  | |  |  |  |  |  |  |  |  |
| 10 | ＞5 years | sheep | annual | EG | 3 | -1.6545 |  |  |  | |  |  |  |  |  |  |  |  |
| 10 | ＞5 years | sheep | annual | EG | 3 | -1.2522 |  |  |  | |  |  |  |  |  |  |  |  |
| 10 | ＞5 years | sheep | annual | EG | 3 | -1.1819 |  |  |  | |  |  |  |  |  |  |  |  |
| 11 | ＞5 years | mixed | annual | HG | 3 | -2.986 |  |  |  | |  |  |  |  |  |  |  |  |
| 11 | ＞5 years | mixed | the growing season | LG | 3 | -0.3229 |  |  |  | |  |  |  |  |  |  |  |  |
| 11 | ＞5 years | mixed | the growing season | MG | 3 | -0.8156 |  |  |  | |  |  |  |  |  |  |  |  |
| 12 | ＞5 years | mixed | annual | EG | 4 | 0.05 |  |  |  | |  |  |  |  |  | -0.0144 | -0.012 | 0.3194 |
| 12 | ＞5 years | mixed | annual | EG | 4 | 0.1231 |  |  |  | |  |  |  |  |  | -0.0489 | 0 | -0.2231 |
| 12 | ＞5 years | mixed | non-growing season | LG | 4 | -0.2181 |  |  |  | |  |  |  |  |  | 0.0617 | 0.1843 | -0.2114 |
| 12 | ＞5 years | mixed | non-growing season | LG | 4 | 0.225 |  |  |  | |  |  |  |  |  | 0.0314 | 0.3228 | -0.3038 |
| 13 | ＞5 years | sheep | annual | MG | 3 | -0.8009 | -0.1146 | 0.6863 | -0.1806 | |  |  |  |  |  | 0.0348 | -0.2384 |  |
| 14 | 2-5 years | sheep | annual | MG | 3 |  |  |  |  | |  |  |  |  |  |  | 0.1759 | -0.377 |
| 15 | ＞5 years | sheep | annual | MG | 5 | -0.5961 | -0.196 | 0.4002 | -0.2771 | |  |  |  |  |  |  |  |  |
| 15 | ＞5 years | sheep | annual | MG | 5 | -0.5963 | -0.1474 | 0.4489 | -0.2348 | |  |  |  |  |  |  |  |  |
| 15 | ＞5 years | sheep | annual | MG | 5 | -0.5962 | -0.0148 | 0.5814 | -0.1381 | |  |  |  |  |  |  |  |  |
| 16 | 2-5 years | mixed | annual | EG | 5 |  |  |  |  | |  |  |  |  |  |  | 0.4263 |  |
| 16 | 2-5 years | mixed | annual | EG | 5 |  |  |  |  | |  |  |  |  |  |  | 0.0168 |  |
| 16 | 2-5 years | mixed | annual | EG | 5 |  |  |  |  | |  |  |  |  |  |  | 0.0255 |  |
| 16 | ＞5 years | mixed | annual | EG | 5 | -0.9409 | -0.1933 | 0.7476 | -0.2364 | | -0.1523 |  |  |  |  | 0.0142 |  |  |
| 16 | ＞5 years | mixed | annual | EG | 5 | -1.2823 | -0.3373 | 0.945 | -0.3779 | |  |  |  |  |  | 0.0278 |  |  |
| 16 | ＞5 years | mixed | annual | EG | 5 | -1.2455 | -0.2464 | 0.999 | -0.2904 | |  |  |  |  |  | 0.014 |  |  |
| 17 | ＞5 years | mixed | annual | LG | 3 | -0.0732 | -0.0163 | 0.1114 | 0.0295 | | 0.1211 | 0.069 |  |  |  | 0.0069 | 0.0157 | -0.0466 |
| 17 | ＞5 years | mixed | annual | LG | 3 |  | 0.1918 |  |  | | 0.3145 | 0 |  |  |  |  |  |  |
| 17 | ＞5 years | mixed | annual | LG | 3 |  | 0.081 |  |  | | 0.2318 | -0.0445 |  |  |  |  |  |  |
| 17 | ＞5 years | mixed | annual | LG | 3 |  | 0.1661 |  |  | | 0.0588 | 0.0513 |  |  |  |  |  |  |
| 17 | ＞5 years | mixed | annual | LG | 3 |  | 0.0382 |  |  | |  |  |  |  |  |  |  |  |
| 17 | ＞5 years | mixed | annual | MG | 3 | -0.3423 | 0.2543 | 0.5274 | 0.1505 | | 0.1724 | 0 |  |  |  | 0.0239 | 0.0389 | -0.3044 |
| 17 | ＞5 years | mixed | annual | MG | 3 |  | 0.1915 |  |  | | 0.4055 | -0.0426 |  |  |  |  |  |  |
| 17 | ＞5 years | mixed | annual | MG | 3 |  | -0.2975 |  |  | | 0.5534 | 0 |  |  |  |  |  |  |
| 17 | ＞5 years | mixed | annual | MG | 3 |  | -0.1208 |  |  | | 0.1144 | 0.0513 |  |  |  |  |  |  |
| 17 | ＞5 years | mixed | annual | MG | 3 |  | 0.1851 |  |  | |  |  |  |  |  |  |  |  |
| 17 | ＞5 years | mixed | annual | HG | 3 | -0.6481 | 0.0512 | 0.5277 | -0.1549 | | -0.3178 | -0.2877 |  |  |  | 0.0372 | 0.069 | -0.5318 |
| 17 | ＞5 years | mixed | annual | HG | 3 |  | -0.5661 |  |  | | -0.0674 | -0.2336 |  |  |  |  |  |  |
| 17 | ＞5 years | mixed | annual | HG | 3 |  | -0.8021 |  |  | | -0.1566 | -0.4274 |  |  |  |  |  |  |
| 17 | ＞5 years | mixed | annual | HG | 3 |  | -0.8382 |  |  | | -0.0465 | -0.2364 |  |  |  |  |  |  |
| 17 | ＞5 years | mixed | annual | HG | 3 |  | -0.1204 |  |  | |  |  |  |  |  |  |  |  |
| 18 | 1 year | mixed | the growing season | EG | 3 |  |  |  |  | | -0.2379 |  |  |  |  |  |  | 0.3847 |
| 18 | 1 year | mixed | the growing season | EG | 3 |  |  |  |  | | -0.4642 |  |  |  |  |  |  | -0.098 |
| 18 | 1 year | mixed | the growing season | EG | 3 |  |  |  |  | | -0.4064 |  |  |  |  |  |  | -0.0673 |
| 18 | 1 year | mixed | the growing season | EG | 3 |  |  |  |  | | -0.2462 |  |  |  |  |  |  | 0.0746 |
| 18 | 1 year | mixed | the growing season | MG | 3 |  |  |  |  | | -0.1105 |  |  |  |  |  |  | 0.0947 |
| 18 | 1 year | mixed | the growing season | MG | 3 |  |  |  |  | | -0.3 |  |  |  |  |  |  | 0.0488 |
| 18 | 1 year | mixed | the growing season | MG | 3 |  |  |  |  | | -0.2279 |  |  |  |  |  |  | 0.2066 |
| 18 | 1 year | mixed | the growing season | MG | 3 |  |  |  |  | | -0.2503 |  |  |  |  |  |  | 0.2288 |
| 18 | 2-5 years | mixed | annual | EG | 3 |  |  |  |  | | -0.0906 |  |  |  |  |  | -0.0796 | -0.8362 |
| 18 | 2-5 years | mixed | annual | EG | 3 |  |  |  |  | | -0.0885 |  |  |  |  |  | -0.0123 | -0.775 |
| 18 | 2-5 years | mixed | annual | EG | 3 |  |  |  |  | | -0.3718 |  |  |  |  |  | -0.0625 | -0.5151 |
| 18 | 2-5 years | mixed | annual | EG | 3 |  |  |  |  | | 0.0925 |  |  |  |  |  | -0.1268 | 1.1701 |
| 18 | 2-5 years | mixed | annual | EG | 3 |  |  |  |  | | 0.2962 |  |  |  |  |  |  | 1.1728 |
| 18 | 2-5 years | mixed | annual | EG | 3 |  |  |  |  | | 0.3928 |  |  |  |  |  |  | 1.2172 |
| 18 | 2-5 years | mixed | annual | EG | 3 |  |  |  |  | | 0.1466 |  |  |  |  |  |  | 0.3039 |
| 18 | 2-5 years | mixed | the growing season | MG | 3 |  |  |  |  | | 0.1827 |  |  |  |  |  | -0.1252 | 0.7565 |
| 18 | 2-5 years | mixed | the growing season | MG | 3 |  |  |  |  | | 1.0146 |  |  |  |  |  | -0.1163 | 0.9849 |
| 18 | 2-5 years | mixed | the growing season | MG | 3 |  |  |  |  | | 0.7151 |  |  |  |  |  | -0.186 | 0.9552 |
| 18 | 2-5 years | mixed | the growing season | MG | 3 |  |  |  |  | |  |  |  |  |  |  | -0.1268 | 0.5801 |
| 18 | 2-5 years | mixed | annual | MG | 3 |  |  |  |  | | -0.1328 |  |  |  |  |  | -0.1959 | -0.1736 |
| 18 | 2-5 years | mixed | annual | MG | 3 |  |  |  |  | | -0.1834 |  |  |  |  |  | -0.1493 | -0.0783 |
| 18 | 2-5 years | mixed | annual | MG | 3 |  |  |  |  | | -0.2227 |  |  |  |  |  | -0.159 | -0.0803 |
| 18 | 2-5 years | mixed | annual | MG | 3 |  |  |  |  | | -0.3954 |  |  |  |  |  | -0.1251 | 1.3239 |
| 18 | 2-5 years | mixed | annual | MG | 3 |  |  |  |  | | 0.6359 |  |  |  |  |  |  | 1.0357 |
| 18 | 2-5 years | mixed | annual | MG | 3 |  |  |  |  | | 1.2804 |  |  |  |  |  |  | 0.8972 |
| 18 | 2-5 years | mixed | annual | MG | 3 |  |  |  |  | | 1.3289 |  |  |  |  |  |  | 0.4929 |
| 18 | 2-5 years | cattle | non-growing season | LG | 3 |  |  |  |  | |  |  |  |  |  |  | -0.2085 | 1.5883 |
| 18 | 2-5 years | cattle | non-growing season | LG | 3 |  |  |  |  | |  |  |  |  |  |  | -0.2478 | 1.6422 |
| 18 | 2-5 years | cattle | non-growing season | LG | 3 |  |  |  |  | |  |  |  |  |  |  | -0.1715 | 1.1854 |
| 18 | 2-5 years | cattle | non-growing season | LG | 3 |  |  |  |  | |  |  |  |  |  |  | -0.2187 | 0.7153 |
| 19 | 1 year | sheep | the growing season | LG | 3 |  |  |  |  | | -0.2357 | -0.1542 |  |  | -0.4475 |  | -0.0074 | -0.0395 |
| 19 | 1 year | sheep | the growing season | LG | 3 |  |  |  |  | | -0.1365 | -0.1733 |  |  | -0.0049 |  | 0.0807 | -0.2231 |
| 19 | 1 year | sheep | the growing season | LG | 3 |  |  |  |  | | -0.0917 | -0.5773 |  |  | 0.0217 |  | 0.1247 | -0.3687 |
| 19 | 1 year | sheep | the growing season | LG | 3 |  |  |  |  | |  |  |  |  |  |  | 0.087 | 0.0765 |
| 19 | 1 year | sheep | the growing season | MG | 3 |  |  |  |  | | -0.1089 | -0.3365 |  |  | -0.4606 |  | 0.0361 | -0.1911 |
| 19 | 1 year | sheep | the growing season | MG | 3 |  |  |  |  | | -0.1178 | -0.3665 |  |  | -0.2467 |  | 0.0884 | -0.3102 |
| 19 | 1 year | sheep | the growing season | MG | 3 |  |  |  |  | | -0.3262 | -0.1512 |  |  | -0.618 |  | 0.0078 | -0.087 |
| 19 | 1 year | sheep | the growing season | MG | 3 |  |  |  |  | | -0.0362 | -0.3533 |  |  | -0.48 |  | -0.007 | 0.087 |
| 19 | 1 year | sheep | the growing season | MG | 3 |  |  |  |  | | -0.1271 | -0.3342 |  |  | -0.2182 |  | 0.0361 | -0.2201 |
| 19 | 1 year | sheep | the growing season | MG | 3 |  |  |  |  | | -0.3775 | 0.3264 |  |  | -0.9245 |  | 0.1554 | -0.4359 |
| 19 | 1 year | sheep | the growing season | MG | 3 |  |  |  |  | |  |  |  |  |  |  | 0.2356 | -0.418 |
| 19 | 1 year | sheep | the growing season | MG | 3 |  |  |  |  | |  |  |  |  |  |  | 0.0676 | -0.0347 |
| 19 | 1 year | sheep | the growing season | HG | 3 |  |  |  |  | | -0.0399 | -0.7172 |  |  | -0.5946 |  | 0.0572 | -0.2415 |
| 19 | 1 year | sheep | the growing season | HG | 3 |  |  |  |  | | -0.0949 | -0.3665 |  |  | -0.3845 |  | 0.1112 | -0.4055 |
| 19 | 1 year | sheep | the growing season | HG | 3 |  |  |  |  | | -0.312 | 0.0841 |  |  | -1.1278 |  | 0.2719 | -0.1823 |
| 19 | 1 year | sheep | the growing season | HG | 3 |  |  |  |  | | -0.0071 | -0.8199 |  |  | -0.6624 |  | 0.1962 | -0.0585 |
| 19 | 1 year | sheep | the growing season | HG | 3 |  |  |  |  | | 0.0364 | -0.8938 |  |  | -0.4393 |  | 0.0913 | -0.2634 |
| 19 | 1 year | sheep | the growing season | HG | 3 |  |  |  |  | | -0.1559 | 0.1313 |  |  | -0.7461 |  | 0.1837 | -0.4055 |
| 19 | 1 year | sheep | the growing season | HG | 3 |  |  |  |  | |  |  |  |  |  |  | 0.1652 | 0.08 |
| 19 | 1 year | sheep | the growing season | HG | 3 |  |  |  |  | |  |  |  |  |  |  | 0.007 | 0.2231 |
| 20 | 1 year | sheep | the growing season | LG | 3 | -0.1426 | -0.0666 | 0.076 | -0.0695 | |  |  |  |  |  |  |  |  |
| 20 | 1 year | sheep | the growing season | MG | 3 | -0.0013 | -0.1277 | -0.1265 | -0.1226 | |  |  |  |  |  |  |  |  |
| 20 | 1 year | sheep | the growing season | MG | 3 | -0.0756 | -0.1439 | -0.0683 | -0.1411 | |  |  |  |  |  |  |  |  |
| 20 | 1 year | sheep | the growing season | HG | 3 | -0.3987 | -0.2342 | 0.1645 | -0.2401 | |  |  |  |  |  |  |  |  |
| 20 | 1 year | sheep | the growing season | HG | 3 | -0.4937 | -0.335 | 0.1586 | -0.3407 | |  |  |  |  |  |  |  |  |
| 21 | 1 year | sheep | the growing season | LG | 3 | 0.027 |  |  |  | | -0.2013 | 0.5819 |  |  | 0.1133 |  |  | -0.0352 |
| 21 | 1 year | sheep | the growing season | LG | 3 | 0.027 |  |  |  | | -0.2219 | 0 |  |  | 0.0577 |  |  | -0.0228 |
| 21 | 1 year | sheep | the growing season | LG | 3 | 0.027 |  |  |  | |  |  |  |  |  |  |  | 0.0042 |
| 21 | 1 year | sheep | the growing season | MG | 3 | -0.2347 |  |  |  | | -0.1119 | 0.7191 |  |  | 0.1117 |  |  | -0.0817 |
| 21 | 1 year | sheep | the growing season | MG | 3 | -0.2347 |  |  |  | | -0.202 | -0.2151 |  |  | -0.0853 |  |  | -0.057 |
| 21 | 1 year | sheep | the growing season | MG | 3 | -0.2347 |  |  |  | |  |  |  |  |  |  |  | 0.0256 |
| 21 | 1 year | sheep | the growing season | HG | 3 | -0.4165 |  |  |  | | -0.0752 | 0.1466 |  |  | 0.0752 |  |  | -0.0961 |
| 21 | 1 year | sheep | the growing season | HG | 3 | -0.4165 |  |  |  | | -0.1199 | -0.3895 |  |  | -0.1528 |  |  | -0.0789 |
| 21 | 1 year | sheep | the growing season | HG | 3 | -0.4165 |  |  |  | |  |  |  |  |  |  |  | 0.0418 |
| 22 | ＞5 years | mixed | annual | LG | 3 | -0.1188 |  |  |  | | 0.147 |  |  |  |  |  |  |  |
| 22 | ＞5 years | mixed | annual | LG | 3 |  |  |  |  | | 0.0674 |  |  |  |  |  |  |  |
| 22 | ＞5 years | mixed | annual | LG | 3 |  |  |  |  | | 0.042 |  |  |  |  |  |  |  |
| 22 | ＞5 years | mixed | annual | LG | 3 |  |  |  |  | | 0.0889 |  |  |  |  |  |  |  |
| 22 | ＞5 years | mixed | annual | LG | 3 |  |  |  |  | | 0.0953 |  |  |  |  |  |  |  |
| 22 | ＞5 years | mixed | annual | MG | 3 | -0.3544 |  |  |  | | 0.1542 |  |  |  |  |  |  |  |
| 22 | ＞5 years | mixed | annual | MG | 3 |  |  |  |  | | 0.0783 |  |  |  |  |  |  |  |
| 22 | ＞5 years | mixed | annual | MG | 3 |  |  |  |  | | 0.069 |  |  |  |  |  |  |  |
| 22 | ＞5 years | mixed | annual | MG | 3 |  |  |  |  | | 0.1706 |  |  |  |  |  |  |  |
| 22 | ＞5 years | mixed | annual | MG | 3 |  |  |  |  | | 0.1178 |  |  |  |  |  |  |  |
| 22 | ＞5 years | mixed | annual | HG | 3 | -0.933 |  |  |  | | -0.0426 |  |  |  |  |  |  |  |
| 22 | ＞5 years | mixed | annual | HG | 3 |  |  |  |  | | 0.0455 |  |  |  |  |  |  |  |
| 22 | ＞5 years | mixed | annual | HG | 3 |  |  |  |  | | 0 |  |  |  |  |  |  |  |
| 22 | ＞5 years | mixed | annual | HG | 3 |  |  |  |  | | 0.0674 |  |  |  |  |  |  |  |
| 22 | ＞5 years | mixed | annual | HG | 3 |  |  |  |  | | 0 |  |  |  |  |  |  |  |
| 23 | 1 year | cattle | the growing season | LG | 3 | -0.2885 | -0.2041 | 0.0844 | -0.2558 | | -0.149 | 0.0619 | -0.1198 | -0.1625 |  | 0.0286 | 0.0213 | -0.1043 |
| 23 | 1 year | cattle | the growing season | LG | 3 |  |  |  |  | | -0.2348 | 0.0522 | -0.2403 | -0.0299 |  |  |  |  |
| 23 | 1 year | cattle | the growing season | LG | 3 |  |  |  |  | | -0.2556 | 0.1603 | -0.0859 | -0.1612 |  |  |  |  |
| 23 | 1 year | cattle | the growing season | LG | 3 |  |  |  |  | | 0.1178 | -0.1027 | -0.0215 | -0.1981 |  |  |  |  |
| 23 | 1 year | cattle | the growing season | MG | 3 | -0.3665 | -0.2942 | 0.0723 | -0.3386 | | -0.0051 | -0.066 | -0.0829 | 0.0466 |  | 0.042 | 0.0447 | -0.1728 |
| 23 | 1 year | cattle | the growing season | MG | 3 |  |  |  |  | | 0.041 | -0.018 | -0.0949 | -0.0652 |  |  |  |  |
| 23 | 1 year | cattle | the growing season | MG | 3 |  |  |  |  | | -0.0479 | -0.091 | -0.1307 | -0.2371 |  |  |  |  |
| 23 | 1 year | cattle | the growing season | MG | 3 |  |  |  |  | | -0.0074 | -0.1582 | -0.0084 | -0.1981 |  |  |  |  |
| 23 | 1 year | cattle | the growing season | HG | 3 | -0.427 | -0.4766 | -0.0495 | -0.4454 | | -0.026 | -0.1866 | -0.1236 | 0.0045 |  | 0.0624 | 0.0826 | -0.2706 |
| 23 | 1 year | cattle | the growing season | HG | 3 |  |  |  |  | | -0.0874 | -0.0935 | -0.1434 | -0.1769 |  |  |  |  |
| 23 | 1 year | cattle | the growing season | HG | 3 |  |  |  |  | | -0.1173 | -0.2177 | -0.2159 | -0.1638 |  |  |  |  |
| 23 | 1 year | cattle | the growing season | HG | 3 |  |  |  |  | | 0.1993 | -0.3814 |  | -0.1981 |  |  |  |  |
| 24 | 2-5 years | sheep | annual | EG | 3 | -1.0025 |  |  |  | |  |  |  |  |  |  |  | -0.163 |
| 24 | 2-5 years | sheep | non-growing season | MG | 3 | -0.6148 |  |  |  | |  |  |  |  |  |  |  | -0.0598 |
| 25 | 1 year | sheep | the growing season | LG | 3 | -0.1634 |  |  |  | |  |  |  |  |  |  | 0.014 | -0.0417 |
| 25 | 1 year | sheep | the growing season | LG | 3 |  |  |  |  | |  |  |  |  |  |  | 0.0441 | -0.0827 |
| 25 | 1 year | sheep | the growing season | LG | 3 |  |  |  |  | |  |  |  |  |  |  | 0.0374 | -0.0306 |
| 25 | 1 year | sheep | the growing season | MG | 3 | -0.8993 |  |  |  | |  |  |  |  |  |  | 0.039 | -0.0469 |
| 25 | 1 year | sheep | the growing season | MG | 3 |  |  |  |  | |  |  |  |  |  |  | 0.0464 | -0.2792 |
| 25 | 1 year | sheep | the growing season | MG | 3 |  |  |  |  | |  |  |  |  |  |  | 0.0413 | 0.2844 |
| 25 | 1 year | sheep | the growing season | HG | 3 | -1.9949 |  |  |  | |  |  |  |  |  |  | 0.0603 | -0.1115 |
| 25 | 1 year | sheep | the growing season | HG | 3 |  |  |  |  | |  |  |  |  |  |  | 0.0558 | -0.4773 |
| 25 | 1 year | sheep | the growing season | HG | 3 |  |  |  |  | |  |  |  |  |  |  | 0.0475 | 0.3267 |
| 26 | 2-5 years | cattle | the growing season | LG | 3 | -0.5195 | 0.0502 | 0.5697 | 0.0355 | | -0.1025 | -0.0753 | -0.1143 | -0.0794 | -0.3157 | 0.0043 | -0.0944 | -0.0128 |
| 26 | 2-5 years | cattle | the growing season | LG | 3 | -0.702 | 0.1812 | 0.8832 | 0.1613 | | -0.0253 | -0.0157 | 0.0166 | -0.072 | -0.1927 | -0.0037 | -0.0509 | -0.0257 |
| 26 | 2-5 years | cattle | the growing season | LG | 3 |  |  |  |  | | 0.037 | 0.0548 | -0.1248 | -0.0336 | -0.2763 | -0.0083 | -0.1345 | 0.1302 |
| 26 | 2-5 years | cattle | the growing season | LG | 3 |  |  |  |  | | -0.0783 | -0.0051 | -0.0856 | -0.022 | -0.1286 | 0.0005 | 0.0138 | -0.0127 |
| 26 | 2-5 years | cattle | the growing season | LG | 3 |  |  |  |  | | -0.1184 | 0.142 | -0.0674 | 0.0382 | -0.1234 | -0.0235 | -0.0523 | -0.0592 |
| 26 | 2-5 years | cattle | the growing season | LG | 3 |  |  |  |  | | -0.0924 | 0.092 | 0.0073 | 0.297 | -0.2555 | -0.0137 | 0.164 | -0.04 |
| 26 | 2-5 years | cattle | the growing season | HG | 3 | -0.4481 | -0.156 | 0.2921 | -0.1645 | | -0.4628 | -0.052 | -0.1387 | -0.0068 | -0.3559 | -0.0151 | -0.0959 | 0.0516 |
| 26 | 2-5 years | cattle | the growing season | HG | 3 | -0.9844 | 0.1252 | 1.1096 | 0.1025 | | -0.1982 | -0.1256 | -0.3358 | 0.0721 | -0.4033 | -0.0178 | -0.2031 | -0.197 |
| 26 | 2-5 years | cattle | the growing season | HG | 3 |  |  |  |  | | -0.4022 | 0.03 | 0 | -0.0858 | -0.3041 | 0.0041 | -0.051 | -0.1948 |
| 26 | 2-5 years | cattle | the growing season | HG | 3 |  |  |  |  | | -0.4292 | -0.1164 | -0.1734 | 0.0426 | -0.2534 | -0.0036 | 0.0523 | -0.0736 |
| 26 | 2-5 years | cattle | the growing season | HG | 3 |  |  |  |  | | 0.0587 | 0.092 | -0.0655 | -0.0969 | -0.2041 | -0.0245 | -0.0102 | -0.1237 |
| 26 | 2-5 years | cattle | the growing season | HG | 3 |  |  |  |  | | -0.515 | 0.0869 | -0.1503 | 0.1875 | -0.3455 | -0.0101 | 0.004 | -0.1461 |
| 27 | 2-5 years | sheep | the growing season | LG | 3 |  |  |  |  | |  |  |  |  |  |  | 0.0144 |  |
| 27 | 2-5 years | sheep | the growing season | LG | 3 |  |  |  |  | |  |  |  |  |  |  | 0.1214 |  |
| 27 | 2-5 years | sheep | the growing season | MG | 3 |  |  |  |  | |  |  |  |  |  |  | 0.0286 |  |
| 27 | 2-5 years | sheep | the growing season | MG | 3 |  |  |  |  | |  |  |  |  |  |  | 0.1355 |  |
| 27 | 2-5 years | sheep | the growing season | HG | 3 |  |  |  |  | |  |  |  |  |  |  | 0.0215 |  |
| 27 | 2-5 years | sheep | the growing season | HG | 3 |  |  |  |  | |  |  |  |  |  |  | 0.1285 |  |
| 27 | 2-5 years | sheep | the growing season | EG | 3 |  |  |  |  | |  |  |  |  |  |  | 0 |  |
| 27 | 2-5 years | sheep | the growing season | EG | 3 |  |  |  |  | |  |  |  |  |  |  | 0.107 |  |
| 27 | ＞5 years | sheep | the growing season | LG | 3 |  |  |  |  | | -0.0361 | 0 | -0.257 |  | -0.0942 | -0.0104 |  |  |
| 27 | ＞5 years | sheep | the growing season | LG | 3 |  |  |  |  | | -0.1933 | -0.0606 | -0.5238 |  | -0.1879 | 0.0039 |  |  |
| 27 | ＞5 years | sheep | the growing season | MG | 3 |  |  |  |  | | -0.0288 | 0 | -0.2238 |  | -0.065 | 0.0065 |  |  |
| 27 | ＞5 years | sheep | the growing season | MG | 3 |  |  |  |  | | -0.186 | -0.0606 | -0.4905 |  | -0.1587 | 0.0208 |  |  |
| 27 | ＞5 years | sheep | the growing season | HG | 3 |  |  |  |  | | -0.0288 | 0.0606 | -0.4032 |  | -0.0957 | 0.0129 |  |  |
| 27 | ＞5 years | sheep | the growing season | HG | 3 |  |  |  |  | | -0.186 | 0 | -0.67 |  | -0.1894 | 0.0272 |  |  |
| 27 | ＞5 years | sheep | the growing season | EG | 3 |  |  |  |  | | 0 | -0.0984 | 0.0003 |  | -0.0591 | -0.0052 |  |  |
| 27 | ＞5 years | sheep | the growing season | EG | 3 |  |  |  |  | | -0.1572 | -0.1591 | -0.2664 |  | -0.1528 | 0.0092 |  |  |
| 28 | ＞5 years | mixed | annual | LG | 3 | -0.3694 |  |  |  | |  |  |  |  |  |  |  |  |
| 28 | ＞5 years | mixed | annual | MG | 3 | -0.8088 |  |  |  | |  |  |  |  |  |  |  |  |
| 28 | ＞5 years | mixed | annual | HG | 3 | -1.313 |  |  |  | |  |  |  |  |  |  |  |  |
| 29 | 2-5 years | sheep | the growing season | MG | 3 |  |  |  |  | |  |  |  |  |  |  | 0.0715 |  |
| 29 | 2-5 years | sheep | the growing season | MG | 3 |  |  |  |  | |  |  |  |  |  |  | 0.0294 |  |
| 29 | 2-5 years | sheep | the growing season | MG | 3 |  |  |  |  | |  |  |  |  |  |  | 0.0072 |  |
| 29 | 2-5 years | sheep | the growing season | MG | 3 |  |  |  |  | |  |  |  |  |  |  | -0.0073 |  |
| 29 | 2-5 years | sheep | the growing season | HG | 3 |  |  |  |  | |  |  |  |  |  |  | 0.1054 |  |
| 29 | 2-5 years | sheep | the growing season | HG | 3 |  |  |  |  | |  |  |  |  |  |  | 0.0858 |  |
| 29 | 2-5 years | sheep | the growing season | HG | 3 |  |  |  |  | |  |  |  |  |  |  | 0.0286 |  |
| 29 | 2-5 years | sheep | the growing season | HG | 3 |  |  |  |  | |  |  |  |  |  |  | 0.0144 |  |
| 29 | ＞5 years | sheep | the growing season | MG | 3 |  |  |  |  | | -0.0741 | 0.1703 | 0.1507 | -0.0118 |  |  |  | -0.1948 |
| 29 | ＞5 years | sheep | the growing season | MG | 3 |  |  |  |  | | 0 | -0.1957 | 0.0734 | -0.2788 |  |  |  | -0.1114 |
| 29 | ＞5 years | sheep | the growing season | MG | 3 |  |  |  |  | | 0.087 | 0.08 | -0.112 | 0.5501 |  |  |  | -0.1368 |
| 29 | ＞5 years | sheep | the growing season | MG | 3 |  |  |  |  | | -0.0741 | -0.3429 | 0.1583 | -0.2684 |  |  |  | -0.3165 |
| 29 | ＞5 years | sheep | the growing season | HG | 3 |  |  |  |  | | 0.069 | -0.5108 | 0.2178 | -0.155 |  |  |  | -0.3912 |
| 29 | ＞5 years | sheep | the growing season | HG | 3 |  |  |  |  | | 0.1542 | -0.2231 | -0.0164 | 0.2154 |  |  |  | -0.5071 |
| 29 | ＞5 years | sheep | the growing season | HG | 3 |  |  |  |  | | 0.087 | 0.3285 | -0.0811 | 0.9675 |  |  |  | -0.2826 |
| 29 | ＞5 years | sheep | the growing season | HG | 3 |  |  |  |  | | 0.1335 | -0.1759 | 0.2597 | 0.1116 |  |  |  | -0.0204 |
| 30 | 2-5 years | cattle | annual | LG | 3 |  |  |  |  | |  |  |  |  |  |  | 0.0839 |  |
| 30 | 2-5 years | cattle | annual | LG | 3 |  |  |  |  | |  |  |  |  |  |  | -0.2134 |  |
| 30 | 2-5 years | cattle | annual | LG | 3 |  |  |  |  | |  |  |  |  |  |  | -0.285 |  |
| 30 | 2-5 years | cattle | annual | LG | 3 |  |  |  |  | |  |  |  |  |  |  | 0.3018 |  |
| 30 | 2-5 years | cattle | annual | LG | 3 |  |  |  |  | |  |  |  |  |  |  | -0.2086 |  |
| 30 | 2-5 years | cattle | annual | LG | 3 |  |  |  |  | |  |  |  |  |  |  | -0.007 |  |
| 30 | 2-5 years | cattle | annual | LG | 3 |  |  |  |  | |  |  |  |  |  |  | -0.0381 |  |
| 30 | 2-5 years | cattle | annual | LG | 3 |  |  |  |  | |  |  |  |  |  |  | -0.1577 |  |
| 30 | 2-5 years | cattle | annual | LG | 3 |  |  |  |  | |  |  |  |  |  |  | 0.2145 |  |
| 30 | 2-5 years | cattle | annual | MG | 3 |  |  |  |  | |  |  |  |  |  |  | 0.1284 |  |
| 30 | 2-5 years | cattle | annual | MG | 3 |  |  |  |  | |  |  |  |  |  |  | 0.0775 |  |
| 30 | 2-5 years | cattle | annual | MG | 3 |  |  |  |  | |  |  |  |  |  |  | 0.1466 |  |
| 30 | 2-5 years | cattle | annual | MG | 3 |  |  |  |  | |  |  |  |  |  |  | -0.0117 |  |
| 30 | 2-5 years | cattle | annual | MG | 3 |  |  |  |  | |  |  |  |  |  |  | 0.0391 |  |
| 30 | 2-5 years | cattle | annual | MG | 3 |  |  |  |  | |  |  |  |  |  |  | -0.0459 |  |
| 30 | 2-5 years | cattle | annual | MG | 3 |  |  |  |  | |  |  |  |  |  |  | 0.6703 |  |
| 30 | 2-5 years | cattle | annual | MG | 3 |  |  |  |  | |  |  |  |  |  |  | -0.6063 |  |
| 30 | 2-5 years | cattle | annual | MG | 3 |  |  |  |  | |  |  |  |  |  |  | -0.4261 |  |
| 30 | 2-5 years | cattle | annual | HG | 3 |  |  |  |  | |  |  |  |  |  |  | 0.0597 |  |
| 30 | 2-5 years | cattle | annual | HG | 3 |  |  |  |  | |  |  |  |  |  |  | -0.0909 |  |
| 30 | 2-5 years | cattle | annual | HG | 3 |  |  |  |  | |  |  |  |  |  |  | -0.0546 |  |
| 30 | 2-5 years | cattle | annual | HG | 3 |  |  |  |  | |  |  |  |  |  |  | 0.1189 |  |
| 30 | 2-5 years | cattle | annual | HG | 3 |  |  |  |  | |  |  |  |  |  |  | 0.077 |  |
| 30 | 2-5 years | cattle | annual | HG | 3 |  |  |  |  | |  |  |  |  |  |  | 0.0153 |  |
| 30 | 2-5 years | cattle | annual | HG | 3 |  |  |  |  | |  |  |  |  |  |  | -0.12 |  |
| 30 | 2-5 years | cattle | annual | HG | 3 |  |  |  |  | |  |  |  |  |  |  | -0.775 |  |
| 30 | 2-5 years | cattle | annual | HG | 3 |  |  |  |  | |  |  |  |  |  |  | -0.4135 |  |
| 30 | ＞5 years | cattle | annual | LG | 3 |  | -0.3597 |  |  | |  |  |  |  |  |  |  | -0.017 |
| 30 | ＞5 years | cattle | annual | LG | 3 |  | 0.211 |  |  | |  |  |  |  |  |  |  | 0.0148 |
| 30 | ＞5 years | cattle | annual | LG | 3 |  | -0.0772 |  |  | |  |  |  |  |  |  |  | 0.1997 |
| 30 | ＞5 years | cattle | annual | LG | 3 |  | -0.1569 |  |  | |  |  |  |  |  |  |  | -0.1134 |
| 30 | ＞5 years | cattle | annual | LG | 3 |  | -0.2042 |  |  | |  |  |  |  |  |  |  | 0.292 |
| 30 | ＞5 years | cattle | annual | LG | 3 |  | -0.2147 |  |  | |  |  |  |  |  |  |  | -0.203 |
| 30 | ＞5 years | cattle | annual | LG | 3 |  | -0.057 |  |  | |  |  |  |  |  |  |  | 0.0462 |
| 30 | ＞5 years | cattle | annual | LG | 3 |  | 0.0188 |  |  | |  |  |  |  |  |  |  | 0.0818 |
| 30 | ＞5 years | cattle | annual | LG | 3 |  | 0.208 |  |  | |  |  |  |  |  |  |  | 0.489 |
| 30 | ＞5 years | cattle | annual | LG | 3 |  | -0.057 |  |  | |  |  |  |  |  |  |  |  |
| 30 | ＞5 years | cattle | annual | LG | 3 |  | 0.0188 |  |  | |  |  |  |  |  |  |  |  |
| 30 | ＞5 years | cattle | annual | LG | 3 |  | 0.208 |  |  | |  |  |  |  |  |  |  |  |
| 30 | ＞5 years | cattle | annual | LG | 3 |  | -0.1144 |  |  | |  |  |  |  |  |  |  |  |
| 30 | ＞5 years | cattle | annual | LG | 3 |  | -0.0409 |  |  | |  |  |  |  |  |  |  |  |
| 30 | ＞5 years | cattle | annual | LG | 3 |  | 0.2623 |  |  | |  |  |  |  |  |  |  |  |
| 30 | ＞5 years | cattle | annual | LG | 3 |  | -0.1144 |  |  | |  |  |  |  |  |  |  |  |
| 30 | ＞5 years | cattle | annual | LG | 3 |  | -0.0409 |  |  | |  |  |  |  |  |  |  |  |
| 30 | ＞5 years | cattle | annual | LG | 3 |  | 0.2623 |  |  | |  |  |  |  |  |  |  |  |
| 30 | ＞5 years | cattle | annual | MG | 3 |  | 0.1054 |  |  | |  |  |  |  |  |  |  | -0.0505 |
| 30 | ＞5 years | cattle | annual | MG | 3 |  | 0.0299 |  |  | |  |  |  |  |  |  |  | -0.0309 |
| 30 | ＞5 years | cattle | annual | MG | 3 |  | 0.2297 |  |  | |  |  |  |  |  |  |  | -0.1083 |
| 30 | ＞5 years | cattle | annual | MG | 3 |  | -0.2177 |  |  | |  |  |  |  |  |  |  | 0.0922 |
| 30 | ＞5 years | cattle | annual | MG | 3 |  | -0.042 |  |  | |  |  |  |  |  |  |  | -0.1559 |
| 30 | ＞5 years | cattle | annual | MG | 3 |  | -0.1216 |  |  | |  |  |  |  |  |  |  | 0.009 |
| 30 | ＞5 years | cattle | annual | MG | 3 |  | -0.017 |  |  | |  |  |  |  |  |  |  | -0.0998 |
| 30 | ＞5 years | cattle | annual | MG | 3 |  | 0.5386 |  |  | |  |  |  |  |  |  |  | -0.0681 |
| 30 | ＞5 years | cattle | annual | MG | 3 |  | -0.2606 |  |  | |  |  |  |  |  |  |  | 0.1184 |
| 30 | ＞5 years | cattle | annual | MG | 3 |  | 0.0183 |  |  | |  |  |  |  |  |  |  |  |
| 30 | ＞5 years | cattle | annual | MG | 3 |  | -0.2417 |  |  | |  |  |  |  |  |  |  |  |
| 30 | ＞5 years | cattle | annual | MG | 3 |  | -0.1474 |  |  | |  |  |  |  |  |  |  |  |
| 30 | ＞5 years | cattle | annual | MG | 3 |  | -0.3793 |  |  | |  |  |  |  |  |  |  |  |
| 30 | ＞5 years | cattle | annual | MG | 3 |  | -0.1235 |  |  | |  |  |  |  |  |  |  |  |
| 30 | ＞5 years | cattle | annual | MG | 3 |  | -0.1998 |  |  | |  |  |  |  |  |  |  |  |
| 30 | ＞5 years | cattle | annual | MG | 3 |  | 0.0128 |  |  | |  |  |  |  |  |  |  |  |
| 30 | ＞5 years | cattle | annual | MG | 3 |  | -0.3082 |  |  | |  |  |  |  |  |  |  |  |
| 30 | ＞5 years | cattle | annual | MG | 3 |  | -0.164 |  |  | |  |  |  |  |  |  |  |  |
| 30 | ＞5 years | cattle | annual | HG | 3 |  | 0.3546 |  |  | |  |  |  |  |  |  |  | -0.1088 |
| 30 | ＞5 years | cattle | annual | HG | 3 |  | -0.027 |  |  | |  |  |  |  |  |  |  | -0.058 |
| 30 | ＞5 years | cattle | annual | HG | 3 |  | 0.3737 |  |  | |  |  |  |  |  |  |  | -0.033 |
| 30 | ＞5 years | cattle | annual | HG | 3 |  | 0.1657 |  |  | |  |  |  |  |  |  |  | -0.0905 |
| 30 | ＞5 years | cattle | annual | HG | 3 |  | 0.356 |  |  | |  |  |  |  |  |  |  | -0.1353 |
| 30 | ＞5 years | cattle | annual | HG | 3 |  | 0.0108 |  |  | |  |  |  |  |  |  |  | -0.331 |
| 30 | ＞5 years | cattle | annual | HG | 3 |  | -0.0681 |  |  | |  |  |  |  |  |  |  | 0.0777 |
| 30 | ＞5 years | cattle | annual | HG | 3 |  | -0.0637 |  |  | |  |  |  |  |  |  |  | 0.0588 |
| 30 | ＞5 years | cattle | annual | HG | 3 |  | 0.2014 |  |  | |  |  |  |  |  |  |  | 0.1043 |
| 30 | ＞5 years | cattle | annual | HG | 3 |  | 0.1477 |  |  | |  |  |  |  |  |  |  |  |
| 30 | ＞5 years | cattle | annual | HG | 3 |  | 0.1267 |  |  | |  |  |  |  |  |  |  |  |
| 30 | ＞5 years | cattle | annual | HG | 3 |  | 0.3596 |  |  | |  |  |  |  |  |  |  |  |
| 30 | ＞5 years | cattle | annual | HG | 3 |  | -0.0088 |  |  | |  |  |  |  |  |  |  |  |
| 30 | ＞5 years | cattle | annual | HG | 3 |  | -0.5115 |  |  | |  |  |  |  |  |  |  |  |
| 30 | ＞5 years | cattle | annual | HG | 3 |  | 0.0139 |  |  | |  |  |  |  |  |  |  |  |
| 30 | ＞5 years | cattle | annual | HG | 3 |  | 0.1296 |  |  | |  |  |  |  |  |  |  |  |
| 30 | ＞5 years | cattle | annual | HG | 3 |  | 0.167 |  |  | |  |  |  |  |  |  |  |  |
| 30 | ＞5 years | cattle | annual | HG | 3 |  | 0.1761 |  |  | |  |  |  |  |  |  |  |  |
| 30 | ＞5 years | cattle | the growing season | LG | 3 | -0.2812 | -0.2788 | 0.0024 | -0.2796 | |  |  |  |  |  |  |  |  |
| 30 | ＞5 years | cattle | the growing season | LG | 3 | -0.8248 | -0.1689 | 0.6559 | -0.2676 | |  |  |  |  |  |  |  |  |
| 30 | ＞5 years | cattle | the growing season | LG | 3 | 0.0787 | -0.0302 | -0.1089 | -0.0007 | |  |  |  |  |  |  |  |  |
| 30 | ＞5 years | cattle | the growing season | LG | 3 | -0.2269 | -0.0302 | 0.1968 | -0.0561 | |  |  |  |  |  |  |  |  |
| 30 | ＞5 years | cattle | the growing season | LG | 3 | -0.0363 | -0.0625 | -0.0262 | -0.0579 | |  |  |  |  |  |  |  |  |
| 30 | ＞5 years | cattle | the growing season | LG | 3 | -0.23 | -0.0625 | 0.1675 | -0.0881 | |  |  |  |  |  |  |  |  |
| 30 | ＞5 years | cattle | the growing season | MG | 3 | -0.1372 | 0.105 | 0.2422 | 0.0329 | |  |  |  |  |  |  |  |  |
| 30 | ＞5 years | cattle | the growing season | MG | 3 | -0.7335 | -0.1728 | 0.5607 | -0.2391 | |  |  |  |  |  |  |  |  |
| 30 | ＞5 years | cattle | the growing season | MG | 3 | -0.0738 | 0.0342 | 0.108 | 0.0174 | |  |  |  |  |  |  |  |  |
| 30 | ＞5 years | cattle | the growing season | MG | 3 | -0.2802 | -0.0358 | 0.2444 | -0.061 | |  |  |  |  |  |  |  |  |
| 30 | ＞5 years | cattle | the growing season | MG | 3 | -0.1192 | -0.3257 | -0.2066 | -0.3077 | |  |  |  |  |  |  |  |  |
| 30 | ＞5 years | cattle | the growing season | MG | 3 | 0.0198 | -0.0548 | -0.0746 | -0.0442 | |  |  |  |  |  |  |  |  |
| 30 | ＞5 years | cattle | the growing season | HG | 3 | -0.1949 | 0.2985 | 0.4934 | 0.1566 | |  |  |  |  |  |  |  |  |
| 30 | ＞5 years | cattle | the growing season | HG | 3 | -1.1502 | 0.1853 | 1.3355 | 0.0159 | |  |  |  |  |  |  |  |  |
| 30 | ＞5 years | cattle | the growing season | HG | 3 | -0.4498 | -0.0458 | 0.404 | -0.1172 | |  |  |  |  |  |  |  |  |
| 30 | ＞5 years | cattle | the growing season | HG | 3 | -0.4126 | 0.1623 | 0.5749 | 0.1162 | |  |  |  |  |  |  |  |  |
| 30 | ＞5 years | cattle | the growing season | HG | 3 | -0.1886 | -0.106 | 0.0826 | -0.115 | |  |  |  |  |  |  |  |  |
| 30 | ＞5 years | cattle | the growing season | HG | 3 | -0.2211 | 0.1404 | 0.3615 | 0.0889 | |  |  |  |  |  |  |  |  |
| 31 | ＞5 years | mixed | annual | LG | 3 | -0.1188 |  |  |  | |  |  |  |  |  |  |  |  |
| 31 | ＞5 years | mixed | annual | MG | 3 | -0.3544 |  |  |  | |  |  |  |  |  |  |  |  |
| 31 | ＞5 years | mixed | annual | HG | 3 | -0.933 |  |  |  | |  |  |  |  |  |  |  |  |
| 32 | 2-5 years | sheep | the growing season | LG | 3 |  |  |  |  | |  |  |  |  |  |  | 0.0072 | -0.0418 |
| 32 | 2-5 years | sheep | the growing season | LG | 3 |  |  |  |  | |  |  |  |  |  |  | 0.0353 | -0.2773 |
| 32 | 2-5 years | sheep | the growing season | LG | 3 |  |  |  |  | |  |  |  |  |  |  | 0 | -0.0094 |
| 32 | 2-5 years | sheep | the growing season | MG | 3 |  |  |  |  | |  |  |  |  |  |  | 0.0426 | -0.1384 |
| 32 | 2-5 years | sheep | the growing season | MG | 3 |  |  |  |  | |  |  |  |  |  |  | 0.109 | -0.252 |
| 32 | 2-5 years | sheep | the growing season | MG | 3 |  |  |  |  | |  |  |  |  |  |  | 0.0741 | 0.0007 |
| 32 | 2-5 years | sheep | the growing season | HG | 3 |  |  |  |  | |  |  |  |  |  |  | 0.07 | -0.4228 |
| 32 | 2-5 years | sheep | the growing season | HG | 3 |  |  |  |  | |  |  |  |  |  |  | 0.0695 | -0.3003 |
| 32 | 2-5 years | sheep | the growing season | HG | 3 |  |  |  |  | |  |  |  |  |  |  | 0.0478 | -0.1079 |
| 33 | 1 year | sheep | the growing season | MG | 3 |  | -0.439 |  |  | |  |  |  |  |  |  |  |  |
| 33 | 1 year | sheep | the growing season | MG | 3 |  | 0.5662 |  |  | |  |  |  |  |  |  |  |  |
| 33 | 1 year | sheep | the growing season | MG | 3 |  | 0.1147 |  |  | |  |  |  |  |  |  |  |  |
| 33 | 1 year | sheep | the growing season | MG | 3 |  | 0.4362 |  |  | |  |  |  |  |  |  |  |  |
| 33 | 1 year | sheep | the growing season | MG | 3 |  | 0.9349 |  |  | |  |  |  |  |  |  |  |  |
| 33 | 1 year | sheep | the growing season | MG | 3 |  | 0.7408 |  |  | |  |  |  |  |  |  |  |  |
| 33 | 1 year | sheep | the growing season | MG | 3 |  | 0.3027 |  |  | |  |  |  |  |  |  |  |  |
| 33 | 1 year | sheep | the growing season | MG | 3 |  | 0.7848 |  |  | |  |  |  |  |  |  |  |  |
| 33 | 1 year | sheep | the growing season | MG | 3 |  | 0.6333 |  |  | |  |  |  |  |  |  |  |  |
| 34 | ＞5 years | mixed | annual | LG | 3 | -0.1188 | 0.308 | 0.4049 | 0.2223 | |  |  |  |  |  |  |  |  |
| 34 | ＞5 years | mixed | annual | LG | 3 |  | 0.3355 |  |  | |  |  |  |  |  |  |  |  |
| 34 | ＞5 years | mixed | annual | LG | 3 |  | 0.0894 |  |  | |  |  |  |  |  |  |  |  |
| 34 | ＞5 years | mixed | annual | LG | 3 |  | 0.3387 |  |  | |  |  |  |  |  |  |  |  |
| 34 | ＞5 years | mixed | annual | LG | 3 |  | 0.286 |  |  | |  |  |  |  |  |  |  |  |
| 34 | ＞5 years | mixed | annual | MG | 3 | -0.3544 | -0.049 | 0.1417 | -0.2375 | |  |  |  |  |  |  |  |  |
| 34 | ＞5 years | mixed | annual | MG | 3 |  | -0.3667 |  |  | |  |  |  |  |  |  |  |  |
| 34 | ＞5 years | mixed | annual | MG | 3 |  | -0.1749 |  |  | |  |  |  |  |  |  |  |  |
| 34 | ＞5 years | mixed | annual | MG | 3 |  | -1.0327 |  |  | |  |  |  |  |  |  |  |  |
| 34 | ＞5 years | mixed | annual | MG | 3 |  | -0.2128 |  |  | |  |  |  |  |  |  |  |  |
| 34 | ＞5 years | mixed | annual | HG | 3 | -0.933 | -0.3152 | 0.5555 | -0.4598 | |  |  |  |  |  |  |  |  |
| 34 | ＞5 years | mixed | annual | HG | 3 |  | -0.353 |  |  | |  |  |  |  |  |  |  |  |
| 34 | ＞5 years | mixed | annual | HG | 3 |  | -0.4934 |  |  | |  |  |  |  |  |  |  |  |
| 34 | ＞5 years | mixed | annual | HG | 3 |  | -0.6294 |  |  | |  |  |  |  |  |  |  |  |
| 34 | ＞5 years | mixed | annual | HG | 3 |  | -0.3775 |  |  | |  |  |  |  |  |  |  |  |
| 35 | ＞5 years | mixed | annual | LG | 3 | -0.1645 |  |  |  | |  |  |  |  |  |  |  |  |
| 35 | ＞5 years | mixed | annual | MG | 3 | -1 |  |  |  | |  |  |  |  |  |  |  |  |
| 35 | ＞5 years | mixed | annual | HG | 3 | -1.5268 |  |  |  | |  |  |  |  |  |  |  |  |
| 40 | ＞5 years | sheep | annual | MG | 3 | -1.2824 |  |  |  | | -0.2541 | -0.5222 | -0.242 | -1.4433 |  | 0.1834 |  | -0.565 |
| 40 | ＞5 years | sheep | annual | MG | 3 |  |  |  |  | | -0.3518 | -0.0343 | -0.3392 | -1.4241 |  | 0.0251 |  | -0.7306 |
| 40 | ＞5 years | sheep | annual | MG | 3 |  |  |  |  | | -0.3517 | -0.4904 | -0.2369 | -0.6491 |  | 0.1405 |  | -0.383 |
| 40 | ＞5 years | sheep | annual | MG | 3 |  |  |  |  | | -0.3125 | -0.3841 | -0.4667 | -0.7787 |  | -0.5103 |  | -0.1513 |
| 40 | ＞5 years | sheep | the growing season | MG | 3 | -0.8984 |  |  |  | | 0.0491 | -0.0593 | 0.0272 | -0.4987 |  | -0.0387 |  | -0.0355 |
| 40 | ＞5 years | sheep | the growing season | MG | 3 |  |  |  |  | | 0.0006 | 0.1421 | -0.0676 | -0.3488 |  | -0.0627 |  | -0.2126 |
| 40 | ＞5 years | sheep | the growing season | MG | 3 |  |  |  |  | | -0.2153 | -0.1293 | 0.0142 | -0.0579 |  | 0.0997 |  | 0.0193 |
| 40 | ＞5 years | sheep | the growing season | MG | 3 |  |  |  |  | | 0.0993 | -0.0848 | -0.0819 | -0.609 |  | -0.1972 |  | -0.0416 |
| 41 | ＞5 years | sheep | the growing season | MG | 3 | -0.6733 | -0.0232 | 0.3871 | -0.091 | |  |  |  |  |  |  |  |  |
| 41 | ＞5 years | sheep | the growing season | MG | 3 |  | -0.5327 | -1.0405 | -0.4493 | |  |  |  |  |  |  |  |  |
| 41 | ＞5 years | sheep | the growing season | MG | 3 |  | 0.2422 | -0.2353 | -0.1526 | |  |  |  |  |  |  |  |  |
| 41 | ＞5 years | sheep | the growing season | MG | 3 |  | -0.8922 | -0.2331 | -0.7352 | |  |  |  |  |  |  |  |  |
| 41 | ＞5 years | sheep | the growing season | MG | 3 |  | -0.2891 | -0.8464 | -0.1348 | |  |  |  |  |  |  |  |  |
| 41 | ＞5 years | sheep | the growing season | MG | 3 |  | -0.3695 | 1.4196 | -0.2974 | |  |  |  |  |  |  |  |  |
| 41 | ＞5 years | sheep | the growing season | MG | 3 |  | -0.1039 |  |  | |  |  |  |  |  |  |  |  |
| 41 | ＞5 years | sheep | the growing season | MG | 3 |  | -0.4561 |  |  | |  |  |  |  |  |  |  |  |
| 41 | ＞5 years | sheep | the growing season | MG | 3 |  | -0.617 |  |  | |  |  |  |  |  |  |  |  |
| 41 | ＞5 years | sheep | the growing season | MG | 3 |  | -0.4678 |  |  | |  |  |  |  |  |  |  |  |
| 41 | ＞5 years | sheep | the growing season | MG | 3 |  | 0.0605 |  |  | |  |  |  |  |  |  |  |  |
| 41 | ＞5 years | sheep | the growing season | MG | 3 |  | -0.2122 |  |  | |  |  |  |  |  |  |  |  |
| 41 | ＞5 years | sheep | the growing season | MG | 3 |  | -0.376 |  |  | |  |  |  |  |  |  |  |  |
| 41 | ＞5 years | sheep | the growing season | MG | 3 |  | -0.1139 |  |  | |  |  |  |  |  |  |  |  |
| 41 | ＞5 years | sheep | the growing season | MG | 3 |  | -0.8585 |  |  | |  |  |  |  |  |  |  |  |
| 41 | ＞5 years | sheep | the growing season | MG | 3 |  | -0.6117 |  |  | |  |  |  |  |  |  |  |  |
| 41 | ＞5 years | sheep | the growing season | MG | 3 |  | 0.1641 |  |  | |  |  |  |  |  |  |  |  |
| 41 | ＞5 years | sheep | the growing season | MG | 3 |  | -0.1443 |  |  | |  |  |  |  |  |  |  |  |
| 41 | ＞5 years | sheep | the growing season | MG | 3 | -0.3708 | -0.3185 | -0.0186 | -0.2857 | |  |  |  |  |  |  |  |  |
| 41 | ＞5 years | sheep | the growing season | MG | 3 |  | -0.2423 | 0.0537 | -0.1807 | |  |  |  |  |  |  |  |  |
| 41 | ＞5 years | sheep | the growing season | MG | 3 |  | 0.4922 | 0.965 | 0.351 | |  |  |  |  |  |  |  |  |
| 41 | ＞5 years | sheep | the growing season | MG | 3 |  | -0.2912 | 0.2657 | -0.0987 | |  |  |  |  |  |  |  |  |
| 41 | ＞5 years | sheep | the growing season | MG | 3 |  | -0.3097 | 0.47 | -0.1333 | |  |  |  |  |  |  |  |  |
| 41 | ＞5 years | sheep | the growing season | MG | 3 |  | -0.3006 | 0.2809 | -0.2363 | |  |  |  |  |  |  |  |  |
| 41 | ＞5 years | sheep | the growing season | MG | 3 |  | -0.3613 |  |  | |  |  |  |  |  |  |  |  |
| 41 | ＞5 years | sheep | the growing season | MG | 3 |  | -0.2331 |  |  | |  |  |  |  |  |  |  |  |
| 41 | ＞5 years | sheep | the growing season | MG | 3 |  | 0.2792 |  |  | |  |  |  |  |  |  |  |  |
| 41 | ＞5 years | sheep | the growing season | MG | 3 |  | 0.1502 |  |  | |  |  |  |  |  |  |  |  |
| 41 | ＞5 years | sheep | the growing season | MG | 3 |  | -0.0623 |  |  | |  |  |  |  |  |  |  |  |
| 41 | ＞5 years | sheep | the growing season | MG | 3 |  | -0.2463 |  |  | |  |  |  |  |  |  |  |  |
| 41 | ＞5 years | sheep | the growing season | MG | 3 |  | -0.0487 |  |  | |  |  |  |  |  |  |  |  |
| 41 | ＞5 years | sheep | the growing season | MG | 3 |  | 0.1806 |  |  | |  |  |  |  |  |  |  |  |
| 41 | ＞5 years | sheep | the growing season | MG | 3 |  | 0.1256 |  |  | |  |  |  |  |  |  |  |  |
| 41 | ＞5 years | sheep | the growing season | MG | 3 |  | 0.1553 |  |  | |  |  |  |  |  |  |  |  |
| 41 | ＞5 years | sheep | the growing season | MG | 3 |  | 0.4449 |  |  | |  |  |  |  |  |  |  |  |
| 41 | ＞5 years | sheep | the growing season | MG | 3 |  | 0.0041 |  |  | |  |  |  |  |  |  |  |  |
| 42 | ＞5 years | sheep | annual | EG | 3 |  |  |  |  | |  | -0.5596 |  | -0.43 |  |  |  | -0.1457 |
| 42 | ＞5 years | sheep | annual | HG | 3 |  |  |  |  | |  | -0.6466 |  | -0.3354 |  |  |  | -0.0702 |
| 43 | ＞5 years | mixed | annual | EG | 3 |  | 0.1873 | -0.2926 | 0.4 | |  |  |  |  |  |  |  |  |
| 44 | ＞5 years | mixed | annual | HG | 3 | -0.797 |  |  |  | |  |  |  |  |  |  |  |  |
| 44 | ＞5 years | mixed | non-growing season | LG | 3 | -0.2886 |  |  |  | |  |  |  |  |  |  |  |  |
| 45 | 2-5 years | sheep | annual | EG | 3 | -2.0816 | 0.2963 | 2.3779 | -1.6278 | |  |  |  |  |  |  |  |  |
| 45 | 2-5 years | sheep | annual | EG | 3 | -2.0206 | 0.619 | 2.6397 | -1.5532 | |  |  |  |  |  |  |  |  |
| 46 | 2-5 years | sheep | the growing season | LG | 3 |  |  |  |  | | -0.0377 | 0 |  |  |  |  |  |  |
| 46 | 2-5 years | sheep | the growing season | LG | 3 |  |  |  |  | |  |  |  |  |  |  |  |  |
| 46 | 2-5 years | sheep | the growing season | MG | 3 |  |  |  |  | | -0.0506 | 0 |  |  |  |  |  |  |
| 46 | 2-5 years | sheep | the growing season | HG | 3 |  |  |  |  | | -0.0637 | -0.077 |  |  |  |  |  |  |
| 47 | 2-5 years | sheep | the growing season | MG | 3 | 0.5135 | 0.0731 | -1.3411 | -0.2233 | |  |  |  |  |  |  | -0.0286 | -0.5812 |
| 47 | 2-5 years | sheep | the growing season | MG | 3 |  | -1.1657 |  |  | |  |  |  |  |  |  | 0.0697 | -1.2967 |
| 47 | 2-5 years | sheep | the growing season | MG | 3 |  | -0.9102 |  |  | |  |  |  |  |  |  | -0.0103 | -0.4031 |
| 47 | 2-5 years | sheep | the growing season | MG | 3 | -0.1602 | -0.0155 | -1.0307 | -0.7656 | |  |  |  |  |  |  | -0.0367 | -0.3751 |
| 47 | 2-5 years | sheep | the growing season | MG | 3 | 0.0096 | 0.7015 | -0.6808 | -0.419 | |  |  |  |  |  |  | -0.0042 | -0.3214 |
| 47 | 2-5 years | sheep | the growing season | MG | 3 | 0.7113 | 0.0719 | -1.294 | -0.007 | |  |  |  |  |  |  | -0.0612 | 0.0463 |
| 47 | 2-5 years | sheep | the growing season | MG | 3 |  | -1.3528 |  |  | |  |  |  |  |  |  | 0.0491 | -0.914 |
| 47 | 2-5 years | sheep | the growing season | MG | 3 |  | -2.5682 |  |  | |  |  |  |  |  |  | -0.0257 | 0.3769 |
| 47 | 2-5 years | sheep | the growing season | MG | 3 |  | -0.6596 |  |  | |  |  |  |  |  |  | -0.1017 | -0.0916 |
| 47 | 2-5 years | sheep | the growing season | MG | 3 |  | -2.1269 |  |  | |  |  |  |  |  |  | 0.0202 | -0.1495 |
| 47 | 2-5 years | sheep | the growing season | MG | 3 |  | -0.4116 |  |  | |  |  |  |  |  |  | -0.0425 | 0.7324 |
| 47 | 2-5 years | sheep | the growing season | MG | 3 |  | -0.8439 |  |  | |  |  |  |  |  |  | 0.0015 | 0.3663 |
| 48 | 2-5 years | sheep | annual | MG | 3 | -0.178 | -0.0214 | 0.0361 | -0.0241 | | -0.0916 | -0.7034 |  | -0.1926 | -0.1022 |  | 0.05 | -1.0728 |
| 48 | 2-5 years | sheep | annual | MG | 3 | -0.0575 | 0.0704 |  |  | | -0.0883 | -0.5633 |  | -0.42 | -0.0469 |  | 0.0752 | -1.1239 |
| 48 | 2-5 years | sheep | annual | MG | 3 | -0.5846 | 0.1652 |  |  | | -0.3539 | -0.334 |  | -0.2683 | 0.0636 |  | 0.076 | -0.5089 |
| 48 | 2-5 years | sheep | annual | MG | 3 |  | -0.0678 |  |  | | 0.0912 | -0.6987 |  | -0.4205 | 0.3135 |  | 0.0194 | -0.55 |
| 48 | 2-5 years | sheep | annual | MG | 3 |  | -0.0833 |  |  | |  |  |  |  |  |  |  |  |
| 48 | 2-5 years | sheep | annual | MG | 3 |  | -0.2655 |  |  | |  |  |  |  |  |  |  |  |
| 48 | 2-5 years | sheep | annual | MG | 3 |  | -0.3303 |  |  | |  |  |  |  |  |  |  |  |
| 48 | 2-5 years | sheep | the growing season | MG | 3 | 0.0882 | 0.3068 | 0.2646 | 0.2889 | | 0.1438 | -0.2885 |  | 0.0907 | 0.2517 |  | -0.2015 | -0.5697 |
| 48 | 2-5 years | sheep | the growing season | MG | 3 | 0.0423 | 0.158 |  |  | | -0.0591 | -0.3354 |  | -0.0986 | 0.3218 |  | -0.1329 | -0.4652 |
| 48 | 2-5 years | sheep | the growing season | MG | 3 | -0.4377 | 0.6734 |  |  | | 0.0868 | -0.3375 |  | 0.034 | 0.4461 |  | -0.0081 | 0.0056 |
| 48 | 2-5 years | sheep | the growing season | MG | 3 |  | 0.6848 |  |  | | -0.1478 | -0.9788 |  | -0.1564 | 0.3937 |  | -0.0847 | 0.0441 |
| 48 | 2-5 years | sheep | the growing season | MG | 3 |  | -0.1106 |  |  | |  |  |  |  |  |  |  |  |
| 48 | 2-5 years | sheep | the growing season | MG | 3 |  | 0.0381 |  |  | |  |  |  |  |  |  |  |  |
| 48 | 2-5 years | sheep | the growing season | MG | 3 |  | -0.1385 |  |  | |  |  |  |  |  |  |  |  |
| 49 | 1 year | mixed | annual | LG | 3 |  |  |  |  | |  |  |  |  |  |  |  | -0.5909 |
| 49 | 1 year | mixed | annual | LG | 3 |  |  |  |  | |  |  |  |  |  |  |  | -0.9025 |
| 49 | 1 year | mixed | annual | MG | 3 |  |  |  |  | |  |  |  |  |  |  |  | -0.7233 |
| 49 | 1 year | mixed | annual | MG | 3 |  |  |  |  | |  |  |  |  |  |  |  | -1.0348 |
| 49 | 1 year | mixed | annual | HG | 3 |  |  |  |  | |  |  |  |  |  |  |  | 0.0184 |
| 49 | 1 year | mixed | annual | HG | 3 |  |  |  |  | |  |  |  |  |  |  |  | -0.5859 |
| 49 | 1 year | mixed | annual | HG | 3 |  |  |  |  | |  |  |  |  |  |  |  | -0.2931 |
| 49 | 1 year | mixed | annual | HG | 3 |  |  |  |  | |  |  |  |  |  |  |  | -0.8974 |
| 49 | 1 year | mixed | annual | EG | 3 |  |  |  |  | |  |  |  |  |  |  |  | -1.3919 |
| 49 | 1 year | mixed | annual | EG | 3 |  |  |  |  | |  |  |  |  |  |  |  | -1.7034 |
| 49 | 1 year | mixed | non-growing season | MG | 3 |  |  |  |  | |  |  |  |  |  |  |  | -0.0902 |
| 49 | 1 year | mixed | non-growing season | MG | 3 |  |  |  |  | |  |  |  |  |  |  |  | 0.14 |
| 49 | 1 year | mixed | non-growing season | MG | 3 |  |  |  |  | |  |  |  |  |  |  |  | -0.4017 |
| 49 | 1 year | mixed | non-growing season | MG | 3 |  |  |  |  | |  |  |  |  |  |  |  | -0.1715 |
| 49 | ＞5 years | mixed | annual | LG | 3 |  |  |  |  | | -0.1409 |  |  |  |  | 0.1833 |  |  |
| 49 | ＞5 years | mixed | annual | LG | 3 |  |  |  |  | | -0.0572 |  |  |  |  | 0.074 |  |  |
| 49 | ＞5 years | mixed | annual | MG | 3 |  |  |  |  | | -0.5633 |  |  |  |  | 0.2507 |  |  |
| 49 | ＞5 years | mixed | annual | MG | 3 |  |  |  |  | | -0.4796 |  |  |  |  | 0.1414 |  |  |
| 49 | ＞5 years | mixed | annual | HG | 3 |  |  |  |  | | -0.0147 |  |  |  |  | 0.1159 |  |  |
| 49 | ＞5 years | mixed | annual | HG | 3 |  |  |  |  | | -0.7928 |  |  |  |  | 0.1894 |  |  |
| 49 | ＞5 years | mixed | annual | HG | 3 |  |  |  |  | | 0.069 |  |  |  |  | 0.0067 |  |  |
| 49 | ＞5 years | mixed | annual | HG | 3 |  |  |  |  | | -0.7091 |  |  |  |  | 0.0802 |  |  |
| 49 | ＞5 years | mixed | annual | EG | 3 |  |  |  |  | | -0.7303 |  |  |  |  | 0.177 |  |  |
| 49 | ＞5 years | mixed | annual | EG | 3 |  |  |  |  | | -0.6466 |  |  |  |  | 0.0678 |  |  |
| 49 | ＞5 years | mixed | non-growing season | MG | 3 |  |  |  |  | | -0.2378 |  |  |  |  | 0.0992 |  |  |
| 49 | ＞5 years | mixed | non-growing season | MG | 3 |  |  |  |  | | -0.068 |  |  |  |  | 0.1986 |  |  |
| 49 | ＞5 years | mixed | non-growing season | MG | 3 |  |  |  |  | | -0.1542 |  |  |  |  | -0.0101 |  |  |
| 49 | ＞5 years | mixed | non-growing season | MG | 3 |  |  |  |  | | 0.0157 |  |  |  |  | 0.0894 |  |  |
| 50 | ＞5 years | mixed | annual | HG | 3 |  |  |  |  | | 0.0081 | 0.1499 | 0.2159 | -0.0554 | -0.5922 | -0.0193 |  |  |
| 50 | ＞5 years | mixed | annual | HG | 3 |  |  |  |  | | -0.0481 | 0.0924 | -0.0101 | 0.1478 | -0.4653 | -0.0092 |  |  |
| 50 | ＞5 years | mixed | annual | HG | 3 |  |  |  |  | | 0.0418 | 0.2311 | 0.0797 | -0.1396 | -0.9417 | 0 |  |  |
| 50 | ＞5 years | mixed | non-growing season | LG | 3 |  |  |  |  | |  |  | -0.0828 | -0.4037 | -0.6467 | 0.0328 |  |  |
| 50 | ＞5 years | mixed | non-growing season | LG | 3 |  |  |  |  | |  |  | -0.0326 | 0.4585 | -0.4234 | -0.0151 |  |  |
| 50 | ＞5 years | mixed | non-growing season | LG | 3 |  |  |  |  | |  |  | 0.1966 | 0.5713 | -0.354 | -0.0367 |  |  |
| 50 | ＞5 years | mixed | non-growing season | MG | 3 |  |  |  |  | | 0.0493 | 0.0846 | -0.1841 | -0.0927 | -0.3383 | 0.0118 |  |  |
| 50 | ＞5 years | mixed | non-growing season | MG | 3 |  |  |  |  | | 0.0258 | 0.0572 | -0.3458 | -0.0248 | -0.1643 | -0.0081 |  |  |
| 50 | ＞5 years | mixed | non-growing season | MG | 3 |  |  |  |  | | -0.0302 | 0.0625 | -0.2859 | 0.2799 | -0.5174 | -0.0205 |  |  |
| 50 | ＞5 years | mixed | non-growing season | MG | 3 |  |  |  |  | | -0.0222 | 0.1769 |  |  |  |  |  |  |
| 50 | ＞5 years | mixed | non-growing season | MG | 3 |  |  |  |  | | 0.066 | 0.2927 |  |  |  |  |  |  |
| 50 | ＞5 years | mixed | non-growing season | MG | 3 |  |  |  |  | | 0.0269 | 0.2776 |  |  |  |  |  |  |
| 51 | ＞5 years | sheep | annual | MG | 6 |  |  |  |  | | -0.0741 | 0.1617 | 0.1573 | -0.0118 | -0.0486 |  | 0.0715 | -0.1948 |
| 51 | ＞5 years | sheep | annual | MG | 6 |  |  |  |  | | 0 | -0.1818 | 0.0727 | -0.2788 | 0.0857 |  | 0.0294 | -0.1114 |
| 51 | ＞5 years | sheep | annual | MG | 6 |  |  |  |  | | 0.087 | 0.05 | -0.1108 | 0.5501 | 0.1301 |  | 0.0072 | -0.1368 |
| 51 | ＞5 years | sheep | annual | MG | 6 |  |  |  |  | | -0.0741 | -0.3377 | 0.1604 | -0.2684 | -0.1082 |  | -0.0073 | -0.3165 |
| 51 | ＞5 years | sheep | annual | HG | 6 |  |  |  |  | | 0.069 | -0.5184 | 0.2213 | -0.155 | 0.0434 |  | 0.1054 | -0.3912 |
| 51 | ＞5 years | sheep | annual | HG | 6 |  |  |  |  | | 0 | -0.1845 | 0.3602 | 0.3919 | -0.0703 |  | 0.0783 | -0.2989 |
| 51 | ＞5 years | sheep | annual | HG | 6 |  |  |  |  | | 0.1542 | -0.2103 | -0.0162 | 0.2154 | 0.1725 |  | 0.0858 | -0.5071 |
| 51 | ＞5 years | sheep | annual | HG | 6 |  |  |  |  | | 0.2231 | -0.1556 | 0.0733 | -0.003 | 0.3473 |  | 0.0789 | -0.4681 |
| 51 | ＞5 years | sheep | annual | HG | 6 |  |  |  |  | | 0.087 | 0.31 | -0.0799 | 0.9675 | 0.1301 |  | 0.0286 | -0.2826 |
| 51 | ＞5 years | sheep | annual | HG | 6 |  |  |  |  | | 0.4353 | -0.0015 | 0.0729 | 1.5267 | 0.5326 |  | 0.0144 | -0.226 |
| 51 | ＞5 years | sheep | annual | HG | 6 |  |  |  |  | | 0.1335 | -0.1825 | 0.2571 | 0.1116 | 0.2544 |  | 0.0144 | -0.0204 |
| 51 | ＞5 years | sheep | annual | HG | 6 |  |  |  |  | | 0.069 | -0.6404 | -0.0548 | 0.256 | 0.3928 |  | 0.0072 | 0 |
| 51 | ＞5 years | sheep | the growing season | HG | 6 |  |  |  |  | | 0.1942 | 0.0997 | 0.4049 | 0.1399 | 0.1605 |  | 0.0292 | -0.17 |
| 51 | ＞5 years | sheep | the growing season | HG | 6 |  |  |  |  | | -0.0741 | -1.0924 | -0.1097 | -0.2557 | -0.1192 |  | 0.1186 | -0.1457 |
| 51 | ＞5 years | sheep | the growing season | HG | 6 |  |  |  |  | | 0 | 0.6764 | -0.1482 | 0.1805 | 0.1308 |  | 0.058 | -0.3213 |
| 51 | ＞5 years | sheep | the growing season | HG | 6 |  |  |  |  | | 0.1542 | -0.0834 | -0.2263 | -0.2957 | 0.1742 |  | 0.0926 | -0.1386 |
| 51 | ＞5 years | sheep | the growing season | HG | 6 |  |  |  |  | | 0.1671 | 0.339 | -0.074 | 1.3972 | 0.1513 |  | 0.0286 | -0.2356 |
| 51 | ＞5 years | sheep | the growing season | HG | 6 |  |  |  |  | | 0.3102 | 0.38 | -0.1476 | 0.3053 | 0.3774 |  | 0 | -0.1861 |
| 51 | ＞5 years | sheep | the growing season | HG | 6 |  |  |  |  | | -0.0741 | -0.8556 | 0.1152 | -0.5523 | -0.0943 |  | 0.0072 | -0.3446 |
| 51 | ＞5 years | sheep | the growing season | HG | 6 |  |  |  |  | | -0.1542 | -0.0832 | 0.2374 | -0.0399 | -0.1437 |  | 0.0072 | -0.4201 |
| 52 | 1 year | sheep | the growing season | LG | 3 |  |  |  |  | |  |  |  | 0 | -0.0888 | -0.0145 | -0.0009 | 0.3128 |
| 52 | 1 year | sheep | the growing season | LG | 3 |  |  |  |  | |  |  |  | 0.6437 | -0.171 | -0.0251 |  | 0.1467 |
| 52 | 1 year | sheep | the growing season | MG | 3 |  |  |  |  | |  |  |  | -0.3029 | -0.1296 | 0.0013 | 0.0193 | 0.0882 |
| 52 | 1 year | sheep | the growing season | MG | 3 |  |  |  |  | |  |  |  | 0.2238 | -0.1235 | -0.015 |  | 0.1198 |
| 52 | 1 year | sheep | the growing season | HG | 3 |  |  |  |  | |  |  |  | 0.2579 | -0.114 | 0.0232 | -0.0009 | 0.0161 |
| 52 | 1 year | sheep | the growing season | HG | 3 |  |  |  |  | |  |  |  | 0.8212 | -0.1215 | 0.0099 |  | 0.0376 |
| 52 | 2-5 years | sheep | the growing season | LG | 3 |  |  |  |  | |  |  |  |  |  |  | 0.0148 |  |
| 52 | 2-5 years | sheep | the growing season | MG | 3 |  |  |  |  | |  |  |  |  |  |  | -0.0254 |  |
| 52 | 2-5 years | sheep | the growing season | HG | 3 |  |  |  |  | |  |  |  |  |  |  | -0.0086 |  |
| 52 | ＞5 years | sheep | the growing season | LG | 3 |  |  |  |  | |  |  |  | 0.0565 | 0.1944 | 0.0063 |  | -0.08 |
| 52 | ＞5 years | sheep | the growing season | LG | 3 |  |  |  |  | |  |  |  | -0.1354 | 0.3106 | 0.0063 |  | -0.0676 |
| 52 | ＞5 years | sheep | the growing season | MG | 3 |  |  |  |  | |  |  |  | -0.0436 | -0.0146 | 0.0089 |  | -0.1186 |
| 52 | ＞5 years | sheep | the growing season | MG | 3 |  |  |  |  | |  |  |  | -0.3864 | -0.035 | 0.0089 |  | -0.095 |
| 52 | ＞5 years | sheep | the growing season | MG | 3 |  |  |  |  | |  |  |  |  |  |  |  |  |
| 52 | ＞5 years | sheep | the growing season | MG | 3 |  |  |  |  | |  |  |  |  |  |  |  |  |
| 52 | ＞5 years | sheep | the growing season | MG | 3 |  |  |  |  | |  |  |  |  |  |  |  |  |
| 52 | ＞5 years | sheep | the growing season | MG | 3 |  |  |  |  | |  |  |  |  |  |  |  |  |
| 52 | ＞5 years | sheep | the growing season | MG | 3 |  |  |  |  | |  |  |  |  |  |  |  |  |
| 52 | ＞5 years | sheep | the growing season | MG | 3 |  |  |  |  | |  |  |  |  |  |  |  |  |
| 52 | ＞5 years | sheep | the growing season | HG | 3 |  |  |  |  | |  |  |  | -0.435 | -0.0559 | 0.0101 |  | -0.1464 |
| 52 | ＞5 years | sheep | the growing season | HG | 3 |  |  |  |  | |  |  |  | -0.4986 | -0.0603 | 0.0101 |  | -0.1296 |
| 53 | 1 year | sheep | the growing season | LG | 3 |  |  |  |  | |  |  | 0.0448 |  |  |  |  |  |
| 53 | 1 year | sheep | the growing season | LG | 3 |  |  |  |  | |  |  | 0.0456 |  |  |  |  |  |
| 53 | 1 year | sheep | the growing season | LG | 3 |  |  |  |  | |  |  | 0.2449 |  |  |  |  |  |
| 53 | 1 year | sheep | the growing season | LG | 3 |  |  |  |  | |  |  | 0.1349 |  |  |  |  |  |
| 53 | 1 year | sheep | the growing season | LG | 3 |  |  |  |  | |  |  | -0.0529 |  |  |  |  |  |
| 53 | 1 year | sheep | the growing season | MG | 3 |  |  |  |  | |  |  | -0.0591 |  |  |  |  |  |
| 53 | 1 year | sheep | the growing season | MG | 3 |  |  |  |  | |  |  | -0.0655 |  |  |  |  |  |
| 53 | 1 year | sheep | the growing season | MG | 3 |  |  |  |  | |  |  | 0.239 |  |  |  |  |  |
| 53 | 1 year | sheep | the growing season | MG | 3 |  |  |  |  | |  |  | 0.1437 |  |  |  |  |  |
| 53 | 1 year | sheep | the growing season | MG | 3 |  |  |  |  | |  |  | -0.1723 |  |  |  |  |  |
| 53 | 1 year | sheep | the growing season | HG | 3 |  |  |  |  | |  |  | -0.1947 |  |  |  |  |  |
| 53 | 1 year | sheep | the growing season | HG | 3 |  |  |  |  | |  |  | -0.1626 |  |  |  |  |  |
| 53 | 1 year | sheep | the growing season | HG | 3 |  |  |  |  | |  |  | 0.1374 |  |  |  |  |  |
| 53 | 1 year | sheep | the growing season | HG | 3 |  |  |  |  | |  |  | 0.0802 |  |  |  |  |  |
| 53 | 1 year | sheep | the growing season | HG | 3 |  |  |  |  | |  |  | -0.1409 |  |  |  |  |  |
| 54 | ＞5 years | mixed | annual | HG | 3 | -0.4932 | -0.236 | 0.5553 | -0.263 | |  |  |  |  |  |  |  | -0.0128 |
| 54 | ＞5 years | mixed | annual | HG | 3 | -0.7913 | -0.4059 |  |  | |  |  |  |  |  |  |  | -0.1505 |
| 55 | ＞5 years | mixed | annual | LG | 7 | -0.1448 | -0.1148 | 0.2509 | -0.1566 | | -0.2513 |  |  |  |  | 0.0272 | 0.0402 |  |
| 55 | ＞5 years | mixed | annual | LG | 7 | -0.3657 |  |  |  | |  |  |  |  |  |  |  |  |
| 56 | ＞5 years | mixed | annual | LG | 3 | 0.1201 | 0.0735 | -0.0644 | 0.061 | |  |  |  |  |  |  | 0.1836 |  |
| 56 | ＞5 years | mixed | annual | LG | 3 |  | 0.0356 |  |  | |  |  |  |  |  |  | 0.2279 |  |
| 56 | ＞5 years | mixed | annual | LG | 3 |  | 0.0506 |  |  | |  |  |  |  |  |  | 0.3137 |  |
| 56 | ＞5 years | mixed | annual | LG | 3 |  | 0.0333 |  |  | |  |  |  |  |  |  | 0.3443 |  |
| 56 | ＞5 years | mixed | annual | MG | 3 | -0.4041 | -0.1365 | 0.1403 | -0.2742 | |  |  |  |  |  |  | 0.2461 |  |
| 56 | ＞5 years | mixed | annual | MG | 3 |  | -0.3912 |  |  | |  |  |  |  |  |  | 0.1823 |  |
| 56 | ＞5 years | mixed | annual | MG | 3 |  | -0.4385 |  |  | |  |  |  |  |  |  | 0.2388 |  |
| 56 | ＞5 years | mixed | annual | MG | 3 |  | -0.3318 |  |  | |  |  |  |  |  |  | 0.2634 |  |
| 56 | ＞5 years | mixed | annual | HG | 3 | -0.8374 | -0.2607 | 0.4813 | -0.3867 | |  |  |  |  |  |  | 0.19 |  |
| 56 | ＞5 years | mixed | annual | HG | 3 |  | -0.4347 |  |  | |  |  |  |  |  |  | 0.1133 |  |
| 56 | ＞5 years | mixed | annual | HG | 3 |  | -0.4778 |  |  | |  |  |  |  |  |  | 0.0941 |  |
| 56 | ＞5 years | mixed | annual | HG | 3 |  | -0.4376 |  |  | |  |  |  |  |  |  | 0.027 |  |
| 57 | 2-5 years | mixed | annual | EG | 3 |  |  |  |  | | -0.1098 | -0.0785 | -0.0307 | 1.1012 | 0.1274 | 0.0399 | -0.0854 |  |
| 57 | 2-5 years | mixed | annual | EG | 3 |  |  |  |  | | 0.1062 | -0.0483 | -0.0056 | 0.6451 | 0.3557 | 0.0245 | -0.0603 |  |
| 57 | 2-5 years | mixed | annual | EG | 3 |  |  |  |  | | 0.1275 | 0.0094 | 0.0406 | 0.5607 | 0.2961 | 0.0175 | -0.0479 |  |
| 58 | 2-5 years | sheep | the growing season | LG | 3 |  |  |  |  | | 0.0924 | -0.0625 | 0.1281 | 0.4495 | -0.1077 | -0.035 |  |  |
| 58 | 2-5 years | sheep | the growing season | HG | 3 |  |  |  |  | | 0.1769 | 0.087 | 0.1133 | 0.5635 | 0.1061 | -0.0143 |  |  |
| 59 | 2-5 years | sheep | the growing season | LG | 3 |  |  |  |  | | -0.029 | 0.026 | -0.0776 | 0.1542 | -0.0015 |  |  |  |
| 59 | 2-5 years | sheep | the growing season | LG | 3 |  |  |  |  | | -0.0822 | -0.0723 | -0.0102 | 0.047 | -0.0859 |  |  |  |
| 59 | 2-5 years | sheep | the growing season | LG | 3 |  |  |  |  | | 0.0896 | 0.2183 | 0.0049 | -0.2151 | 0.0487 |  |  |  |
| 59 | 2-5 years | sheep | the growing season | LG | 3 |  |  |  |  | | -0.1268 | -0.0488 | -0.1406 | 0.0937 | -0.1493 |  |  |  |
| 59 | 2-5 years | sheep | the growing season | LG | 3 |  |  |  |  | | -0.1542 | -0.0476 | -0.0315 | -0.2384 | -0.1515 |  |  |  |
| 59 | 2-5 years | sheep | the growing season | LG | 3 |  |  |  |  | | 0 | 0.0377 | -0.0101 | -0.1335 | -0.0219 |  |  |  |
| 59 | 2-5 years | sheep | the growing season | LG | 3 |  |  |  |  | | 0.0465 | 0 | -0.0239 | 0.2582 | 0.1227 |  |  |  |
| 59 | 2-5 years | sheep | the growing season | LG | 3 |  |  |  |  | | -0.1178 | 0.0918 | -0.0558 | 0.0381 | -0.0227 |  |  |  |
| 59 | 2-5 years | sheep | the growing season | LG | 3 |  |  |  |  | | -0.023 | 0.0991 | 0.0457 | 0.0541 | -0.0453 |  |  |  |
| 59 | 2-5 years | sheep | the growing season | LG | 3 |  |  |  |  | | -0.1769 | -0.1671 | 0.05 | 0.1759 | -0.1952 |  |  |  |
| 59 | 2-5 years | sheep | the growing season | LG | 3 |  |  |  |  | | -0.1178 | -0.0822 | 0.2422 | -0.0513 | -0.051 |  |  |  |
| 59 | 2-5 years | sheep | the growing season | LG | 3 |  |  |  |  | | -0.087 | -0.4162 | -0.0309 | -0.0243 | -0.2067 |  |  |  |
| 59 | 2-5 years | sheep | the growing season | LG | 3 |  |  |  |  | | -0.1214 | -0.1001 | -0.1341 | 0.2076 | -0.0315 |  |  |  |
| 59 | 2-5 years | sheep | the growing season | LG | 3 |  |  |  |  | | -0.1625 | -0.1452 | -0.0941 | 0.0831 | -0.1153 |  |  |  |
| 59 | 2-5 years | sheep | the growing season | LG | 3 |  |  |  |  | | -0.1112 | -0.08 | 0.2204 | 0.0986 | -0.1628 |  |  |  |
| 59 | 2-5 years | sheep | the growing season | LG | 3 |  |  |  |  | | -0.0572 | -0.2549 | -0.0568 | 0 | -0.1045 |  |  |  |
| 59 | 2-5 years | sheep | the growing season | LG | 3 |  |  |  |  | | -0.1178 | -0.1366 | -0.1216 | 0.1095 | 0.0219 |  |  |  |
| 59 | 2-5 years | sheep | the growing season | LG | 3 |  |  |  |  | | -0.1335 | -0.0606 | -0.0009 | 0.2877 | -0.0898 |  |  |  |
| 59 | 2-5 years | sheep | the growing season | LG | 3 |  |  |  |  | | -0.2231 | -0.0274 | 0.12 | -0.0922 | -0.0591 |  |  |  |
| 59 | 2-5 years | sheep | the growing season | LG | 3 |  |  |  |  | | -0.0282 | -0.2624 | -0.1111 | 0.0827 | -0.0783 |  |  |  |
| 59 | 2-5 years | sheep | the growing season | LG | 3 |  |  |  |  | | -0.087 | -0.0953 | -0.1427 | -0.0078 | -0.0839 |  |  |  |
| 59 | 2-5 years | sheep | the growing season | MG | 3 |  |  |  |  | | -0.0896 | 0 | -0.1404 | 0.0165 | -0.003 |  |  |  |
| 59 | 2-5 years | sheep | the growing season | MG | 3 |  |  |  |  | | -0.1411 | 0.0674 | -0.0028 | -0.0194 | -0.0617 |  |  |  |
| 59 | 2-5 years | sheep | the growing season | MG | 3 |  |  |  |  | | 0.0308 | 0.0476 | -0.1921 | -0.2476 | 0 |  |  |  |
| 59 | 2-5 years | sheep | the growing season | MG | 3 |  |  |  |  | | -0.2113 | -0.1001 | -0.1178 | 0.1098 | -0.163 |  |  |  |
| 59 | 2-5 years | sheep | the growing season | MG | 3 |  |  |  |  | | -0.1823 | -0.0976 | 0.0137 | -0.1915 | -0.0782 |  |  |  |
| 59 | 2-5 years | sheep | the growing season | MG | 3 |  |  |  |  | | -0.1112 | -0.1671 | -0.1892 | -0.0805 | -0.1202 |  |  |  |
| 59 | 2-5 years | sheep | the growing season | MG | 3 |  |  |  |  | | -0.1268 | -0.0976 | -0.1514 | 0.1719 | -0.0382 |  |  |  |
| 59 | 2-5 years | sheep | the growing season | MG | 3 |  |  |  |  | | -0.2513 | -0.1226 | -0.1417 | -0.1349 | -0.2285 |  |  |  |
| 59 | 2-5 years | sheep | the growing season | MG | 3 |  |  |  |  | | -0.2007 | -0.0426 | 0.0087 | -0.0572 | -0.1792 |  |  |  |
| 59 | 2-5 years | sheep | the growing season | MG | 3 |  |  |  |  | | -0.2097 | -0.1978 | -0.1586 | 0.2915 | -0.2889 |  |  |  |
| 59 | 2-5 years | sheep | the growing season | MG | 3 |  |  |  |  | | -0.1495 | -0.0541 | 0.1255 | -0.3646 | -0.1121 |  |  |  |
| 59 | 2-5 years | sheep | the growing season | MG | 3 |  |  |  |  | | -0.1495 | -0.4162 | 0.0502 | -0.1009 | -0.2522 |  |  |  |
| 59 | 2-5 years | sheep | the growing season | MG | 3 |  |  |  |  | | -0.2231 | -0.0241 | -0.0574 | -0.08 | -0.1097 |  |  |  |
| 59 | 2-5 years | sheep | the growing season | MG | 3 |  |  |  |  | | -0.2231 | -0.1769 | -0.119 | 0.1116 | -0.2157 |  |  |  |
| 59 | 2-5 years | sheep | the growing season | MG | 3 |  |  |  |  | | -0.2036 | -0.0526 | 0.0823 | -0.2247 | -0.151 |  |  |  |
| 59 | 2-5 years | sheep | the growing season | MG | 3 |  |  |  |  | | -0.087 | -0.3567 | -0.1245 | -0.174 | -0.0523 |  |  |  |
| 59 | 2-5 years | sheep | the growing season | MG | 3 |  |  |  |  | | -0.1178 | -0.2126 | -0.0477 | -0.0596 | -0.0614 |  |  |  |
| 59 | 2-5 years | sheep | the growing season | MG | 3 |  |  |  |  | | -0.0253 | 0.029 | -0.2216 | 0.3015 | -0.0585 |  |  |  |
| 59 | 2-5 years | sheep | the growing season | MG | 3 |  |  |  |  | | -0.1924 | 0.0526 | 0.0396 | -0.2982 | -0.0404 |  |  |  |
| 59 | 2-5 years | sheep | the growing season | MG | 3 |  |  |  |  | | -0.1178 | -0.1082 | 0 | -0.1997 | -0.1296 |  |  |  |
| 59 | 2-5 years | sheep | the growing season | MG | 3 |  |  |  |  | | -0.087 | -0.023 | -0.1477 | -0.1425 | -0.0335 |  |  |  |
| 59 | 2-5 years | sheep | the growing season | HG | 3 |  |  |  |  | | 0 | 0 | -0.0684 | -0.0339 | 0.0802 |  |  |  |
| 59 | 2-5 years | sheep | the growing season | HG | 3 |  |  |  |  | | -0.1411 | 0.0455 | -0.0204 | -0.0097 | -0.0163 |  |  |  |
| 59 | 2-5 years | sheep | the growing season | HG | 3 |  |  |  |  | | 0 | 0 | -0.0463 | -0.1308 | -0.0222 |  |  |  |
| 59 | 2-5 years | sheep | the growing season | HG | 3 |  |  |  |  | | -0.1268 | -0.0741 | -0.0419 | 0.1335 | -0.2391 |  |  |  |
| 59 | 2-5 years | sheep | the growing season | HG | 3 |  |  |  |  | | -0.0488 | 0.023 | -0.0342 | -0.1823 | 0.1052 |  |  |  |
| 59 | 2-5 years | sheep | the growing season | HG | 3 |  |  |  |  | | -0.0822 | -0.1671 | 0.0253 | -0.2113 | -0.0961 |  |  |  |
| 59 | 2-5 years | sheep | the growing season | HG | 3 |  |  |  |  | | -0.1268 | -0.0723 | -0.0109 | -0.1133 | 0.0069 |  |  |  |
| 59 | 2-5 years | sheep | the growing season | HG | 3 |  |  |  |  | | -0.1178 | -0.0392 | -0.1148 | -0.1804 | -0.1276 |  |  |  |
| 59 | 2-5 years | sheep | the growing season | HG | 3 |  |  |  |  | | -0.0706 | -0.0211 | -0.0343 | -0.0795 | -0.1181 |  |  |  |
| 59 | 2-5 years | sheep | the growing season | HG | 3 |  |  |  |  | | 0.0526 | -0.1372 | -0.1504 | 0.2262 | -0.0444 |  |  |  |
| 59 | 2-5 years | sheep | the growing season | HG | 3 |  |  |  |  | | -0.1823 | -0.1411 | 0.2502 | -0.3178 | -0.0428 |  |  |  |
| 59 | 2-5 years | sheep | the growing season | HG | 3 |  |  |  |  | | -0.1495 | -0.2948 | 0.1303 | -0.0576 | -0.192 |  |  |  |
| 59 | 2-5 years | sheep | the growing season | HG | 3 |  |  |  |  | | -0.2595 | 0.0235 | 0.041 | 0.0418 | -0.1112 |  |  |  |
| 59 | 2-5 years | sheep | the growing season | HG | 3 |  |  |  |  | | -0.1335 | -0.1144 | 0.0107 | -0.0402 | -0.2095 |  |  |  |
| 59 | 2-5 years | sheep | the growing season | HG | 3 |  |  |  |  | | -0.1411 | -0.1082 | 0.1331 | -0.2401 | -0.0912 |  |  |  |
| 59 | 2-5 years | sheep | the growing season | HG | 3 |  |  |  |  | | -0.087 | 0 | -0.1365 | -0.0342 | -0.0495 |  |  |  |
| 59 | 2-5 years | sheep | the growing season | HG | 3 |  |  |  |  | | -0.2513 | -0.2126 | 0.0681 | 0.0164 | -0.0324 |  |  |  |
| 59 | 2-5 years | sheep | the growing season | HG | 3 |  |  |  |  | | -0.1054 | 0.029 | -0.2149 | 0.1382 | -0.1495 |  |  |  |
| 59 | 2-5 years | sheep | the growing season | HG | 3 |  |  |  |  | | 0.0247 | 0.0267 | 0.188 | -0.2326 | -0.0319 |  |  |  |
| 59 | 2-5 years | sheep | the growing season | HG | 3 |  |  |  |  | | -0.0572 | -0.026 | -0.0409 | -0.0087 | -0.0712 |  |  |  |
| 59 | 2-5 years | sheep | the growing season | HG | 3 |  |  |  |  | | -0.1178 | -0.0465 | -0.0341 | -0.1515 | -0.0556 |  |  |  |
| 60 | ＞5 years | mixed | the growing season | EG | 3 |  |  |  |  | | -0.2228 | -0.3971 |  |  | -0.232 | 0.2077 | 0.5508 | -0.3349 |
| 60 | ＞5 years | mixed | the growing season | EG | 3 |  |  |  |  | | -0.1778 | -0.3485 |  |  | -0.2003 | 0.1922 | 0.4999 | -0.2896 |
| 60 | ＞5 years | mixed | the growing season | EG | 3 |  |  |  |  | | -0.151 | -0.2522 |  |  | -0.1448 | 0.106 | 0.3305 | -0.2438 |
| 60 | ＞5 years | mixed | the growing season | EG | 3 |  |  |  |  | | -0.1303 | -0.1843 |  |  | -0.1167 | 0.1339 | 0.3011 | -0.2209 |
| 60 | ＞5 years | mixed | the growing season | EG | 3 |  |  |  |  | | -0.0901 | -0.1571 |  |  | -0.0836 | 0.1316 | 0.2581 | -0.1318 |
| 60 | ＞5 years | mixed | the growing season | EG | 3 |  |  |  |  | | -0.0514 | -0.1127 |  |  | -0.0487 | 0.0529 | 0.1165 | -0.0609 |
| 60 | ＞5 years | mixed | the growing season | EG | 3 |  |  |  |  | | -0.0094 | -0.1049 |  |  | -0.0506 | 0.0695 | 0.1938 | -0.0364 |
| 60 | ＞5 years | mixed | the growing season | EG | 3 |  |  |  |  | | 0.0016 | 0.0018 |  |  | 0.0321 | 0.0059 | 0.1054 | -0.0072 |
| 60 | ＞5 years | mixed | the growing season | EG | 3 |  |  |  |  | | -0.1339 | -0.494 |  |  | -0.1834 | 0.1355 | 0.1534 | -0.3235 |
| 60 | ＞5 years | mixed | the growing season | EG | 3 |  |  |  |  | | -0.1455 | -0.4343 |  |  | -0.2337 | 0.1298 | 0.1784 | -0.269 |
| 60 | ＞5 years | mixed | the growing season | EG | 3 |  |  |  |  | | -0.1238 | -0.3989 |  |  | -0.0671 | 0.0825 | 0.0191 | -0.2973 |
| 60 | ＞5 years | mixed | the growing season | EG | 3 |  |  |  |  | | -0.079 | -0.3316 |  |  | -0.1071 | 0.089 | 0.1233 | -0.2223 |
| 60 | ＞5 years | mixed | the growing season | EG | 3 |  |  |  |  | | -0.0817 | -0.2346 |  |  | -0.1403 | 0.089 | 0.0678 | -0.1411 |
| 60 | ＞5 years | mixed | the growing season | EG | 3 |  |  |  |  | | -0.048 | -0.2118 |  |  | -0.1094 | 0.0507 | 0.1097 | -0.1079 |
| 60 | ＞5 years | mixed | the growing season | EG | 3 |  |  |  |  | | -0.0427 | -0.1082 |  |  | -0.0795 | 0.0006 | 0.0848 | -0.0365 |
| 60 | ＞5 years | mixed | the growing season | EG | 3 |  |  |  |  | | -0.0309 | -0.0247 |  |  | -0.0406 | 0.0403 | 0.0231 | 0.0243 |
| 61 | 1 year | cattle | the growing season | MG | 3 | -0.2297 |  |  |  | | -0.0215 | -0.4563 | 0.497 | -0.1621 | -0.1033 |  | -0.0064 | -0.0374 |
| 61 | 1 year | cattle | the growing season | MG | 3 |  |  |  |  | | -0.2933 | -0.4857 | -0.3043 | 0.0705 | -0.0281 |  | 0.0171 | -0.0879 |
| 61 | 1 year | cattle | the growing season | MG | 3 |  |  |  |  | | 0.0189 | 0.3093 | -0.1823 | -0.3124 | -0.023 |  | 0.0471 | 0.0089 |
| 61 | 1 year | cattle | the growing season | HG | 3 | -0.4375 |  |  |  | | -0.0569 | -0.5785 | 0.5325 | -0.5464 | -0.2216 |  | 0.1692 | 0.0468 |
| 61 | 1 year | cattle | the growing season | HG | 3 |  |  |  |  | | -0.3717 | -0.5498 | -0.2134 | 0.3687 | -0.2462 |  | 0.1214 | 0.0131 |
| 61 | 1 year | cattle | the growing season | HG | 3 |  |  |  |  | | -0.1677 | -0.1549 | -0.1267 | -0.32 | -0.0473 |  | 0.0828 | -0.0312 |
| 62 | 1 year | cattle | the growing season | LG | 3 | -0.1714 | -0.1131 | 0.0583 | -0.1423 | |  |  |  |  |  |  | -0.08 | -0.0303 |
| 62 | 1 year | cattle | the growing season | MG | 3 | -0.0843 | -0.2713 | -0.187 | -0.1718 | |  |  |  |  |  |  | 0.1092 | -0.0188 |
| 62 | 1 year | cattle | the growing season | HG | 3 | 0.0185 | -0.0269 | -0.0454 | -0.0035 | |  |  |  |  |  |  | 0.0918 | -0.1318 |
| 62 | 1 year | cattle | the growing season | EG | 3 | -0.5421 | 0.0994 | 0.6415 | -0.1762 | |  |  |  |  |  |  | -0.0905 | -0.0069 |
| 63 | ＞5 years | mixed | the growing season | EG | 3 |  |  |  |  | | 0.6019 | -0.071 | 0.2687 |  | 0.277 | 0.0012 |  | -0.3896 |
| 63 | ＞5 years | mixed | the growing season | EG | 3 |  |  |  |  | | 0.3588 | -0.0028 | 0.0452 |  | -0.032 | -0.0322 |  | -0.0314 |
| 63 | ＞5 years | mixed | the growing season | EG | 3 |  |  |  |  | | -0.317 | -0.0342 | 0.4549 |  | -0.261 | 0.0497 |  | -0.2848 |
| 64 | ＞5 years | mixed | annual | LG | 3 |  |  |  |  | |  |  |  |  |  |  | -0.815 |  |
| 64 | ＞5 years | mixed | annual | LG | 3 |  |  |  |  | |  |  |  |  |  |  | -0.7044 |  |
| 64 | ＞5 years | mixed | annual | LG | 3 |  |  |  |  | |  |  |  |  |  |  | 0.3433 |  |
| 64 | ＞5 years | mixed | annual | HG | 3 |  |  |  |  | |  |  |  |  |  |  | 0.5713 |  |
| 64 | ＞5 years | mixed | annual | HG | 3 |  |  |  |  | |  |  |  |  |  |  | 0.3942 |  |
| 64 | ＞5 years | mixed | annual | HG | 3 |  |  |  |  | |  |  |  |  |  |  | 0.6116 |  |
| 65 | 1 year | cattle | the growing season | LG | 6 | 0.0276 |  |  |  | | 0.0001 | 0.1163 | 0.0109 | 0.3586 | -0.116 |  | -0.3164 |  |
| 65 | 1 year | cattle | the growing season | LG | 6 | -0.3268 |  |  |  | | -0.0037 | 0.397 | -0.0987 | 0.0802 | -0.0603 |  | -0.2556 |  |
| 65 | 1 year | cattle | the growing season | LG | 6 | -0.3562 |  |  |  | | 0.0043 | 0.0807 | -0.1827 | 0.0641 | -0.0365 |  | -0.1116 |  |
| 65 | 1 year | cattle | the growing season | LG | 6 | -0.3128 |  |  |  | | 0.0008 | -0.2475 | -0.1209 | -0.0508 | -0.0267 |  | -0.1056 |  |
| 65 | 1 year | cattle | the growing season | LG | 6 |  |  |  |  | | -0.0053 | 0.042 | 0.0166 | -0.221 | -0.0489 |  |  |  |
| 65 | 1 year | cattle | the growing season | LG | 6 |  |  |  |  | | 0.0004 | -0.2587 | -0.6694 | -0.0773 | 0.0156 |  |  |  |
| 65 | 1 year | cattle | the growing season | LG | 6 |  |  |  |  | | -0.0028 | -0.1225 | -0.3202 | -0.0675 | 0.0403 |  |  |  |
| 65 | 1 year | cattle | the growing season | LG | 6 |  |  |  |  | | -0.0044 | -0.1849 | -0.3427 | -0.0864 | 0.0222 |  |  |  |
| 65 | 1 year | cattle | the growing season | LG | 6 |  |  |  |  | | -0.0016 | -0.0475 | -0.0086 | -0.3442 | -0.073 |  |  |  |
| 65 | 1 year | cattle | the growing season | LG | 6 |  |  |  |  | | 0.0029 | 0.0577 | 0.1739 | -0.2035 | -0.0461 |  |  |  |
| 65 | 1 year | cattle | the growing season | LG | 6 |  |  |  |  | | 0.0006 | 0.0456 | 0.0377 | -0.0099 | 0.1549 |  |  |  |
| 65 | 1 year | cattle | the growing season | LG | 6 |  |  |  |  | | 0.0007 | -0.2106 | -0.1662 | 0.0057 | -0.0695 |  |  |  |
| 65 | 1 year | cattle | the growing season | MG | 6 | 0.0953 |  |  |  | | -0.0554 | 0.0674 | 0.004 | 0.7816 | -0.0851 |  | -0.0898 |  |
| 65 | 1 year | cattle | the growing season | MG | 6 | -0.3614 |  |  |  | | -0.2934 | 0.1558 | -0.1231 | 0.4365 | -0.0418 |  | -0.0572 |  |
| 65 | 1 year | cattle | the growing season | MG | 6 | -0.3668 |  |  |  | | -0.0168 | -0.0212 | -0.0665 | 0.011 | -0.0181 |  | -0.0219 |  |
| 65 | 1 year | cattle | the growing season | MG | 6 | -0.3387 |  |  |  | | -0.2591 | 0.1434 | -0.2037 | 0.0313 | -0.0769 |  | -0.0292 |  |
| 65 | 1 year | cattle | the growing season | MG | 6 |  |  |  |  | | -0.0116 | -0.0173 | -0.01 | -0.2001 | 0.1068 |  |  |  |
| 65 | 1 year | cattle | the growing season | MG | 6 |  |  |  |  | | 0.052 | -0.5989 | -0.1556 | -0.0389 | -0.0066 |  |  |  |
| 65 | 1 year | cattle | the growing season | MG | 6 |  |  |  |  | | 0.1398 | -0.1104 | -0.3011 | 0.0209 | 0.038 |  |  |  |
| 65 | 1 year | cattle | the growing season | MG | 6 |  |  |  |  | | -0.1011 | -0.0329 | -0.1685 | 0.0247 | -0.0861 |  |  |  |
| 65 | 1 year | cattle | the growing season | MG | 6 |  |  |  |  | | 0.0765 | 0.0254 | 0.0915 | -0.2236 | -0.0218 |  |  |  |
| 65 | 1 year | cattle | the growing season | MG | 6 |  |  |  |  | | 0.1978 | -0.0394 | 0.1423 | -0.1863 | 0.0301 |  |  |  |
| 65 | 1 year | cattle | the growing season | MG | 6 |  |  |  |  | | 0.0541 | 0.1333 | 0.0925 | -0.0099 | 0.2311 |  |  |  |
| 65 | 1 year | cattle | the growing season | MG | 6 |  |  |  |  | | 0.1894 | -0.1612 | 0.422 | -0.0506 | 0.01 |  |  |  |
| 65 | 1 year | cattle | the growing season | HG | 6 | 0.0125 |  |  |  | | -0.0187 | -0.004 | -0.0157 | 0.5106 | -0.1111 |  | -0.3804 |  |
| 65 | 1 year | cattle | the growing season | HG | 6 | -0.5066 |  |  |  | | -0.1258 | 0.0195 | -0.1949 | 0.2397 | 0.0093 |  | -0.3264 |  |
| 65 | 1 year | cattle | the growing season | HG | 6 | -0.6081 |  |  |  | | -0.0249 | -0.319 | -0.0636 | 0.0025 | 0.0101 |  | -0.2042 |  |
| 65 | 1 year | cattle | the growing season | HG | 6 | -0.5913 |  |  |  | | -0.1876 | -0.0671 | -0.1724 | 0.0116 | -0.0052 |  | -0.1388 |  |
| 65 | 1 year | cattle | the growing season | HG | 6 |  |  |  |  | | 0.2627 | -0.1145 | 0.0898 | -0.1566 | 0.0249 |  |  |  |
| 65 | 1 year | cattle | the growing season | HG | 6 |  |  |  |  | | 0.3873 | -0.2336 | -0.6506 | -0.0202 | -0.0427 |  |  |  |
| 65 | 1 year | cattle | the growing season | HG | 6 |  |  |  |  | | 0.2225 | -0.0971 | -0.1851 | 0.0265 | 0.067 |  |  |  |
| 65 | 1 year | cattle | the growing season | HG | 6 |  |  |  |  | | 0.0893 | -0.0269 | -0.1794 | -0.0126 | -0.0817 |  |  |  |
| 65 | 1 year | cattle | the growing season | HG | 6 |  |  |  |  | | 0.2911 | -0.0083 | -0.0009 | -0.1444 | -0.0713 |  |  |  |
| 65 | 1 year | cattle | the growing season | HG | 6 |  |  |  |  | | 0.5309 | -0.0808 | -0.0316 | -0.0879 | 0.0219 |  |  |  |
| 65 | 1 year | cattle | the growing season | HG | 6 |  |  |  |  | | 0.5292 | -0.3256 | 0.0975 | -0.0469 | 0.1255 |  |  |  |
| 65 | 1 year | cattle | the growing season | HG | 6 |  |  |  |  | | 0.2868 | -0.1232 | -0.0984 | -0.0291 | 0.0332 |  |  |  |
| 66 | 1 year | cattle | the growing season | LG | 3 | -0.2604 |  |  |  | | -0.0299 |  |  |  | -0.0179 |  |  |  |
| 66 | 1 year | cattle | the growing season | LG | 3 |  |  |  |  | | -0.0198 |  |  |  | 0.0308 |  |  |  |
| 66 | 1 year | cattle | the growing season | LG | 3 |  |  |  |  | | 0.0741 |  |  |  | -0.1361 |  |  |  |
| 66 | 1 year | cattle | the growing season | MG | 3 | -0.446 |  |  |  | | -0.0711 |  |  |  | 0.0018 |  |  |  |
| 66 | 1 year | cattle | the growing season | MG | 3 |  |  |  |  | | 0.0194 |  |  |  | -0.0318 |  |  |  |
| 66 | 1 year | cattle | the growing season | MG | 3 |  |  |  |  | | 0.0377 |  |  |  | 0.1491 |  |  |  |
| 66 | 1 year | cattle | the growing season | HG | 3 | -0.7894 |  |  |  | | -0.0924 |  |  |  | -0.0979 |  |  |  |
| 66 | 1 year | cattle | the growing season | HG | 3 |  |  |  |  | | -0.0606 |  |  |  | -0.0757 |  |  |  |
| 66 | 1 year | cattle | the growing season | HG | 3 |  |  |  |  | | 0 |  |  |  | 0.0222 |  |  |  |
| 66 | 2-5 years | cattle | the growing season | LG | 3 | -0.3306 |  |  |  | | -0.0503 |  |  |  | -0.0118 |  |  |  |
| 66 | 2-5 years | cattle | the growing season | LG | 3 | -0.6088 |  |  |  | | -0.0392 |  |  |  | -0.0103 |  |  |  |
| 66 | 2-5 years | cattle | the growing season | LG | 3 |  |  |  |  | | -0.0198 |  |  |  | 0.008 |  |  |  |
| 66 | 2-5 years | cattle | the growing season | LG | 3 |  |  |  |  | | 0.0972 |  |  |  | 0.0315 |  |  |  |
| 66 | 2-5 years | cattle | the growing season | LG | 3 |  |  |  |  | | 0 |  |  |  | 0.0125 |  |  |  |
| 66 | 2-5 years | cattle | the growing season | LG | 3 |  |  |  |  | | 0.08 |  |  |  | 0.0457 |  |  |  |
| 66 | 2-5 years | cattle | the growing season | MG | 6 | -0.5763 |  |  |  | | -0.0099 |  |  |  | 0.0055 |  |  |  |
| 66 | 2-5 years | cattle | the growing season | MG | 6 | -0.6242 |  |  |  | | -0.0594 |  |  |  | -0.0092 |  |  |  |
| 66 | 2-5 years | cattle | the growing season | MG | 3 |  |  |  |  | | 0 |  |  |  | 0.0057 |  |  |  |
| 66 | 2-5 years | cattle | the growing season | MG | 3 |  |  |  |  | | 0.1155 |  |  |  | -0.0298 |  |  |  |
| 66 | 2-5 years | cattle | the growing season | MG | 3 |  |  |  |  | | 0.0408 |  |  |  | 0.0139 |  |  |  |
| 66 | 2-5 years | cattle | the growing season | MG | 3 |  |  |  |  | | 0.1178 |  |  |  | 0.0421 |  |  |  |
| 66 | 2-5 years | cattle | the growing season | HG | 6 | -0.8571 |  |  |  | | -0.0711 |  |  |  | -0.062 |  |  |  |
| 66 | 2-5 years | cattle | the growing season | HG | 6 | -0.8565 |  |  |  | | -0.1118 |  |  |  | -0.1538 |  |  |  |
| 66 | 2-5 years | cattle | the growing season | HG | 3 |  |  |  |  | | -0.1942 |  |  |  | -0.1573 |  |  |  |
| 66 | 2-5 years | cattle | the growing season | HG | 3 |  |  |  |  | | -0.2029 |  |  |  | -0.2155 |  |  |  |
| 66 | 2-5 years | cattle | the growing season | HG | 3 |  |  |  |  | | 0.08 |  |  |  | -0.0428 |  |  |  |
| 66 | 2-5 years | cattle | the growing season | HG | 3 |  |  |  |  | | 0.1892 |  |  |  | 0.0762 |  |  |  |
| 67 | ＞5 years | sheep | the growing season | LG | 3 | -0.0094 | -0.2577 | -0.2483 | -0.2518 | |  |  |  |  |  |  |  |  |
| 67 | ＞5 years | sheep | the growing season | LG | 3 | -0.1965 | -0.0777 | 0.1188 | -0.0816 | |  |  |  |  |  |  |  |  |
| 67 | ＞5 years | sheep | the growing season | LG | 3 | -0.0416 | -0.2161 | -0.1745 | -0.2071 | |  |  |  |  |  |  |  |  |
| 67 | ＞5 years | sheep | the growing season | LG | 3 | -0.0386 | -0.1259 | -0.0873 | -0.1196 | |  |  |  |  |  |  |  |  |
| 67 | ＞5 years | sheep | the growing season | LG | 3 | -0.1318 | -0.056 | 0.0758 | -0.0603 | |  |  |  |  |  |  |  |  |
| 67 | ＞5 years | sheep | the growing season | LG | 3 |  | -0.278 |  |  | |  |  |  |  |  |  |  |  |
| 67 | ＞5 years | sheep | the growing season | LG | 3 |  | -0.0875 |  |  | |  |  |  |  |  |  |  |  |
| 67 | ＞5 years | sheep | the growing season | LG | 3 |  | -0.2263 |  |  | |  |  |  |  |  |  |  |  |
| 67 | ＞5 years | sheep | the growing season | LG | 3 |  | -0.1307 |  |  | |  |  |  |  |  |  |  |  |
| 67 | ＞5 years | sheep | the growing season | LG | 3 |  | -0.1095 |  |  | |  |  |  |  |  |  |  |  |
| 67 | ＞5 years | sheep | the growing season | LG | 3 |  | -0.2884 |  |  | |  |  |  |  |  |  |  |  |
| 67 | ＞5 years | sheep | the growing season | LG | 3 |  | 0.0054 |  |  | |  |  |  |  |  |  |  |  |
| 67 | ＞5 years | sheep | the growing season | LG | 3 |  | -0.2589 |  |  | |  |  |  |  |  |  |  |  |
| 67 | ＞5 years | sheep | the growing season | LG | 3 |  | -0.212 |  |  | |  |  |  |  |  |  |  |  |
| 67 | ＞5 years | sheep | the growing season | LG | 3 |  | 0.0611 |  |  | |  |  |  |  |  |  |  |  |
| 67 | ＞5 years | sheep | the growing season | LG | 3 |  | -0.1365 |  |  | |  |  |  |  |  |  |  |  |
| 67 | ＞5 years | sheep | the growing season | LG | 3 |  | -0.1285 |  |  | |  |  |  |  |  |  |  |  |
| 67 | ＞5 years | sheep | the growing season | LG | 3 |  | -0.0958 |  |  | |  |  |  |  |  |  |  |  |
| 67 | ＞5 years | sheep | the growing season | LG | 3 |  | 0.0751 |  |  | |  |  |  |  |  |  |  |  |
| 67 | ＞5 years | sheep | the growing season | LG | 3 |  | 0.0124 |  |  | |  |  |  |  |  |  |  |  |
| 67 | ＞5 years | sheep | the growing season | MG | 3 | -0.3236 | -0.3409 | -0.0173 | -0.3406 | |  |  |  |  |  |  |  |  |
| 67 | ＞5 years | sheep | the growing season | MG | 3 | -0.4633 | -0.1259 | 0.3374 | -0.1358 | |  |  |  |  |  |  |  |  |
| 67 | ＞5 years | sheep | the growing season | MG | 3 | -0.129 | -0.2976 | -0.1685 | -0.2889 | |  |  |  |  |  |  |  |  |
| 67 | ＞5 years | sheep | the growing season | MG | 3 | -0.4527 | -0.1423 | 0.3105 | -0.1612 | |  |  |  |  |  |  |  |  |
| 67 | ＞5 years | sheep | the growing season | MG | 3 | -0.5916 | -0.2252 | 0.3664 | -0.2435 | |  |  |  |  |  |  |  |  |
| 67 | ＞5 years | sheep | the growing season | MG | 3 |  | -0.3967 |  |  | |  |  |  |  |  |  |  |  |
| 67 | ＞5 years | sheep | the growing season | MG | 3 |  | -0.1687 |  |  | |  |  |  |  |  |  |  |  |
| 67 | ＞5 years | sheep | the growing season | MG | 3 |  | -0.3253 |  |  | |  |  |  |  |  |  |  |  |
| 67 | ＞5 years | sheep | the growing season | MG | 3 |  | -0.1669 |  |  | |  |  |  |  |  |  |  |  |
| 67 | ＞5 years | sheep | the growing season | MG | 3 |  | -0.2762 |  |  | |  |  |  |  |  |  |  |  |
| 67 | ＞5 years | sheep | the growing season | MG | 3 |  | -0.1718 |  |  | |  |  |  |  |  |  |  |  |
| 67 | ＞5 years | sheep | the growing season | MG | 3 |  | -0.0744 |  |  | |  |  |  |  |  |  |  |  |
| 67 | ＞5 years | sheep | the growing season | MG | 3 |  | -0.2933 |  |  | |  |  |  |  |  |  |  |  |
| 67 | ＞5 years | sheep | the growing season | MG | 3 |  | -0.1652 |  |  | |  |  |  |  |  |  |  |  |
| 67 | ＞5 years | sheep | the growing season | MG | 3 |  | -0.1546 |  |  | |  |  |  |  |  |  |  |  |
| 67 | ＞5 years | sheep | the growing season | MG | 3 |  | -0.3308 |  |  | |  |  |  |  |  |  |  |  |
| 67 | ＞5 years | sheep | the growing season | MG | 3 |  | 0.0157 |  |  | |  |  |  |  |  |  |  |  |
| 67 | ＞5 years | sheep | the growing season | MG | 3 |  | -0.1177 |  |  | |  |  |  |  |  |  |  |  |
| 67 | ＞5 years | sheep | the growing season | MG | 3 |  | 0.0245 |  |  | |  |  |  |  |  |  |  |  |
| 67 | ＞5 years | sheep | the growing season | MG | 3 |  | -0.0483 |  |  | |  |  |  |  |  |  |  |  |
| 67 | ＞5 years | sheep | the growing season | HG | 3 | -0.7411 | -0.867 | -0.1259 | -0.8642 | |  |  |  |  |  |  |  |  |
| 67 | ＞5 years | sheep | the growing season | HG | 3 | -0.5059 | -0.1723 | 0.3336 | -0.1821 | |  |  |  |  |  |  |  |  |
| 67 | ＞5 years | sheep | the growing season | HG | 3 | -0.2631 | -0.3806 | -0.1175 | -0.3747 | |  |  |  |  |  |  |  |  |
| 67 | ＞5 years | sheep | the growing season | HG | 3 | -0.7332 | -0.2224 | 0.5109 | -0.2508 | |  |  |  |  |  |  |  |  |
| 67 | ＞5 years | sheep | the growing season | HG | 3 | -0.7239 | -0.516 | 0.2079 | -0.5272 | |  |  |  |  |  |  |  |  |
| 67 | ＞5 years | sheep | the growing season | HG | 3 |  | -0.9606 |  |  | |  |  |  |  |  |  |  |  |
| 67 | ＞5 years | sheep | the growing season | HG | 3 |  | -0.2306 |  |  | |  |  |  |  |  |  |  |  |
| 67 | ＞5 years | sheep | the growing season | HG | 3 |  | -0.4263 |  |  | |  |  |  |  |  |  |  |  |
| 67 | ＞5 years | sheep | the growing season | HG | 3 |  | -0.3113 |  |  | |  |  |  |  |  |  |  |  |
| 67 | ＞5 years | sheep | the growing season | HG | 3 |  | -0.5864 |  |  | |  |  |  |  |  |  |  |  |
| 67 | ＞5 years | sheep | the growing season | HG | 3 |  | -0.8899 |  |  | |  |  |  |  |  |  |  |  |
| 67 | ＞5 years | sheep | the growing season | HG | 3 |  | -0.0182 |  |  | |  |  |  |  |  |  |  |  |
| 67 | ＞5 years | sheep | the growing season | HG | 3 |  | -0.317 |  |  | |  |  |  |  |  |  |  |  |
| 67 | ＞5 years | sheep | the growing season | HG | 3 |  | -0.126 |  |  | |  |  |  |  |  |  |  |  |
| 67 | ＞5 years | sheep | the growing season | HG | 3 |  | -0.6001 |  |  | |  |  |  |  |  |  |  |  |
| 67 | ＞5 years | sheep | the growing season | HG | 3 |  | -0.4532 |  |  | |  |  |  |  |  |  |  |  |
| 67 | ＞5 years | sheep | the growing season | HG | 3 |  | -0.1334 |  |  | |  |  |  |  |  |  |  |  |
| 67 | ＞5 years | sheep | the growing season | HG | 3 |  | -0.2204 |  |  | |  |  |  |  |  |  |  |  |
| 67 | ＞5 years | sheep | the growing season | HG | 3 |  | 0.0631 |  |  | |  |  |  |  |  |  |  |  |
| 67 | ＞5 years | sheep | the growing season | HG | 3 |  | -0.0219 |  |  | |  |  |  |  |  |  |  |  |
| 67 | ＞5 years | sheep | the growing season | EG | 3 | -0.9228 | -0.8017 | 0.1211 | -0.8041 | |  |  |  |  |  |  |  |  |
| 67 | ＞5 years | sheep | the growing season | EG | 3 | -0.7021 | -0.4203 | 0.2817 | -0.4288 | |  |  |  |  |  |  |  |  |
| 67 | ＞5 years | sheep | the growing season | EG | 3 | -0.3861 | -0.5733 | -0.1872 | -0.5636 | |  |  |  |  |  |  |  |  |
| 67 | ＞5 years | sheep | the growing season | EG | 3 | -0.9041 | -0.5083 | 0.3958 | -0.5315 | |  |  |  |  |  |  |  |  |
| 67 | ＞5 years | sheep | the growing season | EG | 3 | -0.8312 | -0.7488 | 0.0824 | -0.7535 | |  |  |  |  |  |  |  |  |
| 67 | ＞5 years | sheep | the growing season | EG | 3 |  | -0.9339 |  |  | |  |  |  |  |  |  |  |  |
| 67 | ＞5 years | sheep | the growing season | EG | 3 |  | -0.5145 |  |  | |  |  |  |  |  |  |  |  |
| 67 | ＞5 years | sheep | the growing season | EG | 3 |  | -0.648 |  |  | |  |  |  |  |  |  |  |  |
| 67 | ＞5 years | sheep | the growing season | EG | 3 |  | -0.6125 |  |  | |  |  |  |  |  |  |  |  |
| 67 | ＞5 years | sheep | the growing season | EG | 3 |  | -0.8611 |  |  | |  |  |  |  |  |  |  |  |
| 67 | ＞5 years | sheep | the growing season | EG | 3 |  | -0.8233 |  |  | |  |  |  |  |  |  |  |  |
| 67 | ＞5 years | sheep | the growing season | EG | 3 |  | -0.2659 |  |  | |  |  |  |  |  |  |  |  |
| 67 | ＞5 years | sheep | the growing season | EG | 3 |  | -0.6025 |  |  | |  |  |  |  |  |  |  |  |
| 67 | ＞5 years | sheep | the growing season | EG | 3 |  | -0.5495 |  |  | |  |  |  |  |  |  |  |  |
| 67 | ＞5 years | sheep | the growing season | EG | 3 |  | -0.7366 |  |  | |  |  |  |  |  |  |  |  |
| 67 | ＞5 years | sheep | the growing season | EG | 3 |  | -0.3209 |  |  | |  |  |  |  |  |  |  |  |
| 67 | ＞5 years | sheep | the growing season | EG | 3 |  | -0.1904 |  |  | |  |  |  |  |  |  |  |  |
| 67 | ＞5 years | sheep | the growing season | EG | 3 |  | -0.2349 |  |  | |  |  |  |  |  |  |  |  |
| 67 | ＞5 years | sheep | the growing season | EG | 3 |  | 0.0112 |  |  | |  |  |  |  |  |  |  |  |
| 67 | ＞5 years | sheep | the growing season | EG | 3 |  | -0.2915 |  |  | |  |  |  |  |  |  |  |  |
| 68 | 1 year | sheep | annual | EG | 2 |  |  |  |  | | 0.6712 |  | 0.4394 | -0.3194 | 0.6807 | -0.0192 | 0.0966 | -0.4565 |
| 68 | 1 year | sheep | annual | EG | 2 |  |  |  |  | | 0.4595 |  | 0.7436 | 0.665 | 0.4744 | -0.0234 | 0.0767 | -0.3882 |
| 68 | 1 year | sheep | annual | EG | 2 |  |  |  |  | | 1.0986 |  | 0.4394 | 0.2827 | 1.0347 | -0.0401 | -0.0144 | -0.3187 |
| 68 | 1 year | sheep | annual | EG | 2 |  |  |  |  | | 0.7122 |  | 0.5511 | 0.1451 | 0.7207 | -0.0279 | 0.0491 |  |
| 68 | 1 year | sheep | annual | MG | 2 |  |  |  |  | | 0.2985 |  | 0.2436 | -0.1142 | 0.2458 | -0.0119 | 0.1479 | 0.2854 |
| 68 | 1 year | sheep | annual | MG | 2 |  |  |  |  | | 0 |  | 0.2436 | 0.1895 | 0 | -0.0107 | 0.1847 | 0.2003 |
| 68 | 1 year | sheep | annual | MG | 2 |  |  |  |  | | 0.0834 |  | 0 | 0.2966 | -0.0705 | 0.0024 | -0.0989 | 0.4204 |
| 68 | 1 year | sheep | annual | MG | 2 |  |  |  |  | | 0.1542 |  | 0.603 | -0.1178 | 0 | -0.0012 | 0 | 0.2186 |
| 68 | 1 year | sheep | annual | MG | 2 |  |  |  |  | | -0.087 |  | 0.1292 | 0.3548 | -0.2981 | 0.0121 | -0.0146 | 0.1348 |
| 68 | 1 year | sheep | annual | MG | 2 |  |  |  |  | | -0.2162 |  | 0.2436 | 0.6162 | -0.049 | 0.0133 | -0.0146 | 0.2803 |
| 68 | 1 year | sheep | annual | MG | 2 |  |  |  |  | | 1.346 |  | 0.9232 | 0.2052 | 1.0091 | -0.0438 | -0.0513 | 0.3362 |
| 68 | 1 year | sheep | annual | MG | 2 |  |  |  |  | | 1.0629 |  | 0.7102 | -0.9262 | 0.782 | -0.0302 | -0.0513 | 0.4572 |
| 68 | 1 year | sheep | annual | MG | 2 |  |  |  |  | | 1.0259 |  | 0.8071 | 0.0388 | 0.8438 | -0.0327 | 0.0071 | 0.583 |
| 68 | 1 year | sheep | annual | MG | 2 |  |  |  |  | | 0.6337 |  | 0.628 | 0.0102 | 0.4556 | -0.0193 | 0.0353 |  |
| 68 | 1 year | sheep | annual | MG | 2 |  |  |  |  | | 0.3528 |  | 0.3939 | -0.0311 | 0.206 | -0.0096 | 0.0423 |  |
| 68 | 1 year | sheep | annual | MG | 2 |  |  |  |  | | 0.3254 |  | 0.4091 | 0.2902 | 0.291 | -0.006 | -0.0366 |  |
| 69 | 2-5 years | mixed | annual | EG | 3 |  |  |  |  | | -1.9009 | -0.525 | 0.1046 | -0.3686 | -0.7546 |  |  |  |
| 69 | 2-5 years | mixed | annual | EG | 3 |  |  |  |  | | -0.4947 | -0.6999 | -0.1247 | -0.3072 | -0.7175 |  |  |  |
| 69 | 2-5 years | mixed | annual | EG | 3 |  |  |  |  | | -0.5547 | -0.0225 | -0.1279 | -0.3902 | -0.6524 |  |  |  |
| 69 | 2-5 years | mixed | the growing season | HG | 3 |  |  |  |  | | -0.4258 | -0.1053 | 0.0557 | -0.1514 | -0.3261 |  |  |  |
| 69 | 2-5 years | mixed | the growing season | HG | 3 |  |  |  |  | | 0.0466 | -0.0181 | -0.0851 | -0.0432 | -0.2918 |  |  |  |
| 69 | 2-5 years | mixed | the growing season | HG | 3 |  |  |  |  | | -0.0706 | 0.1094 | -0.079 | -0.0455 | -0.4197 |  |  |  |
| 69 | 2-5 years | mixed | non-growing season | MG | 3 |  |  |  |  | | -0.071 | 0.2007 | 0.0374 | 0.0294 | -0.1505 |  |  |  |
| 69 | 2-5 years | mixed | non-growing season | MG | 3 |  |  |  |  | | 0.07 | -0.0507 | -0.0622 | -0.0694 | -0.5929 |  |  |  |
| 69 | 2-5 years | mixed | non-growing season | MG | 3 |  |  |  |  | | -0.1265 | 0.0621 | -0.0639 | -0.077 | -0.5757 |  |  |  |
| 70 | 2-5 years | mixed | annual | EG | 3 | -2.3747 | -2.0085 | 0.3663 | -2.0456 | |  |  |  |  |  |  |  |  |
| 70 | 2-5 years | mixed | the growing season | HG | 3 | -0.7173 | -0.3671 | 0.3502 | -0.4028 | |  |  |  |  |  |  |  |  |
| 70 | 2-5 years | mixed | non-growing season | MG | 3 | -0.3887 | -0.2171 | 0.1716 | -0.236 | |  |  |  |  |  |  |  |  |
| 71 | ＞5 years | sheep | the growing season | LG | 3 |  |  |  |  | | 0.2041 |  | 0.6124 |  |  | 0.0449 |  | 0.1421 |
| 71 | ＞5 years | sheep | the growing season | LG | 3 |  |  |  |  | | 0.0422 |  | 0.6807 |  |  | -0.004 |  | -0.0591 |
| 71 | ＞5 years | sheep | the growing season | MG | 3 |  |  |  |  | | -0.269 |  | 0.8292 |  |  | -0.0299 |  | -0.2083 |
| 71 | ＞5 years | sheep | the growing season | MG | 3 |  |  |  |  | | 0.1264 |  | 0.342 |  |  | 0.0045 |  | 0.0812 |
| 71 | ＞5 years | sheep | the growing season | HG | 3 |  |  |  |  | | -0.2672 |  | 0.5077 |  |  | -0.0189 |  | -0.3911 |
| 71 | ＞5 years | sheep | the growing season | HG | 3 |  |  |  |  | | 0.002 |  | 0.5807 |  |  | 0.0006 |  | -0.0288 |
| 72 | ＞5 years | sheep | the growing season | LG | 3 |  |  |  |  | | -0.0722 |  | 0.4475 |  |  |  |  |  |
| 72 | ＞5 years | sheep | the growing season | LG | 3 |  |  |  |  | | -0.1606 |  | 0.358 |  |  |  |  |  |
| 72 | ＞5 years | sheep | the growing season | LG | 3 |  |  |  |  | | 0.0426 |  | 0.3475 |  |  |  |  |  |
| 72 | ＞5 years | sheep | the growing season | MG | 3 |  |  |  |  | | -0.0654 |  | 0.4356 |  |  |  |  |  |
| 72 | ＞5 years | sheep | the growing season | MG | 3 |  |  |  |  | | -0.1382 |  | 0.3067 |  |  |  |  |  |
| 72 | ＞5 years | sheep | the growing season | MG | 3 |  |  |  |  | | -0.0354 |  | 0.0904 |  |  |  |  |  |
| 72 | ＞5 years | sheep | the growing season | HG | 3 |  |  |  |  | | -0.1138 |  | 0.5253 |  |  |  |  |  |
| 72 | ＞5 years | sheep | the growing season | HG | 3 |  |  |  |  | | -0.2151 |  | 0.175 |  |  |  |  |  |
| 72 | ＞5 years | sheep | the growing season | HG | 3 |  |  |  |  | | -0.1102 |  | -0.0858 |  |  |  |  |  |
| 73 | ＞5 years | mixed | annual | LG | 3 |  |  |  |  | | -0.0941 | -0.0552 | 0.3167 | 0.0402 | -0.0872 | 0.0093 |  |  |
| 73 | ＞5 years | mixed | annual | LG | 3 |  |  |  |  | | -0.0834 | -0.0812 | 0.3964 | 0.095 | -0.2218 | 0.0184 |  |  |
| 73 | ＞5 years | mixed | annual | LG | 3 |  |  |  |  | | -0.0973 | -0.0071 | 0.2854 | 0.0535 | -0.0467 | 0.0013 |  |  |
| 73 | ＞5 years | mixed | annual | LG | 3 |  |  |  |  | | -0.0441 | -0.0173 | 0.5967 | 0.0372 | -0.1265 | 0.0079 |  |  |
| 73 | ＞5 years | mixed | annual | LG | 3 |  |  |  |  | | -0.1264 | -0.0747 | 0.3222 | 0.0884 | -0.2 | 0.0039 |  |  |
| 73 | ＞5 years | mixed | annual | LG | 3 |  |  |  |  | | -0.1164 | -0.1354 | 0.2803 | 0.0287 | -0.1892 | -0.0013 |  |  |
| 73 | ＞5 years | mixed | annual | MG | 3 |  |  |  |  | | -0.1625 | -0.084 | 0.2064 | -0.009 | -0.5307 | 0.0468 |  |  |
| 73 | ＞5 years | mixed | annual | MG | 3 |  |  |  |  | | -0.1155 | -0.157 | 0.3951 | -0.0242 | -0.607 | 0.0617 |  |  |
| 73 | ＞5 years | mixed | annual | MG | 3 |  |  |  |  | | -0.2471 | -0.1536 | 0.2836 | -0.021 | -0.1732 | 0.0145 |  |  |
| 73 | ＞5 years | mixed | annual | MG | 3 |  |  |  |  | | -0.2373 | -0.2155 | 0.6431 | -0.0324 | -0.2375 | 0.026 |  |  |
| 73 | ＞5 years | mixed | annual | MG | 3 |  |  |  |  | | -0.3086 | -0.1085 | 0.1923 | -0.0222 | -0.8295 | 0.013 |  |  |
| 73 | ＞5 years | mixed | annual | MG | 3 |  |  |  |  | | -0.2284 | -0.1567 | 0.1193 | -0.0861 | -0.8014 | 0.0154 |  |  |
| 73 | ＞5 years | mixed | annual | HG | 3 |  |  |  |  | | -0.2247 | -0.1576 | 0.4557 | -0.1358 | -0.7286 | 0.0732 |  |  |
| 73 | ＞5 years | mixed | annual | HG | 3 |  |  |  |  | | -0.4869 | -0.1915 | 0.5756 | -0.0775 | -0.9602 | 0.0741 |  |  |
| 73 | ＞5 years | mixed | annual | HG | 3 |  |  |  |  | | -0.354 | -0.1886 | 0.5467 | -0.0565 | -0.4311 | 0.0744 |  |  |
| 73 | ＞5 years | mixed | annual | HG | 3 |  |  |  |  | | -0.2407 | -0.3308 | 0.8885 | -0.1988 | -0.5685 | 0.0725 |  |  |
| 73 | ＞5 years | mixed | annual | HG | 3 |  |  |  |  | | -0.5151 | -0.2669 | 0.5351 | -0.2234 | -0.9446 | 0.0472 |  |  |
| 73 | ＞5 years | mixed | annual | HG | 3 |  |  |  |  | | -0.6212 | -0.2128 | 0.6125 | -0.2759 | -1.085 | 0.0468 |  |  |
| 74 | ＞5 years | sheep | the growing season | LG | 3 | -0.1151 | -0.502 | -0.3313 | -0.4448 | |  |  |  |  |  |  |  |  |
| 74 | ＞5 years | sheep | the growing season | LG | 3 |  | -0.3953 |  |  | |  |  |  |  |  |  |  |  |
| 74 | ＞5 years | sheep | the growing season | LG | 3 |  | -0.335 |  |  | |  |  |  |  |  |  |  |  |
| 74 | ＞5 years | sheep | the growing season | LG | 3 |  | -0.5977 |  |  | |  |  |  |  |  |  |  |  |
| 74 | ＞5 years | sheep | the growing season | LG | 3 |  | -0.4464 |  |  | |  |  |  |  |  |  |  |  |
| 74 | ＞5 years | sheep | the growing season | MG | 3 | 0.8455 | -0.1608 | -1.0597 | -0.2067 | |  |  |  |  |  |  |  |  |
| 74 | ＞5 years | sheep | the growing season | MG | 3 |  | -0.1759 |  |  | |  |  |  |  |  |  |  |  |
| 74 | ＞5 years | sheep | the growing season | MG | 3 |  | -0.641 |  |  | |  |  |  |  |  |  |  |  |
| 74 | ＞5 years | sheep | the growing season | MG | 3 |  | 0.1882 |  |  | |  |  |  |  |  |  |  |  |
| 74 | ＞5 years | sheep | the growing season | MG | 3 |  | -0.2141 |  |  | |  |  |  |  |  |  |  |  |
| 74 | ＞5 years | sheep | the growing season | HG | 3 | 0.7665 | -0.0248 | -0.7374 | 0.0333 | |  |  |  |  |  |  |  |  |
| 74 | ＞5 years | sheep | the growing season | HG | 3 |  | 0.0722 |  |  | |  |  |  |  |  |  |  |  |
| 74 | ＞5 years | sheep | the growing season | HG | 3 |  | 0.0106 |  |  | |  |  |  |  |  |  |  |  |
| 74 | ＞5 years | sheep | the growing season | HG | 3 |  | 0.3315 |  |  | |  |  |  |  |  |  |  |  |
| 74 | ＞5 years | sheep | the growing season | HG | 3 |  | 0.029 |  |  | |  |  |  |  |  |  |  |  |
| 75 | 2-5 years | sheep | non-growing season | LG | 3 | 0.5837 |  |  |  | |  |  |  |  |  |  |  |  |
| 75 | 2-5 years | sheep | non-growing season | LG | 3 | 0.6521 |  |  |  | |  |  |  |  |  |  |  |  |
| 75 | 2-5 years | sheep | non-growing season | LG | 3 | 0.0777 |  |  |  | |  |  |  |  |  |  |  |  |
| 75 | 2-5 years | sheep | non-growing season | LG | 3 | 0.1915 |  |  |  | |  |  |  |  |  |  |  |  |
| 75 | 2-5 years | sheep | non-growing season | LG | 3 | 0.3454 |  |  |  | |  |  |  |  |  |  |  |  |
| 75 | 2-5 years | sheep | non-growing season | MG | 3 | 0.7243 |  |  |  | |  |  |  |  |  |  |  |  |
| 75 | 2-5 years | sheep | non-growing season | MG | 3 | 0.6297 |  |  |  | |  |  |  |  |  |  |  |  |
| 75 | 2-5 years | sheep | non-growing season | MG | 3 | 0.4552 |  |  |  | |  |  |  |  |  |  |  |  |
| 75 | 2-5 years | sheep | non-growing season | MG | 3 | 0.2495 |  |  |  | |  |  |  |  |  |  |  |  |
| 75 | 2-5 years | sheep | non-growing season | MG | 3 | 0.1593 |  |  |  | |  |  |  |  |  |  |  |  |
| 75 | 2-5 years | sheep | non-growing season | HG | 3 | 0.782 |  |  |  | |  |  |  |  |  |  |  |  |
| 75 | 2-5 years | sheep | non-growing season | HG | 3 | 0.4864 |  |  |  | |  |  |  |  |  |  |  |  |
| 75 | 2-5 years | sheep | non-growing season | HG | 3 | 0.1735 |  |  |  | |  |  |  |  |  |  |  |  |
| 75 | 2-5 years | sheep | non-growing season | HG | 3 | 0.0582 |  |  |  | |  |  |  |  |  |  |  |  |
| 75 | 2-5 years | sheep | non-growing season | HG | 3 | 0.0544 |  |  |  | |  |  |  |  |  |  |  |  |
| 76 | 2-5 years | mixed | annual | LG | 3 | -0.4428 |  |  |  | | -0.0791 | 0 | 0.1999 | 0.5457 |  | -0.0346 | 0.0335 |  |
| 76 | 2-5 years | mixed | annual | MG | 3 | -0.7036 |  |  |  | | -0.1146 | 0.277 | 0.4532 | 0.8361 |  | -0.0354 | 0.6741 |  |
| 76 | 2-5 years | mixed | annual | HG | 3 | -0.9391 |  |  |  | | -0.1355 | 0.2607 | 0.7997 | 0.0249 |  | 0.096 | 0.1441 |  |
| 77 | 2-5 years | sheep | the growing season | LG | 3 | 0.131 | -0.4797 | -0.6923 | -0.2546 | |  |  |  |  |  |  |  |  |
| 77 | 2-5 years | sheep | the growing season | LG | 3 |  | 0.2558 |  |  | |  |  |  |  |  |  |  |  |
| 77 | 2-5 years | sheep | the growing season | LG | 3 |  | 0.0151 |  |  | |  |  |  |  |  |  |  |  |
| 77 | 2-5 years | sheep | the growing season | LG | 3 |  | -0.3469 |  |  | |  |  |  |  |  |  |  |  |
| 77 | 2-5 years | sheep | the growing season | MG | 3 | 0.2288 | -0.2112 | -0.3183 | -0.1234 | |  |  |  |  |  |  |  |  |
| 77 | 2-5 years | sheep | the growing season | MG | 3 |  | -0.1005 |  |  | |  |  |  |  |  |  |  |  |
| 77 | 2-5 years | sheep | the growing season | MG | 3 |  | 0.367 |  |  | |  |  |  |  |  |  |  |  |
| 77 | 2-5 years | sheep | the growing season | MG | 3 |  | -0.159 |  |  | |  |  |  |  |  |  |  |  |
| 77 | 2-5 years | sheep | the growing season | HG | 3 | -0.1642 | -0.3719 | -0.336 | -0.2436 | |  |  |  |  |  |  |  |  |
| 77 | 2-5 years | sheep | the growing season | HG | 3 |  | 0.1799 |  |  | |  |  |  |  |  |  |  |  |
| 77 | 2-5 years | sheep | the growing season | HG | 3 |  | -0.034 |  |  | |  |  |  |  |  |  |  |  |
| 77 | 2-5 years | sheep | the growing season | HG | 3 |  | -0.2816 |  |  | |  |  |  |  |  |  |  |  |
| 78 | 2-5 years | sheep | annual | LG | 3 |  |  |  |  | | -0.0923 |  |  |  |  | 0.0299 | -0.04 | -0.0859 |
| 78 | 2-5 years | sheep | annual | LG | 3 |  |  |  |  | | 0.0393 |  |  |  |  | -0.0323 | 0.1953 | -0.2329 |
| 78 | 2-5 years | sheep | annual | LG | 3 |  |  |  |  | | 0.02 |  |  |  |  | 0.0301 | 0.0681 | 0.0126 |
| 78 | 2-5 years | sheep | annual | LG | 3 |  |  |  |  | | -0.0238 |  |  |  |  | 0.0273 | 0.0548 | -0.1299 |
| 78 | 2-5 years | sheep | annual | LG | 3 |  |  |  |  | | -0.1019 |  |  |  |  | 0.0135 | 0.0244 | 0.0241 |
| 78 | 2-5 years | sheep | annual | LG | 3 |  |  |  |  | | 0.0135 |  |  |  |  | 0.0135 | -0.0124 | -0.0419 |
| 78 | 2-5 years | sheep | annual | MG | 3 |  |  |  |  | | 0.0947 |  |  |  |  | 0.0401 | 0.2729 | -0.1978 |
| 78 | 2-5 years | sheep | annual | MG | 3 |  |  |  |  | | 0.0853 |  |  |  |  | 0.0538 | -0.0883 | -0.0609 |
| 78 | 2-5 years | sheep | annual | MG | 3 |  |  |  |  | | 0.0058 |  |  |  |  | 0.0162 | -0.0506 | 0.0634 |
| 78 | 2-5 years | sheep | annual | HG | 3 |  |  |  |  | | -0.0459 |  |  |  |  | 0.0225 | 0.0755 | -0.2885 |
| 78 | 2-5 years | sheep | annual | HG | 3 |  |  |  |  | | -0.0342 |  |  |  |  | 0.043 | 0.2729 | -0.2174 |
| 78 | 2-5 years | sheep | annual | HG | 3 |  |  |  |  | | -0.0118 |  |  |  |  | 0.0427 | 0.1318 | -0.09 |
| 78 | 2-5 years | sheep | annual | HG | 3 |  |  |  |  | | -0.0394 |  |  |  |  | 0.0413 | 0.18 | -0.1648 |
| 78 | 2-5 years | sheep | annual | HG | 3 |  |  |  |  | | -0.0702 |  |  |  |  | 0.0135 | 0.0715 | 0.0008 |
| 78 | 2-5 years | sheep | annual | HG | 3 |  |  |  |  | | -0.1413 |  |  |  |  | 0.0162 | -0.0124 | -0.0517 |
| 79 | 2-5 years | cattle | the growing season | MG | 6 | -0.6051 | -0.5258 | -0.2149 | -0.7707 | |  |  |  |  |  |  |  |  |
| 79 | 2-5 years | cattle | the growing season | MG | 6 |  | -1.6155 |  |  | |  |  |  |  |  |  |  |  |
| 79 | 2-5 years | cattle | the growing season | MG | 6 |  | -1.515 |  |  | |  |  |  |  |  |  |  |  |
| 79 | 2-5 years | mixed | annual | EG | 6 | -1.1494 | -1.369 | -0.2606 | -1.349 | |  |  |  |  |  |  |  |  |
| 79 | 2-5 years | mixed | annual | EG | 6 |  | -1.514 |  |  | |  |  |  |  |  |  |  |  |
| 79 | 2-5 years | mixed | annual | EG | 6 |  | -1.4207 |  |  | |  |  |  |  |  |  |  |  |
| 79 | 2-5 years | sheep | the growing season | LG | 3 |  |  |  |  | | 0.2851 | 0.1178 |  |  | 0.1907 | 0.0094 | -0.0517 |  |
| 79 | 2-5 years | sheep | the growing season | LG | 3 |  |  |  |  | | 0.203 | 0.0308 |  |  | 0.1196 | -0.0314 | 0.0084 |  |
| 79 | 2-5 years | sheep | the growing season | LG | 3 |  |  |  |  | | 0.0141 | 0.1335 |  |  | 0.049 | 0.0067 | -0.0079 |  |
| 79 | 2-5 years | sheep | the growing season | LG | 3 |  |  |  |  | | 0.1075 | 0 |  |  | 0.1594 | -0.0122 | 0 |  |
| 79 | 2-5 years | sheep | the growing season | LG | 3 |  |  |  |  | | 0.1409 | 0.1823 |  |  | 0.135 | -0.0154 | -0.0083 |  |
| 79 | 2-5 years | sheep | the growing season | LG | 3 |  |  |  |  | | 0.0575 | -0.069 |  |  | 0.0304 | -0.0573 | -0.0167 |  |
| 79 | 2-5 years | sheep | the growing season | LG | 3 |  |  |  |  | | -0.0778 | -0.2384 |  |  | -0.1556 | 0.0154 | 0.1236 |  |
| 79 | 2-5 years | sheep | the growing season | LG | 3 |  |  |  |  | | 0.3941 | 0.3321 |  |  | 0.3636 | 0.0056 | -0.0267 |  |
| 79 | 2-5 years | sheep | the growing season | MG | 3 |  |  |  |  | | -0.196 | -0.1335 |  |  | -0.102 | -0.0067 | 0.0884 |  |
| 79 | 2-5 years | sheep | the growing season | MG | 3 |  |  |  |  | | -0.2284 | -0.0984 |  |  | -0.2188 | -0.0652 | 0.0729 |  |
| 79 | 2-5 years | sheep | the growing season | MG | 3 |  |  |  |  | | 0.0619 | 0 |  |  | -0.0692 | -0.1022 | -0.0239 |  |
| 79 | 2-5 years | sheep | the growing season | MG | 3 |  |  |  |  | | 0.0071 | -0.0741 |  |  | -0.1409 | -0.0413 | 0.031 |  |
| 79 | 2-5 years | sheep | the growing season | MG | 3 |  |  |  |  | | 0.1856 | -0.069 |  |  | 0.2089 | 0.0056 | -0.0336 |  |
| 79 | 2-5 years | sheep | the growing season | MG | 3 |  |  |  |  | | 0.0699 | 0.1252 |  |  | -0.044 | -0.0544 | 0.0082 |  |
| 79 | 2-5 years | sheep | the growing season | MG | 3 |  |  |  |  | | 0.0135 | -0.2007 |  |  | -0.1608 | 0.0929 | 0.1158 |  |
| 79 | 2-5 years | sheep | the growing season | MG | 3 |  |  |  |  | | 0.091 | -0.1292 |  |  | 0.0718 | 0.112 | 0.1313 |  |
| 79 | 2-5 years | sheep | the growing season | HG | 3 |  |  |  |  | | -0.1343 | 0 |  |  | -0.0191 | 0.0239 | -0.0084 |  |
| 79 | 2-5 years | sheep | the growing season | HG | 3 |  |  |  |  | | 0.046 | 0.0606 |  |  | 0.0288 | -0.0314 | 0.0249 |  |
| 79 | 2-5 years | sheep | the growing season | HG | 3 |  |  |  |  | | 0.1929 | 0.1335 |  |  | 0.3054 | -0.0484 | -0.0484 |  |
| 79 | 2-5 years | sheep | the growing season | HG | 3 |  |  |  |  | | 0.1075 | -0.2412 |  |  | 0.0087 | -0.0655 | -0.0079 |  |
| 79 | 2-5 years | sheep | the growing season | HG | 3 |  |  |  |  | | 0.0941 | -0.0339 |  |  | 0.1083 | -0.0558 | -0.0251 |  |
| 79 | 2-5 years | sheep | the growing season | HG | 3 |  |  |  |  | | 0.1579 | -0.069 |  |  | 0.2169 | -0.0618 | 0.0325 |  |
| 79 | 2-5 years | sheep | the growing season | HG | 3 |  |  |  |  | | 0.2081 | -0.0953 |  |  | -0.0213 | 0.0428 | 0.1765 |  |
| 79 | 2-5 years | sheep | the growing season | HG | 3 |  |  |  |  | | 0.1857 | -0.2384 |  |  |  | -0.0099 | 0.076 |  |
| 80 | 1 year | mixed | the growing season | LG | 3 | -0.2645 |  | 0.5332 | 0.0531 | |  |  |  |  |  |  |  |  |
| 80 | 1 year | mixed | the growing season | LG | 3 | -0.1732 |  | 0.7386 | 0.061 | |  |  |  |  |  |  |  |  |
| 80 | 1 year | mixed | the growing season | LG | 3 | -0.2042 |  | 0.6049 | -0.0478 | |  |  |  |  |  |  |  |  |
| 80 | 1 year | mixed | the growing season | LG | 3 | -0.3636 |  | 1.4906 | -0.1772 | |  |  |  |  |  |  |  |  |
| 80 | 1 year | mixed | the growing season | LG | 3 | -0.2427 |  | 0.9475 | -0.1629 | |  |  |  |  |  |  |  |  |
| 80 | 1 year | mixed | the growing season | MG | 3 | -0.5682 |  | 0.3143 | -0.3893 | |  |  |  |  |  |  |  |  |
| 80 | 1 year | mixed | the growing season | MG | 3 | -0.4256 |  | 0.4068 | -0.3112 | |  |  |  |  |  |  |  |  |
| 80 | 1 year | mixed | the growing season | MG | 3 | -0.5381 |  | 0.3572 | -0.4542 | |  |  |  |  |  |  |  |  |
| 80 | 1 year | mixed | the growing season | MG | 3 | -0.6036 |  | 0.3965 | -0.575 | |  |  |  |  |  |  |  |  |
| 80 | 1 year | mixed | the growing season | MG | 3 | -0.5224 |  | 0.351 | -0.5005 | |  |  |  |  |  |  |  |  |
| 80 | 1 year | mixed | the growing season | HG | 3 | -0.956 |  | 0.0041 | -0.9538 | |  |  |  |  |  |  |  |  |
| 80 | 1 year | mixed | the growing season | HG | 3 | -0.8887 |  | 0.1334 | -0.8548 | |  |  |  |  |  |  |  |  |
| 80 | 1 year | mixed | the growing season | HG | 3 | -0.9225 |  | -0.6025 | -1.0193 | |  |  |  |  |  |  |  |  |
| 80 | 1 year | mixed | the growing season | HG | 3 | -1.1218 |  | 0.8025 | -1.051 | |  |  |  |  |  |  |  |  |
| 80 | 1 year | mixed | the growing season | HG | 3 | -0.9627 |  | 0.3526 | -0.9408 | |  |  |  |  |  |  |  |  |
| 81 | ＞5 years | mixed | annual | LG | 3 | -0.4468 | 0.0985 | 0.5855 | 0.1268 | |  |  |  |  |  |  | -0.4712 |  |
| 81 | ＞5 years | mixed | annual | LG | 3 |  | 0.1968 |  |  | |  |  |  |  |  |  | -0.3391 |  |
| 81 | ＞5 years | mixed | annual | LG | 3 |  | 0.1188 |  |  | |  |  |  |  |  |  |  |  |
| 81 | ＞5 years | mixed | annual | LG | 3 |  | 0.1722 |  |  | |  |  |  |  |  |  |  |  |
| 81 | ＞5 years | mixed | annual | LG | 3 |  | 0.2051 |  |  | |  |  |  |  |  |  |  |  |
| 81 | ＞5 years | mixed | annual | MG | 3 | -0.8199 | 0.0394 | 0.7512 | -0.0829 | |  |  |  |  |  |  | -1.0478 |  |
| 81 | ＞5 years | mixed | annual | MG | 3 |  | -0.0002 |  |  | |  |  |  |  |  |  |  |  |
| 81 | ＞5 years | mixed | annual | MG | 3 |  | -0.2027 |  |  | |  |  |  |  |  |  |  |  |
| 81 | ＞5 years | mixed | annual | MG | 3 |  | -0.2635 |  |  | |  |  |  |  |  |  |  |  |
| 81 | ＞5 years | mixed | annual | MG | 3 |  | -0.3959 |  |  | |  |  |  |  |  |  |  |  |
| 81 | ＞5 years | mixed | annual | HG | 3 | -2.1613 | 0.0418 | 2.0512 | -0.1335 | |  |  |  |  |  |  |  |  |
| 81 | ＞5 years | mixed | annual | HG | 3 |  | 0.008 |  |  | |  |  |  |  |  |  |  |  |
| 81 | ＞5 years | mixed | annual | HG | 3 |  | -0.4892 |  |  | |  |  |  |  |  |  |  |  |
| 81 | ＞5 years | mixed | annual | HG | 3 |  | -0.3674 |  |  | |  |  |  |  |  |  |  |  |
| 81 | ＞5 years | mixed | annual | HG | 3 |  | -0.3714 |  |  | |  |  |  |  |  |  |  |  |
| 82 | 2-5 years | cattle | the growing season | LG | 3 | -0.4833 |  |  |  | | -0.1365 |  |  |  |  |  |  |  |
| 82 | 2-5 years | cattle | the growing season | LG | 3 | -0.3561 |  |  |  | | -0.0669 |  |  |  |  |  |  |  |
| 82 | 2-5 years | cattle | the growing season | LG | 3 | -0.4716 |  |  |  | | -0.0572 |  |  |  |  |  |  |  |
| 82 | 2-5 years | cattle | the growing season | MG | 3 | -0.3188 |  |  |  | | -0.1027 |  |  |  |  |  |  |  |
| 82 | 2-5 years | cattle | the growing season | MG | 3 | -0.5636 |  |  |  | | -0.0669 |  |  |  |  |  |  |  |
| 82 | 2-5 years | cattle | the growing season | MG | 3 | -0.7612 |  |  |  | | -0.0733 |  |  |  |  |  |  |  |
| 82 | 2-5 years | cattle | the growing season | HG | 3 | -0.3266 |  |  |  | | -0.07 |  |  |  |  |  |  |  |
| 82 | 2-5 years | cattle | the growing season | HG | 3 | -0.6982 |  |  |  | | -0.0881 |  |  |  |  |  |  |  |
| 82 | 2-5 years | cattle | the growing season | HG | 3 | -0.7501 |  |  |  | | -0.0204 |  |  |  |  |  |  |  |
| 83 | ＞5 years | sheep | annual | MG | 3 |  |  |  |  | | 0.123 | -0.0378 | 0.0655 | -0.0461 | -0.0845 | 0.0314 |  | -0.1894 |
| 83 | ＞5 years | sheep | annual | MG | 3 |  |  |  |  | | 0.1438 | -0.0718 | -0.5348 | -0.2155 | -0.0728 | 0.0753 |  | 0.0825 |
| 83 | ＞5 years | sheep | annual | MG | 3 |  |  |  |  | | -0.3664 | -0.3462 | -0.1362 | 0.2657 | -0.2396 | 0.1542 |  | -0.3998 |
| 83 | ＞5 years | sheep | annual | MG | 3 |  |  |  |  | | -0.1688 | -0.394 | -0.3733 | -0.4532 | -0.0855 | 0.1171 |  | -0.348 |
| 83 | ＞5 years | sheep | annual | MG | 3 |  |  |  |  | | -0.1897 | -0.1025 | 0.6239 | -0.0221 | -0.1552 | 0.1082 |  | -0.1483 |
| 83 | ＞5 years | sheep | annual | MG | 3 |  |  |  |  | | -0.1795 | -0.1415 | 0.2533 | -0.85 | -0.1298 | 0.2393 |  | -0.0384 |
| 84 | ＞5 years | mixed | the growing season | EG | 3 |  |  |  |  | | -0.0741 | 0.1457 | -0.0576 | -0.0955 |  |  |  |  |
| 84 | ＞5 years | mixed | the growing season | EG | 3 |  |  |  |  | | 0.2364 | 0.2278 | 0.0075 | 0.0488 |  |  |  |  |
| 84 | ＞5 years | mixed | the growing season | EG | 3 |  |  |  |  | | 0.3365 | 0.1542 | -0.0074 | 0.1465 |  |  |  |  |
| 85 | ＞5 years | mixed | the growing season | MG | 3 | -0.7678 |  |  |  | | -0.3076 | -0.108 |  |  | -0.3266 |  | 0.2025 |  |
| 85 | ＞5 years | mixed | the growing season | MG | 3 |  |  |  |  | | -0.42 | -0.1684 |  |  | -0.3751 |  | 0.1484 |  |
| 85 | ＞5 years | mixed | the growing season | MG | 3 |  |  |  |  | | -0.3699 | -0.12 |  |  | -0.3202 |  | 0.0918 |  |
| 85 | ＞5 years | mixed | the growing season | MG | 3 |  |  |  |  | | -0.3052 | -0.1116 |  |  | -0.285 |  | 0.0651 |  |
| 85 | ＞5 years | mixed | the growing season | MG | 3 |  |  |  |  | | -0.1746 | -0.1394 |  |  | -0.2317 |  | 0.0274 |  |
| 86 | 2-5 years | mixed | annual | EG | 3 | -0.803 |  |  |  | | -0.3007 | -0.1691 |  |  |  |  | 0.1324 | -0.08 |
| 86 | 2-5 years | mixed | annual | EG | 3 |  |  |  |  | | -0.0359 | -0.0488 |  |  |  |  | 0.0085 | -0.0182 |
| 86 | 2-5 years | mixed | annual | EG | 3 |  |  |  |  | | -0.0238 | -0.0645 |  |  |  |  | 0.0079 | -0.0185 |

Appendix S1. A list of 86 articles from which data were extracted for the meta-analysis

1 Sun, Y. et al. Responses of soil ammonia oxidizers and denitrifiers to different grazing intensities. Acta Ecologica Sinica (2018).

2 Wang, X. et al. Effects of grazing on nitrogen transformation in swamp meadow wetland soils in Napahai of Northwest Yunnan. Acta Ecologica Sinica (2018).

3 Zang, X. et al. Effect on functional diversity of Artemisia frigida rhizosphere soil microbial community with grazing. Journal of Zhejiang A & F University (2017).

4 Feng, X. C., Zhang, L., Ya-Ru, L. I., Bai, S. & Taogetao, B. Effects of Seasonal Grazing on Turnover of Carbon and Nitrogen in Soil Microorganism in Semi-arid Typical Grassland. Chinese Journal of Grassland (2017).

5 Jin, J. et al. Soil nitrogen and Stipa krylovii roots in desert steppe in response to different grazing treatments. Chinese Journal of Ecology (2017).

6 Li, H. Q. et al. Effects of grazing intensity on the ecological stoichiometry characteristics of alpine meadow. Pratacultural Science (2017).

7 Wang, S. S., Zhao, Y. G., Shi, Y. F., Gao, L. Q. & Yang, Q. Y. Impact of short-term grazing disturbance on nitrogen accumulation of biological soil crusts in the hilly Loess Plateau region, China. The journal of applied ecology 28, 3848 (2017).

8 Yao Z et al. Effects of different restoration measures on storage of soil organic carbon and nitrogen in typical steppe of the Loess Hilly Area in Ningxia. Acta Prataculturae Sinica 26, 236-242 (2017).

9 Han, M. et al. Response of Species Diversity and Productivity to Long-term Grazing in the Stipa breviflora Desert Steppe. Acta Botanica Boreali-Occidentalia Sinica (2017).

10 Zhang, S. et al. Study on Aboveground Biomass and Vegetation Stability of Main Plant Populations and Community in Stipa breviflora Desert Steppe. Chinese Journal of Grassland (2017).

11 Hai, L., Wang, S. F., Zhi-Gang, Y. U. & Wang, X. J. Influence of Grazing on Plant Community Characteristics in Typical Mountainous Area in Wulashan,Inner Mongolia. Journal of Inner Mongolia Forestry Science & Technology (2016).

12 Liu, Z. et al. Effects of Different Land Use Patterns on Soil Inorganic Carbon in Alpine Meadow Ecosystem. Bulletin of Soil & Water Conservation (2016).

13 Dong, W. et al. Effect of rest-grazing management on soil water and carbon storage in an arid grassland (China). Journal of Hydrology 527, 754-760 (2015).

14 Zhou, Z. C., Gan, Z. T., Shangguan, Z. P. & Dong, Z. B. Effects of grazing on soil physical properties and soil erodibility in semiarid grassland of the Northern Loess Plateau (China). Catena 82, 87-91 (2010).

15 Wang, D., Wu, G. L., Zhu, Y. J. & Shi, Z. H. Grazing exclusion effects on above- and below-ground C and N pools of typical grassland on the Loess Plateau (China). Catena 123, 113-120 (2014).

16 Xing, W. et al. Effects of grazing exclusion on soil carbon and nitrogen storage in semi-arid grassland in Inner Mongolia, China. Chinese Geographical Science 24, 479-487 (2014).

17 Zhang, J. N. et al. Response of plant diversity and soil nutrient condition to grazing disturbance in Stipa baicalensis Roshev. grassland. Acta Agrestia Sinica 18, 177-182 (2010).

18 Guo, M. Y. et al. Effect of grazing on grassland soil respiration. Pratacultural Science (2011).

19 Lintuya, X. I., Zhu, X. U. & Zheng, Y. Effects of Grazing Intensity on Soil Physical and Chemical Properties of Inner Mongolia Grassland. Prataculture & Animal Husbandry (2009).

20 Tu-Ya, X., Zhu, X. U. & Yang, Z. Influence of Different Stocking Rates on Underground Biomass and Net Primary Productivity on Stipa krylovii Steppe in Inner Mongolia. Chinese Journal of Grassland (2009).

21 Liu YJ & YJ, Z. Effect of Different Grazing Intensities on Vegetation and Soil Physical and chemical Character. Acta Botanica Boreali-Occidentalia Sinica 7, 137-139 (2009).

22 Wang HY et al. Effects of different grazing intensities on total and light fraction organic carbon and nitrogen storages of soil in Stipa Grandis steppe. Journal of Soil and Water Conservation 29, 101-106 (2015).

23 Sa RGW & Ao TG. The Effects of Different Grazing Intensities on the Vegetation, Soil and Livestock Gain Weight in the Inner Mongolia Typical Steppe Journal of Inner Mongolia Agricultural University 8, 113-119 (2011).

24 Liu, X. M. Effects on Soil Properties and Aboveground Biomass of Desert Grassland on Different Grazing Intensities. Grass-Feeding Livestock (2017).

25 Yang, H. S. et al. Influence of Different Grazing Intensity to Soil Fertility in the Subei Alpine Steppes. Journal of Soil & Water Conservation (2009).

26 Zhen-Sheng, S. U. et al. Effects of grazing intensity on soil nutrient of Kobresia pygmaea meadow in Tibet Plateau. Pratacultural Science 32, 322-328 (2015).

27 Mi ZY & Wang Mj. Soil Nitrogen Mineralization of Stipa breviflora Desert steppe Under Different Grazing Intensities and Managing Methods. Journal of Inner Mongolia Agricultural University 6, 89-93 (2011).

28 Wang, H. et al. Organic carbon storage properties in Stipa breviflora desert steppe vegetation soil systems under different grazing intensities. Acta Ecologica Sinica (2016).

29 Wang, T. L. et al. Study on Changes of Soil Nutrients and Plant Community of Stipa breviflora Steppe under Different Grazing Intensities. Acta Agrestia Sinica (2017).

30 Gu WR & Zhu JZ. Effects of Seasonal Delaying Grazing on Vegetation and Soil under Different Grazing Intensities Xinjiang Agricultural Sciences 8, 931-937 (2013).

31 Lan, R., Guo, J., Yin, Z., Jinrong, L. I. & Zhang, T. Effects of Soil Erosion on the Soil Organic Carbon of Typical Steep Under Different Grazing Intensities. Journal of Soil & Water Conservation (2017).

32 E ER & M, H. Study of soil physical properties and water characteristics change under different grazing intensities. Modern Agriculture 3, 90-92 (2012).

33 Ren J et al. Effect of Different Grazing Time on Under-ground Biomass of Plant Communities in Desert Steppe. Animal Husbandry and Feed Science 10, 32-35 (2016).

34 Wang, H. Y. et al. Organic Carbon Storage Characteristics of Stipagrandis Typical Steppe in Different Grazing Degradation Degree. Chinese Journal of Grassland (2016).

35 Li LC, Zhao ML & Han, G. D. The Characteristics of Soil Organic Carbon and the Relationships between Soil Organic Carbon and Vegetations in Desert Steppe under Different Grazing Gradients. Journal of Arid Land Resources and Environment 5, 134-138 (2008).

36 Chang Y, Sun SX, Wang M & Wei ZJ. Effects of the Different Grazing Systems on Vegetation Charaeteristics and Soil in Stipa Breviflora Desert Steppe Grassland and Prataculture 25, 38-42 (2013).

37 Hai-Hong, X. U., Hou, X. Y. & Ri-Su, N. A. Dynamics of soil respiration under different grazing systems in a Stipa breviflora desert steppe. Acta Prataculturae Sinica (2011).

38 Fan, C. et al. Study on Effects of Different Grazing Systems on Phosphorus Loss in Surface Runoff in Hulunbair Grasslands. Journal of Soil & Water Conservation (2017).

39 Wei LY, Wei QZ, Mo ZP & Tong DW. Effects of Different Disturbances on Species Diversity and Productivity of the Shrub-Grass Vegetation on the Degraded Land in Northwest Guangxi, China. Research of Soil and Water Conservation 23, 288-293 (2016).

40 Luo, L. M. et al. Effects of Disturbance Intensity on the Community Characteristics and Functional Traits of Meadow Steppe at a Valley of the Lhasa River Basin. Acta Agrestia Sinica (2015).

41 Zhen, W. et al. Effects of land use on biomass of dominant plants in typical steppe. Acta Prataculturae Sinica (2016).

42 Xuan, Q. et al. Effects of different land-use types on soil active organic carbon in the Stipa klemenaii desert steppe of Inner Mongolia. Acta Prataculturae Sinica (2016).

43 Yu Hongqian & Xu Dongmai. Effect of different grazing ways on soil physical and chemical properties and carbon balance in steppe desert. Ningxia university (2014).

44 Wei Yinjie & Wei ZJ. Response of Vegetation and Soil Physical and Chemical Properties to Grazing Systems in Stipa grandis Steppe. Journal of Inner Mongolia Agricultural University (2012).

45 Bing, L. Responses of soil biological properties to different grazing managements in damxung alpine meadow. University of science and technology of China (2013).

46 REN Ling et al. Characteristics of soil nutrients in alpine meadow under different utilization patterns in Eastern Qilian Mountains. Journal of Gansu Agricultural University 51, 70-75 (2016).

47 WANG Tian - le, WEI Zhi - jun, Lu Shi jie, LIU Weng - ting & ting, B. Y.-. The Response of Soil Physical and Chemical Properties to Seasonal Regulation of Grazing Intensity in Stipa breviflora Steppe. Animal Husbandry and Feed Science 38, 25-31 (2017).

48 Ding, H. et al. Effect of stocking rate on Stipa breviflora desert steppe soil. Chinese Journal of Eco-Agriculture (2016).

49 Han, M. Q. et al. Response of soil nitrogen mineralization to different stocking rates on the Stipa breviflora desert steppe. Acta Prataculturae Sinica (2017).

50 Li, L. Q. et al. Effects of rest-grazing in the regreen-up period on moderately degraded steppification meadow of Qilian Mountain. Pratacultural Science (2017).

51 Liu, Y. et al. Effects of Grazing on Community and Soil Characteristics in the Semi-arid Grassland. Acta Botanica Boreali-Occidentalia Sinica (2016).

52 Li Y & Han, G. D. Effects of Grazing on the Grassland Carbon Storage of the Stipa Grandis Steppe. Journal of Inner Mongolia Agricultural University (2011).

53 Wen, Y. Effect of grazing on soil fertility and phosphorus availability in the red soil region, northeast yunnan. Acta Pedologica Sinica 45, 569-572 (2008).

54 Zhang Chengxia & Nan Zhibiao. Effects of grazing on soil physical chaemistry characteristics and microbes at losse plateau, China. Lanzhou university (2008).

55 SHENG Haiyan, ZHANG Chun ping, CAO Guangming & Guangfen, Z. Effect of grazing on soil environment of alpine meadow dominated by Potentilla froticosa shrub on Qilian Mountain. Ecology and Environmental Sciences 18, 1088-1093 (2009).

56 makefei, X. et al. Response of Plant Diversity and Soil Nutrient to Grazing Intensity in Kobresia pygmaea Meadow of Qinghai-Tibet Plateau. Acta Agrestia Sinica 20, 1026-1032 (2012).

57 Mipam, T. D., Wen, Y. L., Ai, Y., Zhao, H. W. & Chen, Y. J. Impact of different grazing intensity on soil physical properties and plant biomass in Qinghai-Tibet Plateau alpine meadow ecosystem. Pratacultural Science (2016).

58 Zhang, Q. Q. et al. Impact of grazing on soil δ~(15)N of mountainous grassland ecosystems over the northern Tianshan Mountains,China. Pratacultural Science (2016).

59 Jing, Z. Effect of Grazing on Carbon and Nitrogen Reserve of Tibet Alpine Typical Wetland. Hubei Agricultural Sciences 55, 4660-4663 (2016).

60 FengCheng, Z. Effect of grazing on vegetation biomass and soil charater in deyeuxia angustifolia meadow of sanjiang plain. Heilongjiang Animal Science and Veterinary Medicine 12, 67-71 (2012).

61 Liu, S. Y., Cui, G. W., Niu, Z., Wang, Y. Q. & Wen-Hua, H. E. Effects of Grazing on Population Characteristics of Deyeuxia angustifolia and Content of Main Soil Nutrients. Chinese Journal of Grassland (2015).

62 Yao, G., Gao, Y., Yang, T., Ding, Y. & Shilong, M. A. The influence of grazing intensities on litter storage and vegetation productivity of Stipa klemenzii desert steppe. Journal of Arid Land Resources & Environment (2016).

63 Zhang, H., Shi, S. & Wang, S. Effect of grazing intensities on plant community structure and grassland productivity in desert steppe of Ningxia. Journal of Arid Land Resources & Environment (2012).

64 Guang-Peng, Q. U., Mu-You, C., Zhao, J. X. & Tian, L. H. Responses of communitycharacteristics,soil carbon and nitrogen to different grazingmanagement in Tibet alpine wetlands. Grassland & Turf (2016).

65 Wen, L. I., Cao, W. X., Xiao-Long, L. I., Chang-Lin, X. U. & Shi, S. L. Effect of different grazing management on soil nutrient characteristics in alpine meadow-steppe. Grassland & Turf (2016).

66 Wen, L. I. et al. Changes in organic carbon and nitrogen storage in alpine meadows under different grazing management regimes. Acta Prataculturae Sinica (2016).

67 Dun, S. S., Cao, J. R., Jia, X. & Pang, S. Effects of grazing and mowing on extractable carbon and nitrogen in typical grassland of Inner Mongolia, China. Chinese Journal of Applied Ecology 28, 3235-3242 (2017).

68 Du, Y. F. et al. Effects of grazing on nitrogen contents in rhizosphere and non-rhizosphere soil of Stipa breviflora. Pratacultural Science (2016).

69 Wei XiaoJun & Hong, M. The Effort of Grazing Intensity to Nutrition of Stipa Grandis Rhizosphere which is The Mainly Built The Plants of Typical Steppe. Journal of Inner Mongolia Agricultural University (2011).

70 Dong, Q. M. et al. Study on grazing yak performance and soil nutrient changes in warm-season pastures of alpine region. Acta Agrestia Sinica 17, 629-635 (2009).

71 Shao-Wei, M. A. et al. Effect of Grazing Intensity on Stipa breviflora Communities and Canopy Interception. Chinese Journal of Grassland (2016).

72 Li, H. Q. et al. Effects of grazing intensity on the ecological stoichiometry characteristics of alpine meadow. Pratacultural Science (2017).

73 Tan, Y. R. et al. Impact of grazing on the activities of soil enzymes and soil nutrient factors in an alpine meadow on the Qinghai-Tibetan plateau. Journal of Lanzhou University (2012).

74 Lin, L. et al. Responses of soil nutrient traits to grazing intensities in alpine Kobresia meadows. Acta Ecologica Sinica 36 (2016).

75 ZHAI Wenting et al. Effects of Grazing Intensity on Carbon Metabolic Characteristics of Soil Microbial Communities in Alpine Grassland in the Surrounding Area of Qinghai Lake Chin J Appl Environ Biol 23, 0685-0692 (2017).

76 Lv, P. et al. Effects of Grazing Intensity on Vegetation in Sandy Grassland of Horqin. Journal of Desert Research (2016).

77 Yang, Y. et al. Effects of fencing on vegetation community characteristics and soil properties of a typical steppe in Inner Mongolia. Acta Prataculturae Sinica (2016).

78 Sun, X., Ding, W., Jia, H. T. & Jin, J. X. Effect of simulated grazing on carbon storage of meadow grassland ecosystem in the north slope of Tianshan Mountain. Pratacultural Science (2016).

79 Anqier, B. Effect of marketers Grazing Intensities on Grassland Carbon Density and Storage of Carbon of Leymus chinensis Meadow Steppe. Journal of Inner Mongolia Agricultural University (2011).

80 Wei B., Ni L. U., Jiaqi L. I., Zhao, M., & Yingwen, Y. U.. Effects of Enclosure on Plant Community Composition and Niche Characteristics in Alpine Meadow. Acta Botanica Boreali-Occidentalia Sinica, 37 (5) : 0983-0991 (2017).

81 Mao S, Qihua W U, Hongqin L I, et al. Effects of grazing intensity on species diversity and biomass in alpine-cold forb meadow on the Tibetan Plateau[J]. Journal of Glaciology & Geocryology, 37 (5) : 1372-1380 (2015).

82 Yang, H. L. et al. Effects of short-period grazing on soil active organic carbon fractions in Zhaosu meadow steppe. Pratacultural Science 30, 1926-1932 (2013).

83 Shuai, Z. Effects of grazing and fenced on soil microbial diversity in stipa steppes of hulunbeier, inner mongolia. Chinese academy of agricultural sciences (2011).

84 Liu, X. D. et al. Effects of grazing and fencing on nutrients and enzyme activities in desert steppe soil. Acta Agriculturae Zhejiangensis (2016).

85 Chen, F. et al. Effects of different disturbances on diversity and biomass of communities in the typical steppe of loess region. Acta Ecologica Sinica 33, 2856-2866 (2013).

86 Qiu, L. H., Qin, J. H. & Zhang, Y. Effect of banning grazing on alpine meadow soil organic carbon,physio-chemical properties and enzyme activities in Binggou Watershed,Qilian Mountains. Agricultural Research in the Arid Areas (2017).
